# Oncogenic and tumor-suppressive forces converge on a progenitor-orchestrated niche to shape early tumorigenesis

**DOI:** 10.1101/2025.06.10.656791

**Published:** 2025-06-12

**Authors:** José Reyes, Isabella Del Priore, Andrea C. Chaikovsky, Nikhita Pasnuri, Ahmed M. Elhossiny, Tobias Krause, Andrew Moorman, Catherine Snopkowski, Meril Takizawa, Cassandra Burdziak, Nalin Ratnayeke, Ignas Masillioni, Yu-Jui Ho, Ronan Chaligné, Paul B. Romesser, Aveline Filliol, Tal Nawy, John P. Morris, Zhen Zhao, Marina Pasca Di Magliano, Direna Alonso-Curbelo, Dana Pe’er, Scott W. Lowe

**Affiliations:** 1 Cancer Biology and Genetics Program, Memorial Sloan-Kettering Cancer Center, New York, NY, USA; 2 Computational and Systems Biology Program, Memorial Sloan-Kettering Cancer Center, New York, NY, USA; 3 Louis V. Gerstner Jr. Graduate School of Biomedical Sciences, Memorial Sloan-Kettering Cancer Center, New York, NY, USA; 4 Single-cell Analytics Innovation Lab, Memorial Sloan-Kettering Cancer Center, New York, NY, USA; 5 Department of Computational Medicine and Bioinformatics, University of Michigan, Ann Arbor, MI, USA; 6 Department of Radiation Oncology, Memorial Sloan-Kettering Cancer Center, New York, NY, USA; 7 Department of Pharmacology, The University of North Carolina at Chapel Hill Medical School, Chapel Hill, NC, USA; 8 Lineberger Comprehensive Cancer Center, Chapel Hill, NC, USA; 9 Cold Spring Harbor Laboratory; New York, NY, USA; 10 Rogel Cancer Center, University of Michigan, Ann Arbor, MI, USA; 11 Department of Surgery, University of Michigan, Ann Arbor, MI, USA; 12 Institute for Research in Biomedicine (IRB Barcelona), The Barcelona Institute of Science and Technology (BIST), Barcelona, Spain; 13 Howard Hughes Medical Institute, Chevy Chase, MD, USA

## Abstract

The transition from benign to malignant growth is a pivotal yet poorly understood step in cancer progression that marks the shift from a pathologically inert condition to a clinically lethal disease. Here, we integrate lineage tracing, single-cell and spatial transcriptomics to visualize the molecular, cellular and tissue-level events that promote or restrain malignancy during the tumor initiation in mouse models of pancreatic ductal adenocarcinoma (PDAC). We identify a discrete progenitor-like population of *KRAS*-mutant cells that co-activates oncogenic and tumor-suppressive programs—including p53, CDKN2A, and SMAD4—engaging senescence-like responses and remodeling their microenvironment, ultimately assembling a niche that mirrors invasive PDAC. KRAS inhibition depletes progenitor-like cells and dismantles their niche. Conversely, p53 suppression enables progenitor cell expansion, epithelial–mesenchymal reprogramming, and immune-privileged niche formation. These findings position the progenitor-like state as the convergence point of cancer-driving mutations, plasticity, and tissue remodeling—revealing a critical window for intercepting malignancy at its origin.

## Introduction

Cancer progression is a multistep process fueled by the accumulation of genetic alterations that reshape cell identity and reprogram the tumor microenvironment. Yet, clonal expansions bearing oncogenic mutations rarely progress to cancer^1^, despite appearing frequently in histologically normal tissues^2,3^. These clones can persist for long periods as benign growths that retain epithelial architecture^4–6^. Only upon acquisition of additional genetic or epigenetic changes do these lesions breach regulatory constraints, promoting cellular plasticity, local invasion, and metastatic dissemination – features of lethal cancer. Despite its clinical importance, the transition from benign to malignant growth remains poorly understood, in part due to the difficulty of capturing and studying these early events *in vivo*.

Pancreatic ductal adenocarcinoma (PDAC) is an almost universally fatal malignancy^7,8^ that exemplifies progression through well-defined benign and malignant states. It is nearly universally initiated by activating mutations in *KRAS*^9^, which confer epithelial plasticity and drive the formation of pancreatic intraepithelial neoplasia (PanINs) and other precursor lesions^10^. The dense stroma of PDAC, characterized by activated fibroblasts, immunosuppressive myeloid cells, and cytotoxic T-cell exclusion, further shapes both tumor progression and therapeutic response^11–15^. Although mutant KRAS is sufficient to initiate PDAC development and remains necessary for progression and maintenance^16^, we do not understand how genetic lesions, cell state changes, and microenvironmental remodeling converge to trigger the benign to malignant transition.

Disruption of the *TP53*, *CDKN2A* and/or *SMAD4* tumor suppressor pathways contribute to the benign to malignant transition^9,17–20^. Among these, the gene encoding the sequence-specific transcription factor p53, which governs diverse tumor-suppressive molecular programs^21,22^, is mutated and/or deleted most frequently (roughly 70% of cases)^23^. *TP53* mutations are uncommon in low-grade PanINs^24^ and dispensable for their formation in mouse models^25^ and humans^24^. On the other hand, the frequency of *TP53* inactivation increases in high-grade lesions and carcinoma^26^, supporting the role for p53 in restraining malignant progression. Using genetically engineered mice, we previously showed that *Trp53* loss of heterozygosity (LOH) facilitates the ordered accumulation of copy number alterations (CNAs) that are analogous to those occurring in human PDAC^27^. Conversely, p53 restoration in advanced tumors induces reversion to a more differentiated, PanIN-like state^28^. These findings suggest that p53 safeguards against PDAC progression by limiting genomic instability and opposing cell state plasticity—but when, where, and how p53 and other tumor suppressors act during premalignancy to restrain malignant transformation remains unresolved.

In addition to genetic lesions, inflammatory cues are critical modulators of PDAC evolution. Chronic pancreatitis is a risk factor for PDAC^29^, and inflammation accelerates neoplastic progression in *Kras*-mutant mouse models^30^. In the normal pancreas, injury induces acinar-to-ductal metaplasia (ADM), followed by regeneration and restoration of tissue homeostasis^31^. However, this regenerative process is subverted in the presence of oncogenic KRAS, resulting in the establishment of heterogeneous ductal metaplasia, including acinar derived PanIN-like lesions^30–33^. Interestingly, oncogenic KRAS activity in mouse models of PDAC results in the formation of inflammatory niches in around neoplastic niches in the absence of experimentally-induced inflammation^34–36^, implying an inextricable link between oncogenic KRAS activity and the remodeling of the pancreatic microenvironment.

Our prior work revealed that inflammation synergizes with oncogenic KRAS to promote chromatin remodeling and the establishment of a subpopulation of premalignant cells expressing the *Nes*, a marker of pancreatic epithelial progenitor cells^37–39^. This progenitor-like state exhibits high plasticity, as evidenced by permissive chromatin landscapes that suggest that this state can transition into distinct premalignant and malignant states^37^. In addition, this state closely resembles invasive cancer both in terms of transcriptional and chromatin accessibility landscapes. This includes chromatin opening near cell-communication genes, implying a functional interplay between epithelial plasticity and the surrounding niche. Strikingly, this progenitor-like state arises rapidly after injury, while the malignant transition takes months, suggesting that additional tumor-suppressive barriers delay disease progression.

Here, we use lineage tracing and conditional p53 mouse models to dissect how genetic alterations, cell state transitions, and microenvironmental remodeling converge to drive early PDAC progression. By integrating single-cell and spatial -omics with targeted perturbations, we capture the dynamic interplay between epithelial plasticity and niche reprogramming during the benign-to-malignant transition. We directly visualize and reconstruct how this transition is orchestrated by spatially mapping the emergence, stabilization, and expansion of a plastic, progenitor-like cell state uniquely capable of engaging—and ultimately evading—tumor-suppressive mechanisms while assembling a supportive microenvironment for tumor evolution. These insights illuminate a critical window of vulnerability in tumorigenesis and provide a conceptual foundation for intercepting cancer at its inception by targeting the cell states and intercellular communication networks that enable malignant transformation.

## Results

### Capturing *p53*-deficient cell states after spontaneous *p53* loss of heterozygosity

**Figure 1.**
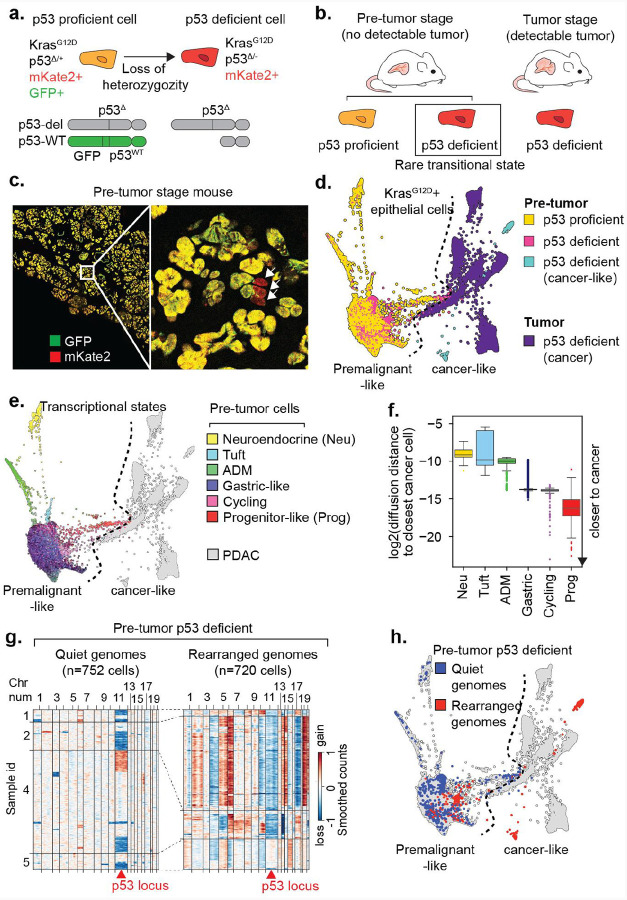
Capturing spontaneous loss of *p53* throughout the premalignant-to-malignant spectrum. **a**. KP^LOH^ mouse model. Loss of GFP linked to the only wild-type *p53* copy in the cell reports *p53* loss of heterozygosity. **b**. Sampling strategy to characterize progression from premalignant to malignant states. **c**. Representative fluorescence image of a pre-tumor stage pancreas section. Arrowheads point to rare cells that lost GFP fluorescence upon *p53* LOH. **d**. Force-directed layout (FDL) of single-cell transcriptional data from sorted *Kras*^G12D^ epithelial cells, colored by mouse stage and *p53* status. **e**. Projection of transcriptional signatures of major subpopulations identified in premalignant pancreas. Multiple transcriptional signatures were used to annotate cell type (Methods). ADM, acinar-to-ductal metaplasia. **f**. Diffusion distance from pre-tumor *p53*-proficient or *p53*-deficient cells to the closest cancer-like cell. **g**. Single-cell karyotypes of pre-tumor *p53*-deficient cells inferred from scRNA-seq (Methods). Rows represent individual cells and columns represent genes, ordered by genomic position. Colors represent inferred loss or gain of genomic material. Chr, chromosome. **h**. FDL of pre-tumor *p53*-deficient cells, colored by genomic state.

To investigate cellular and molecular events underlying the benign-to-malignant transition in pancreatic cancer, we employed the KP^LOH^ model^27^ that enables identification and isolation of cells that undergo spontaneous loss of heterozygosity (LOH) of *Trp53* (hereafter referred to as *p53*) during tumor initiation. The model is derived from multi-allelic KPC (*K**ras*^G12D^, *Tr**p**53*^flox/+^, Ptf1a-Cre) embryonic stem cells that incorporate dual fluorescent reporters to track mutant *Kras* and *p53* status: mKate for lineage-tracing *Kras*-mutant epithelium and GFP for marking cells that retain wild-type *p53* (Fig. 1a). In this system, *Kras*-mutant epithelial cells that retain wild-type *p53* are mKate2/GFP double-positive, whereas cells undergoing spontaneous *p53* LOH become mKate2 single-positive due to co-deletion of a physically linked GFP reporter. In mice lacking macroscopic PDAC (3–4 months old, hereby termed “pre-tumor” stage), *p53*-deficient (mKate2+/GFP-) cells are rare, appearing as isolated cells, small apparently clonal clusters, or as ‘microtumors’ histologically resembling advanced PDAC^27^ (Fig. 1b,c). By isolating tissue from mice well before the onset of detectable malignant tumors, this system facilitates visualization and characterization of epithelial cell states before and immediately after *p53* loss, as well as their interactions with the surrounding microenvironment.

Using single-cell RNA sequencing (scRNA-seq), we compared these rare single-positive cells that underwent *p53* LOH (pre-tumor *p53*-deficient, 1–3% of all mKate2+ cells) to their *p53*-proficient double-positive counterparts (pre-tumor p53 proficient), and to single positive cells isolated from full-blown tumors (tumor *p53*-deficient) (Fig. 1d, Fig. S1a–d and Methods). Consistent with prior work, *p53*-proficient premalignant cells occupied heterogeneous transcriptional states that departed from a normal acinar phenotype^37,40^ but were clearly distinct from malignant PDAC cells, which formed distinct, tumor-specific clusters (Fig. 1e,f and Fig. S1e–k). The *p53*-proficient fraction included cells undergoing acinar-to-ductal metaplasia (ADM) (expressing *Cpa1+, Krt19+*), cells with neuroendocrine (*Scg5+, Chga+, Chgb+*) or tuft (*Pou2f3+*) features, cells expressing gastric lineage markers characteristic of PanIN lesions and the classical PDAC subtype (*Dmbt1+, Muc6+, Tff1+, Tff2+, Anxa10+*), and cells displaying proliferation markers (*Mki67+*, *Cdk1+*) (Fig. 1e and Fig. S1e–k). Interestingly, a small subset of cells expressed the progenitor-like program previously identified as defining a transient, cancer-like cell state induced in *Kras*-expressing epithelium following tissue injury (e.g., *Nes+*, *Msn+, Hmga2+, Vim+*)^37^ (Fig. 1e and Fig. S1e–k). Diffusion distance analysis (see Methods and Note S1) revealed that among all pre-tumor p53 proficient cells, the progenitor-like population is transcriptionally closest to PDAC (Fig. 1f) – and thus a likely transitional intermediate in the benign-to-malignant transition.

We previously applied single-cell DNA sequencing to the KP^LOH^ model and demonstrated that *p53* loss is accompanied by a progressive and selective accumulation of genomic copy number alterations (CNAs) as cells acquire the genomic and histological features of invasive disease^27^. Reasoning that the gradual acquisition of CNAs in *p53*-deficient (mKate2 single-positive cells) could serve as a timestamp of a cell’s trajectory toward malignancy, we inferred CNAs from scRNA-seq data as a surrogate for underlying genomic instability. As expected, most pre-tumor *p53*-deficient cells occupying premalignant transcriptional states had quiet genomes, aside from loss of chromosome 11, indicating that these cells underwent *p53* LOH but had not yet acquired genomic instability or a malignant phenotype (Fig. 1e,g and Fig. S2a). At the other extreme, some *p53*-deficient cells displayed rampant genomic instability (rearranged genomes) and transcriptional profiles resembling PDAC. These microtumors are likely clonal expansions of malignant cells that are below the detection threshold for ultrasound or gross pathology but are evident upon detailed histological examination^27^ (Fig. S2b). Notably, such microtumors also expressed the progenitor state marker HMGA2 (Fig. S2b).

Some *p53*-deficient cells within the pre-tumor pancreata exhibited an intermediate level of genomic rearrangement, consistent with a transitional state in which *p53* LOH occurred but the additional genetic alterations required for full malignancy were still being acquired. Notably, many of these cells occupied the progenitor-like state (Fig. 1h and Fig. S2c) and some shared distinguishing karyotypic changes with highly rearranged, malignant-appearing cells from the same mouse—for example, harboring loss of chr4 (*Cdkn2a*), loss chr11 (*p53*) and gain of chr2, while retaining diploid status in other chromosomes altered in microtumor cells from the same sample (e.g., chr5, chr6, chr10, chr13, chr14) (Fig. S2c). Together, these data point to the highly plastic, progenitor-like state, as a likely precursor for malignant tumors.

### Rare progenitor-like premalignant cells exhibit peak activity of oncogenic and tumor suppressive programs

**Figure 2.**
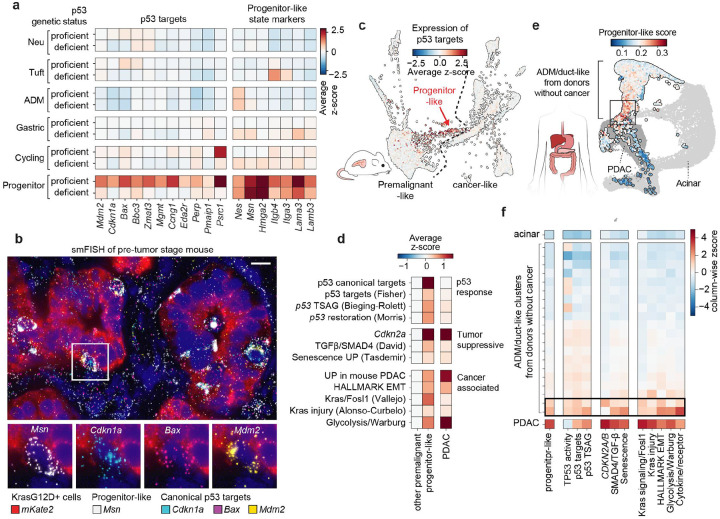
*p53* is maximally active in rare premalignant cells during tumor initiation. **a**. Expression of known p53 targets and markers of progenitor-like cells in pre-tumor *p53*-proficient and deficient cells, as a function of cell state. **b**. Representative smFISH image of pre-tumor stage pancreas, probing for p53 targets and the progenitor-like state marker *Msn*. **c**. FDL of *Kras*^G12D^-positive epithelial cells along PDAC progression, colored by p53 average expression of p53 canonical targets shown in (a). **d**. Expression of tumor-suppressive and oncogenic gene signatures in pre-tumor *p53*-proficient cells or tumor *p53*-deficient cells (PDAC). p53 canonical signature derived from p53 targets in Fig. 2a. Other signatures shown are: p53 curated targets (Fisher)^116^; p53 TSAG, tumor suppression–associated genes^117^; p53-restoration^28^; *Cdkn2a* mRNA; TGFβ-dependent SMAD4 targets^118^; HALLMARK EMT, epithelial-to-mesenchymal transition^119^; senescence UP^52^; UP in mouse PDAC (this work, see Methods); Kras/Fosl1^45^; Kras injury^38^; glycolysis/warburg (curated list, see Methods). **e**. UMAP of cells from healthy human pancreas and PDAC tissue in^3^, colored by progenitor-like signature. PDAC cells and acinar cells are grayed out to facilitate visualization of duct-like and ADM cells from donors with and without cancer. Box highlights cells that exhibit highest progenitor-like signatures. **f**. Expression of tumor-suppressive and oncogenic signatures in pancreatic epithelial cells with and without donors. Different rows of ADM/duct-like cells correspond to PhenoGraph clusters. Colors represent z-score of average signature scores in each column. Box highlights the two PhenoGraph clusters with highest progenitor-like signatures in cells from donors without cancer.

Our single cell data define a window to interrogate the molecular events that precede or immediately follow *p53* inactivation during early tumorigenesis. To identify how spontaneous *p53* loss impacts the distinct premalignant subpopulations, we compared expression of canonical p53 targets between p53-proficient and deficient cells within each transcriptionally-defined premalignant state. Surprisingly, *p53* inactivation had minimal impact on canonical p53 targets in most premalignant cell types, with one notable exception—progenitor-like cells (Fig. 2a, Fig. S3a and Methods). In *p53*-proficient contexts, this subpopulation expressed the highest levels of p53 targets, including genes involved in cell cycle arrest (e.g., *Cdkn1a, Ccng1*), DNA repair (e.g., *Mgmt*) and apoptosis (e.g., *Bbc3, Bax, Pmaip1*). These transcripts were downregulated in the corresponding *p53*-deficient cells, confirming their p53 dependence. Spatial mapping of these features using single molecule fluorescence in situ hybridization (smFISH) revealed that individual *Msn*-positive progenitor-like cells were dispersed throughout glandular structures of the premalignant pancreas, suggesting that this p53-active cell state arises independently during spontaneous tumorigenesis, and does not expand clonally in a p53 proficient setting (Fig. 2b). Thus, despite uniform *Kras*^*G12D*^ mutation across the epithelial compartment and loss of acinar identity^37^, p53 activity is heterogeneous and confined to progenitor-like cells—the subpopulation most transcriptionally related to PDAC (Fig. 2c).

Strikingly, in addition to p53 activation, progenitor-like cells exhibited the highest engagement of the two other major tumor suppressive programs in PDAC^23^: *CDKN2A*^41^ and SMAD4^42^ (Fig. 2d and Fig. S3b–d). Specifically, progenitor-like cells significantly upregulated *Cdkn2a* relative to other premalignant cells (Fig. S3c,d), and inspection of splice junctions in sequencing reads indicated that transcripts encoding both p19^ARF^ and p16^INK4A^ were induced (Fig. S3e), indicating that both tumor suppressive programs encoded by the *Cdkn2a* locus were engaged^19,41^. Additionally, gene set enrichment analysis identified the TGFβ pathway as significantly upregulated in progenitor-like cells as compared to other premalignant subpopulations, an effect that was also observed when using a curated list of SMAD4-dependent TGFβ-induced genes that have been established as SMAD4-dependent^43^ (Fig. S3b–d). Therefore, all three tumor suppressive programs that are commonly lost during PDAC progression are engaged in the progenitor-like state.

Regardless of *p53* status, progenitor-like cells preferentially upregulated gene expression programs associated with malignant PDAC, including KRAS signaling, glycolysis, and epithelial-mesenchymal transition (EMT)^38,44–46^ (Fig. 2d and Fig. S3b–d). The simultaneous engagement of tumor-promoting and tumor-suppressive pathways is reminiscent of oncogene-induced senescence, a potent tumor suppressive program involving p53 and p16^INK4a^ that can be triggered by aberrant RAS signaling^47–50^. Consistent with this, progenitor-like cells were enriched for senescence-associated transcriptional signatures (Fig. 2d and Fig. S3b–d).

To determine whether analogous cell states exist in the human pancreas, we reanalyzed scRNA-seq data from pancreatic epithelial cells obtained at warm autopsy from cancer-free individuals^3^. This dataset includes cells that encompass a spectrum of epithelial states, including normal acinar cells and ADM/duct-like populations. Projection of murine progenitor-like signatures onto these data revealed transcriptional alignment with a rare subset of ADM- and duct-like epithelial cells, which exhibited upregulation of hallmark PDAC programs – including KRAS signaling, glycolysis, and EMT– as well as p53 transcriptional signatures (Fig. 2e,f and Fig. S3f,g). While *KRAS* mutational status could not be inferred from these datasets, our analyses support the presence of an epithelial subpopulation in the human pancreas of cancer-free individuals that mirrors the progenitor-like state observed in mice. Together, these data highlight a conserved premalignant cell state in which oncogenic and tumor-suppressive programs intersect, revealing a potential battleground for malignant transformation.

### Adoption of progenitor-like identity is coupled with morphological reorganization of premalignant lesions

**Figure 3.**
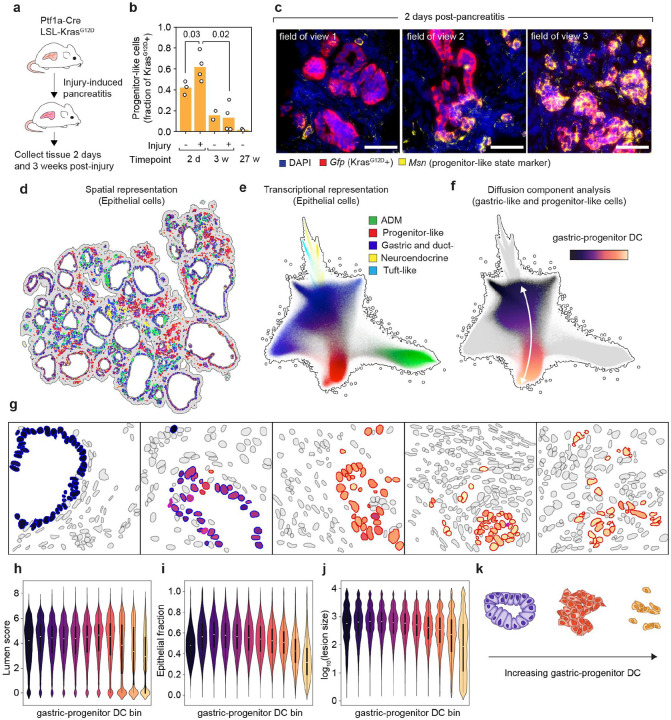
Transcriptional and morphological states undergo coordinated changes in the premalignant epithelium. **a**. Experimental timeline for tissue collection after inducing caerulein-induced pancreatitis in KC mice. **b**. Fraction of progenitor-like epithelial cells in scRNA-seq data as a function of treatment condition and time. **c**. Representative images of smFISH staining for the progenitor-like marker *Msn*. The three fields of view are from the same tissue. Scale bars, 50 μm. **d,e**. Spatial representation (d) or single-cell transcriptional embedding (e) of Xenium data annotated by signatures of major premalignant subpopulations. **f**. Projection of gastric–progenitor diffusion component in transcriptional embedding of single-cells derived from Xenium data. **g**. Representative fields of view of premalignant epithelial lesions in Xenium data. Segmented nuclei are pseudo-colored by their gastric–progenitor DC value, using the colormap in (f). **h–j**. Distributions of lumen score (h), epithelial fraction in local spatial neighborhood (i) and lesion size (j) as a function of gastric–progenitor DC in epithelial cells (see Methods for details on the definition and quantification of morphological parameters). **k**. Schematic of the changes in lesion morphologies along the gastric-progenitor DC continuum.

We and others previously identified progenitor-like cells as a highly plastic subpopulation with elevated cell–cell communication potential that expands following caerulein-induced pancreatitis in *Kras*-mutant mice^37,38,40^. These cells are rare in 12–27-week-old mice not subjected to injury (Figs. 1e, 3b and Fig. S1k), but transiently accumulate upon acute pancreatitis, reaching up to 60% of the epithelium, before progressively declining over 3 weeks (Fig. 3a,b)^37^. The expansion of the progenitor-like subpopulation coincides with other tissue remodeling events, including loss of acinar identity in the epithelial compartment and formation of a fibrotic niche in the stroma. Notably, as shown above, progenitor-like cells exhibit transcriptional features of senescence (see Fig. S3c,d)—a state known to contribute to tissue remodeling through secretory programs and bidirectional signaling^51–53^. These observations suggest that progenitor-like cells are not merely passive products of KRAS activation, but active participants in constructing the premalignant niche.

To study the spatiotemporal dynamics of premalignant lesions in response to pancreatic injury, we leveraged our ability to generate large and synchronous cohorts of KC (LSL-*Kras*^G12D^; *Ptf1a*-Cre) mice derived by injection of multiallelic mESCs into early embryos^54,55^. In addition, our models enable selective tracing of Kras-mutant epithelial cells by fluorescent reporters (mKate2 and GFP), allowing their identification and isolation during tumor initiation. Tissues were collected for histological analysis either 2 days or 3 weeks after the first caerulein injection (see Fig. 3a), time points that capture both the accumulation and depletion of progenitor-like cells in response to inflammation (Fig. 3b).

Consistent with our results from dissociated single-cell analyses (PMID: **37167403**), progenitor-like cells accumulated within 48h of injury-induced pancreatitis, as evidenced by upregulation of MSN, HMGA2, or both proteins (Fig. S4a), as well as the tumor suppressor proteins p53 and p19^ARF^ (Fig. S4a,b). Lesions enriched with progenitor-like cells (termed progenitor lesions) formed disorganized epithelial structures, in contrast with the rosette-like and luminal epithelial morphologies of premalignant tissue lacking this cellular state (Fig. 3c and Fig. S4a). More interestingly, progenitor lesions were morphologically diverse: within the same mouse, progenitor-like cells can appear as small, isolated clusters; as dominant tissue lesions separated by stroma; or as mixed lesions suggestive of transitional states (Fig. 3c). This variety of morphologies may represent snapshots of progressive epithelial identity loss during the earliest stages of oncogenic KRAS-driven transformation.

To systematically define the early morphological, molecular, and compositional underlying the emergence of progenitor lesions and their associated microenvironments (“progenitor niches”), we performed spatial transcriptomics using the Xenium In Situ platform (10x Genomics), which enables single cell resolution within intact tissue. Guided by prior scRNAseq data and smFISH^37,40^) analyses in the premalignant pancreas (Fig. S5), we designed a custom 480-gene panel enriched for epithelial markers to resolve premalignant heterogeneity, while also capturing stromal and immune features and and key signaling and communication pathways^37^ implicated in early tumorigenesis, including MAPK, p53, YAP, TGFβ, and interferon (**Table S4** and Methods). Applying this approach to KC mice (Ptf1a-Cre; LSL-KrasG12D) at early (1–2 days, *n* = 10 mice) and late (3 weeks, *n* = 5 mice) timepoints following caerulein-induced injury enabled spatial profiling across a continuum of progenitor lesions. For each of the 3,833,679 cells analyzed, we obtained precise spatial context and a phenotypic profile based on gene expression. Despite the targeted nature of the panel, we successfully applied established scRNA-seq analysis methods,– including UMAP embedding, clustering, cell type annotation, and diffusion component analysis–to resolve cell states (Fig. 3d–f).

Treating spatial and transcriptional data as complementary yet integrated dimensions allowed us to connect cell phenotypes with their native spatial context in the premalignant state. This analysis successfully discriminated a rich diversity of cell states and their spatial patterning across different length scales: from macroscopic tissue landmarks (e.g. lymphatics, lobular zones) to microscopic structures (e.g, epithelial lesions and blood vessels), and fine-grained gradients (e.g. fibroblast layering) (Fig. S6). The concordance between transcriptional state and spatial arrangement supports the notion that collective self-organization underlies the morphological and transcriptional patterning of the premalignant pancreas.

Initially focusing on the epithelial compartment, we recovered the major subpopulations previously identified by scRNA-seq^37,40^ (Figs. 1e and 3d,e and Methods). These data revealed continuous transcriptional gradients connecting these states, consistent with dynamic transitions between premalignant cell subpopulations (Fig. 3e). To characterize such gradients, we applied diffusion component analysis^56,57^ and identified the continuous axis connecting gastric- and progenitor-like states as the dominant axis of transcriptional variation in these data (hereby termed gastric-progenitor DC) (Fig. 3f and Methods). Gene expression changes along this axis revealed sequential programs marked by early *Msn* induction, followed by *Hmga2*, and culminating upregulation of *Vim*, a canonical mesenchymal cell marker (Fig. S4d).

The gastric-progenitor diffusion component (DC) provided a framework to quantify the morphological, compositional and molecular changes in premalignant lesions as epithelial cells acquired progenitor-like features. By ordering epithelial cells along this axis and analyzing the spatial distribution of epithelial nuclei (see Methods), we observed progressive loss of luminal architecture (Fig. 3g,h), reduced epithelial density accompanied with immune and stromal cell infiltration (Fig. 3g,i), and lesion shrinkage culminating in isolated progenitor-like cells embedded within stroma (Fig. 3g,j). These spatially-resolved analyses map how transcriptional reprogramming toward a progenitor-like state coincides with epithelial disorganization and niche remodeling during early lesion development (Fig. 3k). Together, these findings demonstrate that epithelial cells undergoing progenitor-like reprogramming progressively lose epithelial organization and adopt mesenchymal traits (Fig. S4c), defining a continuum of premalignant remodeling marked by transcriptional plasticity and early features of invasiveness—hallmarks of cancer cell dissemination in PDAC mouse models^58^.

### Progenitor niches resemble cancer niches

To elucidate how progenitor-like cells reshape their microenvironment, we extended our spatial analyses beyond the epithelium to investigate the surrounding stromal and immune compartments. PDAC is characterized by a profoundly remodeled microenvironment comprised of activated fibroblasts and immunosuppressive myeloid cells that suppress cytotoxic T-cell infiltration and limit therapeutic efficacy^14^. Myofibroblastic cancer-associated fibroblasts (myCAFs), defined by *Acta2*, *Postn*, *Tnc*, and *Tgfb1* expression^59^, contribute to extracellular matrix (ECM) deposition and promote tumor-supportive inflammation^60^. In parallel, monocytes and tumor-associated macrophages (TAMs) expressing markers such as *Arg1, Spp1*, and *Il1b*, are known to contribute immune suppression in PDAC and other malignancies^61–64^. Notably, these features mirror cellular programs engaged in wound repair^65,66^ and can arise early in tumorigenesis^36,67,68^, possibly as a regenerative response to incipient cancer cells.

**Figure 4.**
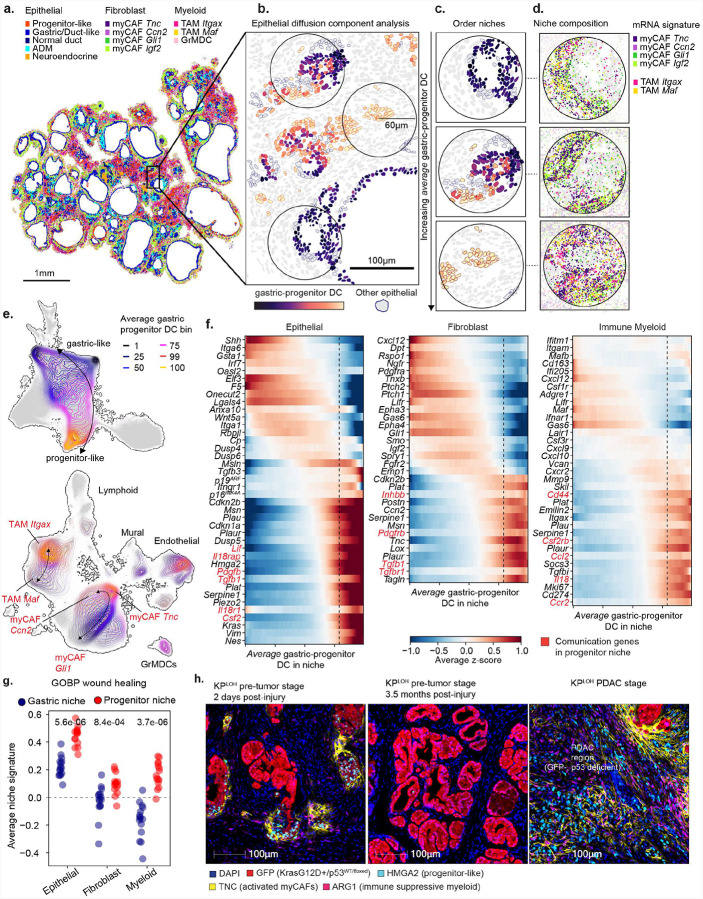
Continuous cellular and molecular remodeling events during the assembly of the progenitor niche. **a**. Representative section of premalignant pancreas harvested 2 days post-injury, analyzed using the Xenium platform and colored by cell type. **b**. Projection of gastric–progenitor DC in premalignant cells. Epithelial cells not categorized as gastric-like or progenitor-like are outlined in dark blue, but not pseudo-colored. **c**. Niches, comprising all cells within a 60-μm radius of a central anchor epithelial cell, are ordered by the average gastric–progenitor DC of their constituent epithelial cells. **d**. Location of individual mRNA molecules associated with select myofibroblasts and monocyte/macrophage subpopulations in niches depicted in (c). **e**. Contour plots denote the density of niche epithelial cells in select bins along the average gastric-progenitor DC (top) and corresponding shifts in the density of microenvironment cells (bottom). **f**. Average niche expression of select genes in (left) epithelial, (middle) fibroblast or (right) myeloid immune cells along the average gastric-progenitor DC. Niches are ordered by average gastric–progenitor DC value, divided into 100 equal bins. Dotted lines indicate DC value at which epithelial cells begin expressing progenitor-like markers. Communication genes associated with progenitor niches are highlighted in red. **g**. Average expression of wound-healing response genes (GO Biological Processes) from our Xenium panel. Each dot represents a single biological replicate (n=15 mice). Values denote the average z-scored expression of wound-healing genes in the specified cellular compartments, in either gastric or progenitor niches. P-values, two-tailed Wilcoxon Rank Sums Test. **h**. Immunofluorescence staining for cellular states enriched in the progenitor niche, as a function of PDAC progression. Scale bars, 100 μm.

To reconstruct the dynamics of niche formation, we leveraged the asynchronous nature of tissue remodeling captured in our spatial datasets. Each tissue section contained epithelial cells spanning the gastric-to-progenitor continuum, surrounded by diverse stromal and immune populations (Fig. S4d and Fig. S6). This spatial heterogeneity was not randomly distributed: epithelial cells within a given spatial neighborhood typically occupied similar positions along the gastric–progenitor axis, forming coherent regional patterns. We formalized these spatial neighborhoods by defining multicellular “niches” as all cells within a 60-μm radius of an epithelial cell ‘anchor’ (Fig. 4a,b). Calculating the average gastric-progenitor DC of niche epithelial cells, we positioned these niches along a pseudotime continuum of progenitor-like state acquisition (Fig. 4c), analogous to single-cell trajectory inference^56,69,70^. This framework allowed us to connect changes in epithelial transcriptional identity with progressive microenvironmental remodeling, revealing niche dynamics from static tissue snapshots.

To examine how the microenvironment evolves across this continuum, we first assessed cell type composition within spatial neighborhoods. This conventional approach revealed only modest differences across the gastric–progenitor axis (Fig. S7a), underestimating the differences between the niches that surround distinct premalignant subpopulations. However, when we instead compared the transcriptional profiles of microenvironmental cells at either end of the niche continuum, we observed stark differences in niche composition: niches dominated by gastric-like epithelial cells were predominantly surrounded by Shh-responsive *Gli1*+ myofibroblasts^71^ and *Maf+*^72^, whereas niches dominated by progenitor-like cells were embedded in a microenvironment enriched for *Itgax*+ (CD11c+) monocytes/macrophages and activated myCAFs expressing the injury-associated ECM component *Tnc*^73^ (Fig. 4d and Fig. S7b–e). These findings prompted us to move beyond discrete cell types to a cell-state based framework for mapping continuous microenvironmental changes along the niche continuum.

To resolve how the microenvironment changes along the gastric-progenitor continuum, we embedded all non-epithelial cells in UMAP space based on their transcriptomic profiles, and visualized their densities (Fig. 4e, bottom) as a function of the average gastric-progenitor DC of epithelial cells in their respective niche (Fig. 4e, top). This continuous, state-based framework revealed gradual and coordinated remodeling of the fibroblast and myeloid compartments, with progressive enrichment of *Itgax+* monocyte/macrophages and myCAFs expressing ECM and TGFβ-related genes (e.g., *Postn*, *Tgfb1*, *Tnc*) (Fig. 4f). Notably, myofibroblasts expressing the *Ccn1* and *Ccn2*, two members of the matricellular CCN family induced by YAP signaling, TGFb, and hypoxia, among other forms of stress^74^, were enriched around progenitor-like cells with advanced mesenchymal features (Fig. S7c), which localized to the periphery of pancreatic lobules (Fig. S6), indicating zonation of progenitor-like niches along the epithelial-mesenchymal plasticity axis. Therefore, niche remodeling is a progressive, spatially organized process tightly coupled to epithelial dedifferentiation.

Shifts along the gastric-progenitor niche continuum involved gradual, compartment-specific gene expressing changes (Fig. 4f), reinforcing the progressive, rather than binary, nature of niche remodeling. Analysis of dissociated single-cell datasets from injured KC mice revealed that the dominant axis of transcriptional variation in fibroblast or myeloid cells mirrored the changes along the niche trajectory (Fig. 4f, S7d,e), indicating that spatial context is a major source of transcriptional heterogeneity in the premalignant pancreas. This analysis also revealed that myeloid cells in progenitor niche expressed markers of immune suppressive subpopulations, including *Spp1*+, *Arg1*+ and *Il1b*+ cells^61,62,64^, while fibroblasts upregulated activation markers (*Acta2, Timp1, Tgfb1, Tnc*) and had features of senescent myofibroblasts (*Cdkn2a+, Cdkn2b+, Plaur+*) previously linked to PDAC progression^60^. Together, these spatially resolved trajectories reconstruct a pseudo-temporal sequence of niche remodeling culminating in the formation of the progenitor niche— a multicellular community defined by the presence of ARG1+ macrophages and TNC+ myofibroblasts (Fig. 4h) that is reminiscent of the desmoplastic and immune suppressive microenvironment of malignant PDAC^11^. Importantly, histologically advanced lesions spontaneously arising in the KP^LOH^ model exhibited similar stromal composition (Fig. 4h), implicating progenitor-like cells act as early architects of the malignant niche during sporadic tumorigenesis.

Given that progenitor-like cells display features of senescence (see Fig. S3c–d)—a state implicated in both tissue repair and fibrosis^75,76^ —we hypothesized that their emergence reflects an aberrant wound-healing response co-opted by oncogenic KRAS. Consistent with this possibility, we noted widespread induction of a wound-healing response signature across stromal and immune compartments in the progenitor niche (Fig. 4g). Among the most upregulated genes were those encoding secreted factors such as *Pgdfb* and *Tnfrsf12a* – mediators of inflammation and fibrosis^77,78^– alongside coordinated activation of the plasminogen pathway (e.g.m *Plaur*, *Plat*, *Plau*, *Serpine1*) (Fig. S7f). These findings suggest that progenitor-like cells initiate a conserved, multi-lineage wound-healing program that, when sustained in the context of oncogenic signaling, drives the assembly of tumor-permissive niches.

### Concerted activation of inter-cellular communication modules define the progenitor niche

The tight spatial and transcriptional coupling of distinct cell states within the progenitor niche suggested a coordinated assembly process driven by intercellular signaling. The first generation of computational approaches for inferring ligand-receptor interactions from single cell data, such as CellPhoneDB and NicheNet, relied on correlating ligand and receptor expression in dissociated single-cell data^79,80^, without accounting for spatial context. Given that the physical proximity of signaling partners is often important for functional signaling, we used spatial transcriptomics to impose an additional constraint: requiring cells expressing cognate ligands and receptors to be colocalized within the same niche–to define communication potential^79^.

**Figure 5.**
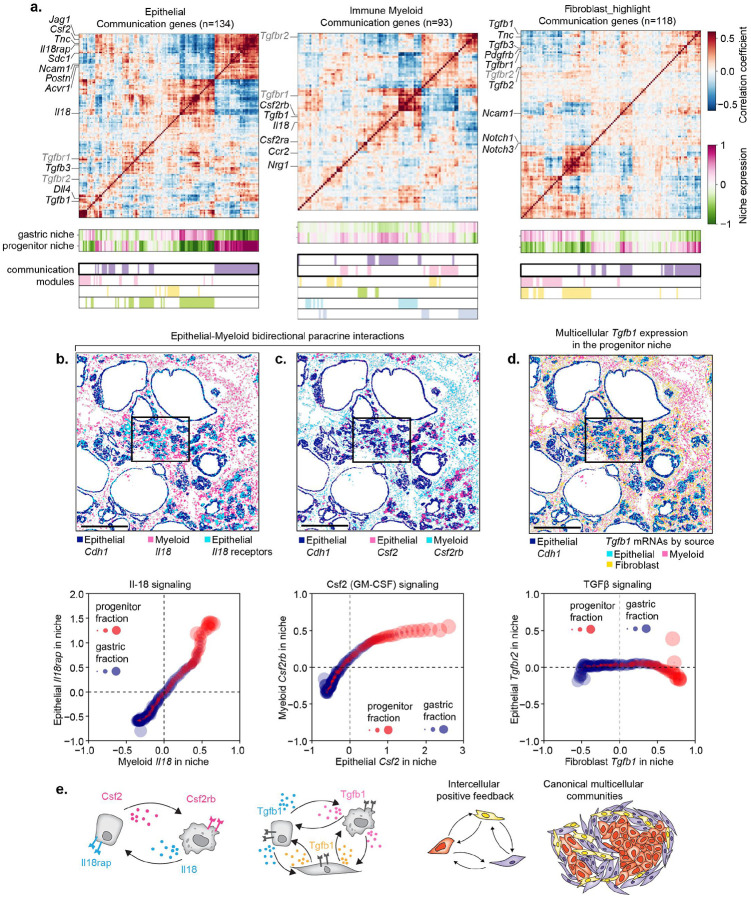
Intercellular communication modules define the progenitor niche. **a**. (Top) Gene-gene correlation matrix of communication gene niche expression in distinct cellular compartments. (Middle) Average expression of communication genes in canonical gastric of progenitor niches. (Bottom) Communication modules identified through graph-based community detection^120^. Boxes highlight communication modules associated with the progenitor niche in each compartment (see Methods). **b-d**. (Top) Each dot is a single mRNA detected in a specific cellular compartment. Scatter plots show colocalization of cognate ligand-receptor pairs from (b) IL-18, (c) GM-CSF signaling, or (d) *Tgfb1* produced in different cellular compartments (see Fig. S8c for markers of progenitor-like cells and associated niche cells in the same tissue region). (Bottom) Niche expression of cognate ligands and receptor pairs from (b) IL-18, (c) GM-CSF, or (d) TGFβ signaling. Each dot denotes the average niche gene expression of the specified communication genes in a specific bin along the gastric-progenitor niche continuum. **e**. Schematic of multicellular interaction circuits enabled by engagement of communication modules in the progenitor niche.

We built upon our previously developed method Calligraphy, a computational approach that leverages the modular organization of communication genes for the discovery and prioritization of cell-cell interactions in single cell data^37^. We quantified cell-type specific expression of communication genes at the niche level, followed by computation of gene-gene covariance matrices (see Methods), and found that communication genes exhibited compartment-specific modular organization in our spatial data (Fig. 5a and Fig. S8a), consistent with our prior work^37^. The added spatial dimension of our data allowed us to identify communication modules associated with the progenitor niche. Within each compartment, we found at least one communication module with enriched expression in the surroundings of progenitor-like cells, indicating that spatially coordinated signaling may contribute to the emergence of these multicellular communities.

Inspection of cognate ligand-receptor pairs between communication modules from distinct cellular compartments operating in the progenitor niche revealed signaling axes spanned multiple modalities, including juxtacrine signaling (e.g., epithelial *Jag1* – fibroblast *Notch3*); ECM production coupled to receptor upregulation (e.g., fibroblast *Postn* and *Tnc* – epithelial *Itgb3* and *Sdc1*); and paracrine interactions (e.g, epithelial *Pdgfb* – fibroblast *Pdgfrb*; myeloid *Nrg1* – epithelial *Itgb3*), potentially mediating MAPK activation in receiving cells^81^ (Fig. S8b). In contrast, ligand–receptor pairs expressed in spatially segregated compartments (e.g., epithelial *Lif* – fibroblast or myeloid *Lifr*) (Fig. S8b) lacked communication potential and may reflect emergent spatial patterning shaped by antagonistic interactions^82^.

To identify signaling interactions most likely to drive progenitor niche formation, we prioritized receptor-ligand pairs that not only co-localized within the niche but also increased in abundance along the gastric–progenitor axis. Such differentially engaged signaling axes are strong candidates for mediating state-specific changes underlying emergence of the progenitor niche. For example, we observed progressive and coordinated upregulation of cognate ligand–receptor pairs along the average gastric–progenitor DC axis, including myeloid *Il18* – epithelial *Il18rap* and epithelial *Csf2* – myeloid *Csf2rb* (Fig 5b,c). Importantly, spatial covariation is not strictly required to determine signaling potential: selective upregulation of a ligand or receptor in one compartment—paired with broad expression of its cognate partner—may also imply spatially constrained communication potential. For example, *Tgfb1* is selectively upregulated in epithelial, fibroblast, and myeloid cells of the progenitor niche (Fig. 5d), potentially activating TGFβ signaling within adjacent epithelial populations that ubiquitously expresses *Tgfbr1* and *Tgfbr2* (Fig. S8c)–a pattern consistent with the enrichment of TGFβ transcriptional signatures in progenitor-like cells (Fig. S3b).

Together, these findings support a model in which progenitor niches arise through a self-organizing circuit of spatially-constrained, reciprocal intercellular signaling. Oncogenic KRAS activation leads to the emergence of progenitor-like epithelial cells which express a distinctive set of signaling ligands and receptors that contribute to the communication potential of this premalignant subpopulation and its microenvironment. These stromal populations, in turn, engage in feedback signaling—via ligand production or extracellular matrix remodeling—that may stabilize and sustain the progenitor-like state, reinforcing the spatial and transcriptional architecture of the niche (Fig. 5e). The net result of these self-reinforcing circuits is the coordinated assembly of a cancer-like ecosystem that may promote cancer initiation.

### Oncogenic *Kras* inhibition dismantles the progenitor niche

The highly coordinated nature of progenitor niche assembly raised the possibility that perturbing a single key component could destabilize the entire system. Given that oncogenic KRAS drives expression of communication modules that define the progenitor state (Fig. S8d)^37^, we hypothesized that this population orchestrates niche formation by rewiring intercellular communication. Moreover, because progenitor-like cells exhibited heighted KRAS activity (see Fig. 2d), we hypothesized that the maintenance of this state and broader niche architecture might depend on persistent oncogenic KRAS signaling.

**Figure 6.**
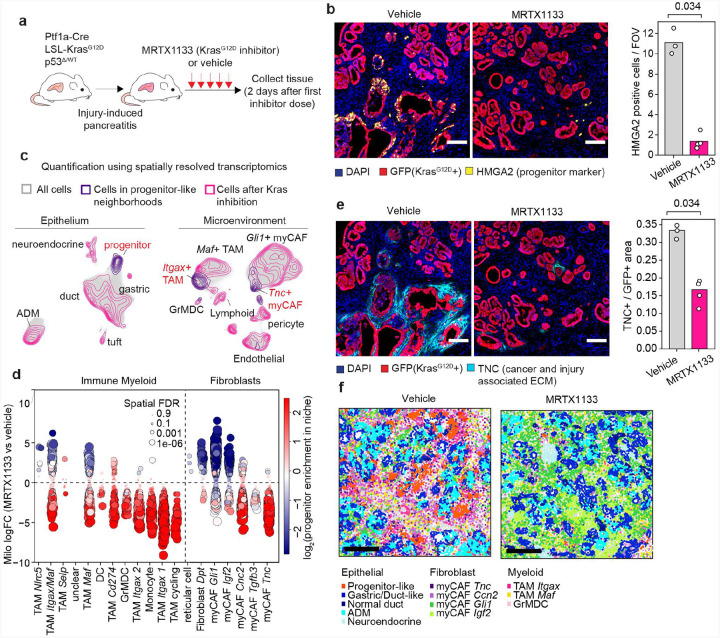
Consequences of acute oncogenic Kras inhibition in the premalignant pancreas. **a**. Timeline of acute oncogenic Kras inhibition in the premalignant pancreas. **b**. Representative images and quantification of HMGA2 staining in KP^LOH^ mice treated with vehicle (n = 3) or MRTX1133 (n = 4). Tissue was collected 2 days after the first inhibitor or vehicle dose. Scale bars, 50 μm. Each dot in quantification corresponds to an individual mouse; bar corresponds to average value. **c**. Two-dimensional density representation of Xenium single-cell gene expression data from mice treated with vehicle (n = 2) or MRTX1133 (n = 4). Purple contours, density of transcriptional states in the vicinity of progenitor-like epithelial cells; pink contours, density after MRTX1133 treatment. **d**. Differential abundance analysis of transcriptional neighborhoods of fibroblasts or myeloid cells in MRTX1133-treated compared to vehicle-treated samples. Each dot represents a transcriptional neighborhood as defined by MiloR^84^ (see Methods). Color represents the enrichment of progenitor-like cells in the spatial vicinity of cells in the transcriptional neighborhood. Significantly enriched or depleted transcriptional neighborhoods are outlined in black. **e**. Representative images and quantification of TNC staining in KP^LOH^ mice treated with vehicle (n = 3) or MRTX1133 (n = 4). Tissue was collected 2 days after the first inhibitor or vehicle dose. **f**. Representative images of Xenium data from vehicle or MRTX1133 treated mice. Each dot is a cell centroid, and colors represent select cell states. Scale bars, 250 μm. **b,e**. Scale bars, 50 μm. Each dot in quantification corresponds to an individual mouse; bar corresponds to average value. P-value, Two Tailed Wilcoxon Rank Sums test.

To test the cell state and tissue-wide consequences of removing this signal, we subjected treated KP^LOH^ mice to acute pancreatitis, followed by a 48 hour pulse of MRTX113^83^, a mutant KRAS^G12D^-specific small molecule inhibitor (Fig. 6a and Methods). This short-term perturbation enabled us to determine the immediate effects of perturbing KRAS signaling without directly targeting their microenvironment. Histological evaluation confirmed target engagement, as evidenced by the reduced phospho-ERK (p-ERK) levels in the epithelial compartment (Fig. S9a). Strikingly, this treatment triggered a rapid depletion of HMGA2+ progenitor-like cells without ablating the entire premalignant epithelium (Fig. 6b). Single cell transcriptional profiling coupled with differential abundance analysis using Milo^84^, revealed that progenitor-like cells were the most depleted upon acute oncogenic KRAS inhibition. In addition, gastric chief-like and pit-like cells– characterized by gene expression signatures of PanIN and the classical PDAC subtype^40^– were also depleted, albeit to a lesser extent (Fig. 6f, Fig. S9b,c). These results demonstrate that the maintenance of the progenitor-like state is exquisitely dependent on persistent oncogenic KRAS signaling.

Our scRNA-seq profiling revealed other informative changes. For example, ADM cells remained but showed transcriptional changes consistent with acinar recovery (Fig. S9d-,e), likely representing the initial phase of restoration of a normal pancreas histology that results from chronic oncogenic KRAS inhibition^16,85^. Moreover, we observed coordinated downregulation KRAS driven transcriptional programs, p53 target genes, and *Cdkn2a* expression (Fig. S9f), changes largely attributable to depletion of the progenitor compartment (Fig. 6b and Fig. S9g). Notably, rare residual progenitor-like cells retained high expression of these programs, suggesting incomplete KRAS inhibition or alternative mechanisms for sustaining tumor suppressive responses (Fig. S9g). Therefore, sustained oncogenic KRAS signaling is essential for maintaining progenitor-like epithelial states that also engage tumor suppressive responses in the injured pancreas.

To assess how depletion of the progenitor-like population impacts surrounding tissue, we applied the Xenium platform to MRTX1133-treated (n=4) and untreated (n=4) tissues collected 48h after the first inhibitor dose, analyzing a total of 2,686,667 cells. Spatial analysis revealed widespread shifts in cellular states across compartments, including the expected loss of progenitor-like cells (Fig. 6c and Fig. S10a) accompanied by striking depletions in *Tnc*^+^ myofibroblasts and *Itgax*^+^ macrophages/monocytes, otherwise enriched in the progenitor niche (Fig. 6e). To systematically quantify these changes, we adapted Milo^84^ to our spatial framework (Fig. S10b and Methods), assessing how progenitor-like cell depletion triggered by MTRX1133 treatment altered the abundance microenvironment state associated with the progenitor niche. This analysis confirmed that the microenvironment cells most tightly associated with progenitor-like cells – *Tnc+* myofibroblasts and *Itgax+* macrophages/monocytes – were preferentially depleted upon oncogenic KRAS inhibition (Fig. 6d and Fig. S10c,d), whereas populations excluded from progenitor niches, such as *Gli1+* myofibroblasts, became enriched (Fig. 6f and Fig. S10c). Although other premalignant subpopulations, including gastric pit and chief-like cells, were also affected (Fig. 6f and Fig. S9b,c), the predominant effect of MRTX1133 treatment was the collapse of the progenitor niche itself (Fig. 6d–f). These data imply that progenitor-like cells actively shape their environment and that targeted depletion of this state is sufficient to collapse the entire niche.

### *p53* naturally collapses the progenitor niche

**Figure 7.**
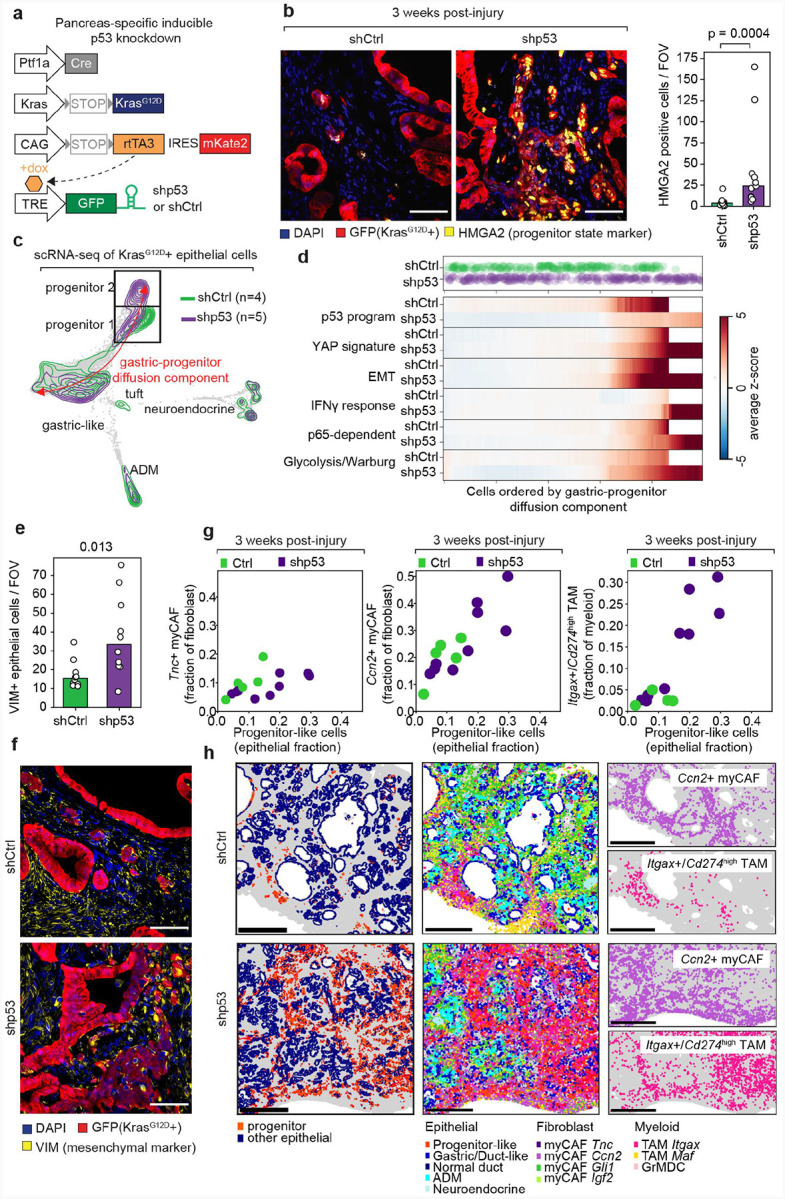
Consequences of *p53* knockdown in the premalignant pancreas. **a**. Mouse model for doxycycline-inducible knockdown of *p53* in the premalignant pancreatic epithelium. **b**. Representative images and quantification of HMGA2 staining 3 weeks post-pancreatitis in shp53 (n = 11) or shRen (n = 10) mice. **c**. Two-dimensional density representation of single-cell transcriptomes from shRen (n = 4) or shp53 (n = 5) *Kras*^G12D^+ pancreatic epithelial cells. **d**. Randomly sampled shp53 or shRen cells (top) and average score of expression signatures in shRen or shp53 cells (bottom) binned along the gastric–progenitor DC (bins <10 cells are not plotted). **e,f**. Representative images and quantification of VIM staining 3 weeks post-pancreatitis in shp53 (n = 10) or shRen (n = 10) mice. **g**. Xenium-based quantification of microenvironment subpopulations associated with the progenitor niche, as a function of the fraction of progenitor-like cells in the tissue. Each dot is a single biological replicate. **h**. Representative images of Xenium data from KC^shRen^ or KC^shp53^ mice 3 weeks post pancreatitis. Each dot is a cell centroid, and colors represent select cell states. Side panels show the abundance of *Ccn2*+ myofibroblasts or *Itgax+/Cd274*^high^ macrophage/monocytes in their associated sample. Scale bars, 500 μm. **b**,**e**. Scale bars, 50 μm. Each dot in quantification corresponds to an individual mouse; bar corresponds to average value. P-value, Two-tailed Wilcoxon Rank Sums test.

Although oncogenic KRAS inhibition rapidly dismantles the progenitor niche, this injury induced tissue state also resolves naturally if inflammation subsides (see Fig. 3b)^37^. Because progenitor-like cells activate tumor suppressive programs, including those governed by p53, we examined how p53 inactivation influences progenitor-like cell dynamics and niche architecture using a conditional mouse model that permits spatial and temporal control of endogenous p53 expression^54^ (Fig. 7a). Specifically, the KC^shp53^ model is derived from multiallelic ES cells and features Cre-dependent activation of oncogenic *Kras*^*G12D*^ and a mKate-coupled reverse tetracycline transactivator (rtTA), enabling epithelial-specific induction of tetracycline-responsive transgenes encoding either a GFP-linked p53-targeting shRNA (shp53) or a non-targeting shRNA control (targeting *renilla luciferase*, hereby referred to as shCtrl) upon doxycycline treatment. This system affords synchronous p53 knockdown in pre-tumor p53 proficient cells (mKate2+/GFP+) while avoiding confounders associated with chronic p53 inactivation in traditional KPC models^20^.

We first examined how p53 modulates progenitor-like cell dynamics and cell state transitions following injury. Following one week of doxycycline treatment to induce shRNA expression, KC^shp53^ and KC^shCtrl^ mice were treated with caerulein to induce pancreatitis and euthanized 3 weeks later for histological and molecular analysis. Reinforcing the notion that p53 selectively targets this subpopulation, p53 suppression produced a marked expansion of HMGA2+ progenitor-like cells compared to controls (Fig. 7b and Fig. S11a,b). Furthermore, beyond simply promoting progenitor-like cell persistence, scRNA-seq analysis revealed that p53 inactivation led to emergence of a distinct cell state displaying more mesenchymal features, such as increased expression of Vimentin (*Vim*) (Fig. 7c and Fig. S11a,b). We refer to the state present in both p53 wild-type and p53-suppressed tumors as progenitor 1, and the mesenchymal-like state arising specifically upon p53 loss as progenitor 2.

Differential gene expression analysis revealed that in addition to acquiring mesenchymal features, progenitor 2 states cells activated inflammatory responses and oncogenic programs, including interferon signaling, Yap signaling, and glycolysis (Fig. 7c–f, and Fig. S11e,f). Furthermore, diffusion component analysis revealed that these features of the progenitor 2 subpopulation emerged progressively along the gastric-progenitor axis as a continuation of the gastric-progenitor diffusion component (Fig. 7c,d). These results suggest that *p53* loss facilitates the progression of a molecular program that is initiated by oncogenic KRAS signaling in progenitor-like cells.

To disentangle direct effects of p53 loss from those driven by shifts in cell state composition, we performed a more focused analysis (Fig. S11c–e). Across *all* progenitor-like cells, p53 suppression broadly downregulated canonical p53 targets and epithelial identity genes (e.g., *Cdh1*, *Epcam*) (Fig. S11c); however, when restricting comparisons to clusters occupying comparable positions along the gastric–progenitor axis, only p53 target gene repression persisted (Fig. S11d and Methods). Therefore, p53 loss has two separable effects: it disables its canonical transcription program and licenses further plasticity that enables epithelial cells to adopt more mesenchymal cell states.

We next asked how p53 loss influences the composition of the progenitor niche using the Xenium platform (9 tissues from KC^shp53^ mice and 5 tissues from KC^shRen^ mice, 4,611,972 cells total). While stromal cell type abundances were similar globally between p53-proficient and deficient tissues three weeks post-injury (Fig. S12a), this masked substantial spatial heterogeneity (Fig. S12b,c). Knockdown of p53 caused progenitor niches to form expansive, contiguous lesions that spanned entire pancreatic lobes, a pattern that was absent from controls (Fig. S12c). These large tissue domains produced the most striking changes in microenvironment composition.

In the fibroblast compartment, *Tnc*+ activated myCAFs localized near progenitor-like cells regardless of p53 status (Fig. 7g,h), but their abundance did not scale with progenitor-like cell expansion (Fig. S12a). In contrast, myCAFs expressing the members of the matricellular CCN family *Ccn1* and *Ccn2* were increased in progenitor niches^74,86^. Their expansion upon p53 knockdown is consistent with their association with rare Vim+ progenitor-like cells in KC^shCtrl^ mice (Fig. S6 and Fig. S7c). Notably, p53-deficient progenitor-like cells upregulated multiple ECM components, as well as the mechanosensing gene *Piezo2* (Fig. S11e), suggesting that this premalignant subpopulation actively responds to, and potentially reshapes, the fibrotic microenvironment of the premalignant pancreas.

The most profound effects of p53 loss were in the myeloid compartment, where the expansion of progenitor-like cells was accompanied by the accumulation of *Itgax*^+^*/Cd274*^*high*^ (PD-L1^high^) macrophages (Fig. 7g,h). Single-cell and spatial transcriptomic analyses confirmed that these PD-L1^high^ cells constitute a distinct cell state (Fig. S12d,e), whose abundance increased sharply once progenitor-like cells exceeded a threshold and then scaled proportionally with niche size (Fig. S12f). Single-cell profiling revealed that, compared to their *Cd274*^low^ counterparts, *Itgax*^+^/*Cd274*^*high*^ monocytes/macrophages upregulated immunosuppressive genes (e.g., *Arg1, Spp1, Marco*)^62,64,87^, as well as markers of the alternative macrophage activation state (e.g., *Chil3, Ms4a8a*)^88,89^, and downregulated MHC class II components (e.g., *H2*-*Eb1, H2*-*Aa, Cd74*) (Fig. S12g). These observations suggest that p53-deficient progenitor cells drive the expansion and evolution of an immune-privileged niche.

Taken together, these findings demonstrate that either KRAS inactivation or p53 engagement is sufficient to dismantle the progenitor-like population and its associated niche. While oncogenic KRAS activity promotes epithelial–mesenchymal plasticity and microenvironmental remodeling—hallmarks of the regenerative phase of wound healing—p53 activation restrains these processes, collapsing the progenitor niche and mimicking the resolution of tissue repair. Beyond its well-established role in restraining genomic instability^27,90,91^, these results position p53 as a critical barrier to both cell-intrinsic and microenvironmental plasticity at a key inflection point between benign and malignant states.

## Discussion

By directly visualizing the cellular and molecular events that promote or restrain the benign-to-malignant transition, our study identifies a progenitor-like cell state as the pivotal target of oncogenic and tumor-suppressive forces during early pancreatic tumorigenesis. We show that this transiently emergent population—induced by oncogenic *Kras* in the context of tissue injury—engages tumor suppressive pathways governed by p53, CDKN2A, and SMAD4, triggering senescent-like programs and activating intercellular communication programs that remodel the surrounding environment. Likely through reciprocal signaling across epithelial, fibroblast, and immune compartments, progenitor-like cells assemble a multicellular niche with hallmarks of invasive cancer. Malignant progression ensues when this progenitor-like state escapes tumor-suppressive surveillance, enabling immune evasion, persistent epithelial plasticity, and stromal co-option. These findings position the progenitor-like state as a gatekeeper of malignant transformation and define a discrete window during which targeting the signaling circuits and cell states that support niche assembly may allow for effective cancer interception.

This work was enabled by methodological innovations that directly visualize early malignant progression in situ. Genetically engineered models that mark spontaneous *p53* loss captured sporadic tumor initiation^27,92,93^, while high-resolution spatial transcriptomics technologies^94^ reconstructed the multicellular ecosystems surrounding progenitor-like cells. Anchoring spatial analysis on transitional epithelial states and mapping continuous gene expression trajectories revealed coordinated shifts in epithelial plasticity, niche remodeling, and tissue architecture along a path to malignancy. Transcriptional gradients in premalignant cells guided the dissection of dynamic and progressive remodeling events that assemble the progenitor niche – a cancer-like environment characterized by immunosuppression and activated wound healing programs. These analyses, analogous to pseudotime construction in dissociated data^56,69,95–99^, provide an inferred timeline of niche-state transitions and establish a generalizable framework for investigating how cell state transitions orchestrate tissue-scale organization in regeneration, fibrosis, and early tumor evolution.

Using these methods, we pinpoint when, where, and how key oncogenic and tumor-suppressive forces act during malignant progression. Surprisingly, the major tumor suppressor pathways implicated in PDAC—p53, CDKN2A, and SMAD4—are not broadly engaged across the premalignant epithelium, but instead converge on a discrete progenitor-like population that displays high KRAS signaling and is transcriptionally poised for transformation. Single-cell analyses of other premalignant tissues^40,47,100–102^ have identified senescent-like/transitory cells resembling the progenitor populations described herein, suggesting that targeting progenitor-like programs may be a conserved tumor suppressive mechanism. Intriguingly, the selective engagement of tumor suppressors in the highly plastic progenitor-like state echoes the long-standing finding that p53 and p16^INK4A^ (encoded in the CDKN2A locus) suppress induced pluripotency^103–107^—the ultimate example of cellular plasticity. Viewed through this lens, our study provides direct visual evidence that p53’s tumor suppressive function stems from its role as a guardian of plasticity, facilitating the resolution of regenerative or progenitor-like states that, if unchecked, promote maladaptive remodeling and tumorigenesis. Our data suggest that the decision between benign persistence and malignant progression occurs within the narrow window defined by the emergence, activation, and resolution of the progenitor-like state.

We further revealed the potential for bidirectional communication between the premalignant epithelial cells and their microenvironment within the progenitor niche. This signaling appears enabled by concerted upregulation of communication modules across compartments–TGFβ signaling, ECM/ECM receptor communication, and immune cytokine- mediated heterotypic crosstalk. The modular and reciprocal nature of these interactions suggest that positive feedback loops stabilize the progenitor niche, mirroring systems-level architectures that mediate regenerative and developmental transitions^108^. Supporting this model, acute KRAS inhibition not only depletes progenitor-like cells but simultaneously dismantles their niche, leading to rapid loss of immunosuppressive macrophages and activated myofibroblasts. These findings reveal that malignant progression is not driven by epithelial transformation alone, but by the emergent properties of a multicellular ecosystem.

These insights converge on a broader principle: the malignant potential of progenitor-like cells is shaped by the interplay between genetic lesions, epithelial plasticity, and microenvironmental remodeling. Prior studies have shown that oncogenic KRAS derails normal regenerative programs, driving chromatin remodeling that induces a highly plastic progenitor state with heightened cell–cell communication potential^37^—features that mimic physiological wound healing but become pathologically sustained in cancer^109^. In this context, the progenitor-like state functions as both a target of tumor suppressive engagement and a hub where persistent KRAS signaling impedes wound resolution, such that targeting oncogenic KRAS activity or engaging p53 transcriptional programs allows resolution to proceed. These results align with emerging evidence that p53 restrains exaggerated injury responses in other epithelial tissues^100,110,111^ and support a model in which p53 and KRAS co-modulate both cell-intrinsic programs and tissue-scale dynamics to govern cancer risk.

This study reveals that oncogenic KRAS inhibition and p53 activation converge on depleting a progenitor-like state that would otherwise seed a tumor-permissive niche, marking a decisive point in malignant progression. While p53 restricts this state, its loss enables persistence, progression towards advanced mesenchymal states, and immune evasion, rendering the niche susceptible to transformation. These findings help contextualize the activity of KRAS inhibitors in advanced PDAC, where they preferentially eliminate basal/mesenchymal-like populations and remodel the tumor microenvironment, in some instances enhancing responsiveness to immunotherapy^112–115^. Our data suggest that these effects reflect, in part, collapse of the progenitor-like state and restoration of regenerative resolution, thereby permitting immune surveillance. Given the strong association between *TP53* mutations and aggressive, treatment-refractory cancers, targeting *p53*-mutant tumors has long been a central goal in oncology. Our data argue that interception efforts should target not only initiating mutations but also the specific cell states and multicellular ecosystems in which p53 operates. By revealing how p53 naturally suppresses malignant progression, our findings suggest that eliminating the progenitor-like states it constrains may represent a tractable and broadly applicable therapeutic strategy—one that phenocopies p53 function without requiring its restoration.

## METHODS

### I. Experimental Methods

#### MOUSE MODELS

All animal experiments were performed in accordance with protocols approved by the Memorial Sloan Kettering Institutional Animal Care and Use Committee (approval number: 11–06-018). Mice were maintained under specific pathogen-free conditions, and provided with food and water *ad libitum*. In all experiments with PDAC models, tumors did not exceed a volume corresponding to 10% of body weight (typically 12–15-mm diameter). Mice were evaluated daily for signs of distress or end-point criteria, and immediately euthanized if they presented signs of cachexia, weight loss beyond 20% of initial weight or breathing difficulties, or if they developed tumors of 15 mm in diameter. Animals were housed on a 12 h light–12 h dark cycle under standard temperature (18–24 °C) and humidity (40–60%).

##### Mouse model genetics

The KP^LOH^ model^1,2^ allows the identification and isolation of cells that undergo spontaneous *p53* loss of heterozygosity (LOH) during pancreatic cancer initiation. This model is derived from multi-allelic ES cells and harbors *Ptf1a*-*Cre*; *LSL*-*Kras*^*G12D*^; *p53*^*flox/WT*^ alleles that predispose mice for spontaneous cancer development. Embryonic expression of *Ptf1a*-Cre in pancreatic epithelial progenitor cells leads to Cre-dependent excision of the LSL (lox-STOP-lox) cassette upstream of mutant *Kras*^*G12D*^, and deletion of one copy of *p53*. Oncogenic KRAS activity in the pancreatic epithelium leads to the formation of premalignant lesions^3^ and eventual PDAC development^1,2^ upon loss of the remaining wild-type copy of *p53* in premalignant cells (*p53*-LOH).

The KP^LOH^ model harbors fluorescent proteins that trace the lineage of cells that experienced Cre activity, as well as cells that have undergone *p53*-LOH. This model includes the *Rosa26*-*CAGGS*-*LSL*-*rtta*-*IRES*-*mKate2 (RIK)* allele^4^, in which Cre-dependent excision of the LSL cassette leads to constitutive polycistronic production of the reverse tetracycline transcriptional activator (rtTA3) and mKate2. Both proteins serve as proxies for oncogenic *Kras*^*G12D*^ activation in epithelial cells (detected through mKate2 immunofluorescence), single molecule fluorescence in situ hybridization (smFISH) or transcriptomics. Furthermore, upon doxycycline administration, rtTA3 expression allows selective induction of transgenes downstream of a promoter harboring the tetracycline-regulated element. This genetic configuration thus allows both detection and perturbation of premalignant cells *in vivo*.

Lastly, the KP^LOH^ model harbors the doxycycline-inducible *TRE*-*GFP*-*shRen.713* allele, which produces GFP and a short-hairpin RNA (shRNA) targeting *Renilla* luciferase (shRen) upon doxycycline administration. We have used this allele extensively as a non-targeting negative control^5^ to account for non-specific effects of shRNA-expression and interaction with the RNAi machinery in a cell. In the context of the KP^LOH^ model, this allele serves the unique purpose of reporting for the genetic status of *p53 in vivo*. The TRE-GFP-shRen allele is located in the *Col1a1* safe-harbor locus in mouse chromosome 11, in cis with the single *p53*^WT^ allele (due to how we designed our breeding scheme). Given the selective pressure for homozygous *p53* loss during PDAC development, and the fact that *p53*-LOH events most frequently occur through whole chromosome or large segmental deletion events^1^, loss of GFP serves as a proxy for *p53*-LOH in this model.

All cohorts in this study were derived from multiallelic mESCs harboring the genetic configuration described above (KPfCRC shRen clone YMZ). While this model uniquely allows tracing and isolation of cells that underwent spontaneous *p53*-LOH events *in vivo*, we have also used these mESCs to study *p53*-proficient cells in the premalignant pancreas due to their high efficiency in generating experimental mice.

KC^shRNA^ models harbor the same genetic configuration of the KP^LOH^ model, with the exception that they have *p53*^WT/WT^ alleles. We use two variants of this model: KC^shCtrl^ harbors the *TRE*-*GFP*-*shRen.713* allele, serving as a non-targeting negative control (shRen), and KC^shp53^ harbors the *TRE*-*GFP*-*shp53.1224* allele^5^ targeting shp53. We used these models to investigate the consequences of inducible p53 knockdown *in vivo*. All KC^shRNA^ mice in this study were generated using multiallelic mESCs (KC^shCtrl^: clone p48–9c shRen.713 c2; KC^shp53^: clone p48–9c shp53.1224 #6)^6^.

The identity of the ESCs and ESC-derived mice were authenticated by genomic PCR using a common Col1a1 primer paired with an shRNA-specific primer:

- Col1a1: 5’-CACCCTGAAAACTTTGCCCC-3’
- shRen.713: 5’-GTATAGATAAGCATTATAATTCCTA-3’ (~250 bp band)
- shp53.1224: 5’-TGTATTACACATGTACTTGTAGTGG-3’ (~210 bp band)

The presence of the RIK allele in mESCs was confirmed using PCR with the following primers:

- 5’-GGTGAGCGAGCTGATTAAGG-3’
- 5’-TTTTGCTGCCGTACATGAAG-3’ (~200 bp band)

In addition, we confirmed shp53 and shRen expression at the single-cell level by aligning reads to the unique sequences that distinguish TRE-GFP-shp53 and TRE-GFP-shRen alleles:

>TGM_shRen_unique
TGCTGTTGACAGTGAGCGCAGGAATTATAATGCTTATCTATAGTGAAG
CCACAGATGTATAGATAAGCATTATAATTCCTATGCCTACTGCCTCGG
>TGM_shp53_unique
TGCTGTTGACAGTGAGCGCCCACTACAAGTACATGTGTAATAGTGAAG
CCACAGATGTATTACACATGTACTTGTAGTGGATGCCTACTGCCTCGG

##### Cohort generation

ESC-derived chimeric male mice were generated by injecting KP^LOH^, KC^shCtrl^ or KC^shp53^ backgrounds at the 8-cell or blastocyst stage, as previously described^5^, enabling the synchronous creation of large cohorts of mice bearing all alleles for modeling PDAC initiation and progression. Cohorts were generated by the Mouse Genetics Core Facility at Memorial Sloan Kettering Cancer Center (MSK) or the Rodent Genetic Engineering Core at New York University. Only mice with coat-color chimaerism of over 95% were included for experiments.

##### shRNA induction

To induce shRNA expression, KC^shCtrl^ and KC^shp53^ mice were switched to a doxycycline diet (200 mg kg^−1^, Harlan Teklad) at 4–5 weeks of age, one week before inducing pancreatitis. KP^LOH^ mice were switched to doxycycline diet at 4–5 weeks of age to allow doxycycline dependent induction of the GFP transgene that reports the presence or absence of the wild-type *p53* allele in this model.

For KC^shp53^ collection cohort 4 (Fig. S11a), a subset of mice were switched to doxycycline diet and the remaining animals were fed normal chow (used in Fig. 7b,e). *p53* knockdown led to an increase in progenitor-like cells and expansion of their niche in this comparison. These results not only corroborate previous findings, but also provide an orthogonal negative control, as they show that differences in the abundance of progenitor-like cells track with *p53* knockdown, as opposed to the specific mESC strain used to generate cohorts.

KP^LOH^ mice from batches 2, 7 and 8 (see Table S1 for details on sample metadata) were administered 625 mg kg^−1^ doxycycline diet (Harlan Teklad) following prior practices in our lab^1^. The remaining cohorts were treated with low dose doxycycline (200 mg kg^−1^) to minimize the potential effects of antibiotic treatment on the microbiome. No differences in the spectrum or dynamics of premalignant states were observed as a function of doxycycline dose.

#### TREATMENTS

##### Injury-induced pancreatitis

To assess the spectrum and dynamics of premalignant states and tissue remodeling events in the in the context of oncogenic KRAS activation in the premalignant epithelium, we subjected 5–6-week-old male mice with eight-hourly intraperitoneal injections of 80 μg kg^−1^ of caerulein (Bachem) for two consecutive days (16 injections total), as previously described^7^. Caerulein dose was adjusted to body weight at the beginning of each day of treatment. We harvested the pancreata at two phases of the injury response: an acute phase, corresponding to the peak of inflammation (days 1 and 2 after the 9th caerulein injection), or a long-term response (3 weeks post-caerulein treatment).

##### Oncogenic KRAS inhibition

The KRAS^G12D^-specific small molecule inhibitor MRTX1133^8^ allowed us to interrogate the consequences of acute removal of Kras signaling in premalignant cells without directly affecting the tumor microenvironment (Fig. 6, Fig. S9 and Fig. S10).

###### Formulation for *in vivo* use

We formulated MRTX1133 for *in vivo* use, as previously described^9^. To prepare the vehicle for drug administration, we first dissolved Captisol (MedChemExpress, HY-17031) at a 20% w/v concentration in sterile water. Next, we mixed the 20% Captisol solution with 100 mM citrate buffer pH 5.0 (Teknova, Q2443) in a 1:1 ratio, resulting in a final vehicle solution of 10% Captisol, 50 mM citrate buffer pH 5.0. To prepare the stock solution of MRTX1133, we diluted MRTX1133 powder in the vehicle solution to a final concentration of 3 mg mL^−1^. We stored vehicle and MRTX1133 formulations at 4°C protected from light for up to 1 week.

###### Dosing

We fed mice with MRTX1133 at the maximum tolerated dose of 30 mg kg^−1^ through intraperitoneal injection (200 μL for a 20-g mouse). Controls were dosed with an equivalent volume of vehicle based on weight. Dosing was twice a day, with an inter-dose interval of 10–12 h. We randomized MRTX1133 and vehicle-treated mice to control for differences in the social structure of individual cages, as well as inter-cage heterogeneity in average mouse size.

###### Experimental design

We used 5-week-old male KP^LOH^ mice generated through mESC injections as experimental cohorts. For mice profiled with scRNA-seq, we started dosing of MRTX1133 or vehicle concurrently with caerulein treatment, euthanizing mice within 4 h of the last MRTX1133 or vehicle dose (fifth dose, 2 days after the first dose). For mice profiled with the Xenium spatial transcriptomics, we started MRTX1133 or vehicle dosing 2 days after the first caerulein dose, euthanizing mice 2 days after the first dose of MRTX1133 or vehicle. These two experimental protocols aimed to assay the role of oncogenic KRAS signaling in inducing or maintaining the progenitor-like state upon acute pancreatitis. In practice, both protocols had equivalent outcomes in terms of the spectrum of premalignant lesions at end-point: depletion of progenitor-like, gastric pit-like and gastric chief-like cells, as well as shifts in the state of acinar-to-ductal metaplasia (ADM) cells, with progenitor-like cells being the subpopulation with strongest dependency on persistent oncogenic KRAS (Fig. 6c and Fig. S9c).

#### SAMPLE COLLECTION

Table S1 provides details of scRNA-seq samples collected for this study.

##### Experimental endpoints

Pre-tumor stage KP^LOH^ mice were euthanized at 3–4 months of age. Lack of a macroscopic tumor mass was assessed by palpation before euthanasia, and confirmed by gross histology upon dissection. Tumor stage KP^LOH^ mice were euthanized upon confirmation of the presence of a macroscopic tumor mass by palpation; two animals at 3 months of age, and one at 8 months. Mice subjected to acute pancreatitis in time course and *p53* perturbation cohorts were euthanized at 1 day, 2 days or 3 weeks after the second day of the pancreatitis protocol. Mice treated with MRTX1133 or vehicle were euthanized 2 days after the first treatment dose.

##### Tissue dissociation for single-cell analyses

For scRNA-seq and bulk RNA-seq collection, we isolated lineage-traced (mKate2+/GFP+ or mKate2/GFP-) epithelial cells from pancreatic tissues from KP^LOH^, or KC^shRNA^ mice by FACS sorting, as previously described^7,10^. Specifically:

1. Pancreata were finely chopped with scissors and incubated in digestion buffer containing 1 mg mL^−1^ collagenase V (Sigma-Aldrich, C9263), 2 U mL^−1^ Dispase (Life Technologies, 17105041) dissolved in HBSS with Mg^2+^ and Ca^2+^ (Thermo Fisher Scientific, 14025076) supplemented with 0.1 mg mL^−1^ DNase I (Sigma, DN25–100MG) and 0.1 mg mL^−1^ soybean trypsin inhibitor (STI) (Sigma, T9003), in gentleMACS C Tubes (Miltenyi Biotec) for 42 min at 37°C using the gentleMACS Octo Dissociator.
2. Digested samples were washed with PBS and further digested with a 0.05% solution of Trypsin-EDTA (Thermo Fisher Scientific, 15400054) diluted in PBS for 5 min at 37 °C. Trypsin digestion was neutralized with FACS buffer (10 mM EGTA and 2% FBS in PBS) containing DNase I and STI.
3. Samples were washed in FACS buffer containing DNAse and STI, and filtered through a 100-μm strainer.
4. Samples were blocked with anti-mouse CD16/CD32 with Fcblock (BD Biosciences, Cat# 553141; Clone 2.4G2) for 7 min at 4°C, followed by incubation with APC-conjugated CD45 antibody (Biolegend, Cat# 103111; Clone 30-F11, 1:200, 10-min incubation).
5. Cells were washed once in FACS buffer containing DNase I and STI, filtered through a 40-μm strainer and resuspended in FACS buffer containing DNase I and STI and 300 nM DAPI as a live-cell marker. Cells were sorted on BD FACSAria I or BD FACSAria III (Becton Dickinson) for mKate2+/GFP+ (Kras^G12D^+ *p53*-proficient epithelial cells) or mKate2+/GFP− (Kras^G12D^+ *p53*-deficient epithelial cells), excluding DAPI+ and CD45+ cells. FACS-sorted cells were collected in 2% FBS in PBS (see Fig. S1c for a representative example of FACS gates for isolating epithelial cells from a pre-tumor stage KP^LOH^ mouse).
6. For scRNA-seq, cells were resuspended at 1000 cells μL^−1^ in 0.04% BSA 1X PBS solution with RNAse inhibitor (Thermo Fisher Scientific, AM2684; 1 U μL^−1^ or 1:40 dilution from stock). In the case of pre-tumor *p53*-deficient cell isolation, the low frequency of this subpopulation (200–2000 cells per mouse) limited our ability to resuspend and process sorted cells directly from the sorter. To allow downstream processing of this rare and important cell population, we spiked-in CD45+ cells isolated from the same mouse to reach a threshold of 30,000 cells, resuspending in a final volume of 30 μL for downstream processing.

For *p53* perturbation and acute oncogenic KRAS inhibition experiments, we modified this isolation protocol to accommodate pooling of biological replicates in the same encapsulation and sequencing runs via cell hashing, minimizing both costs and batch effects. Following from step 2 above:

3. For every sample that would be subjected to cell hashing, we used DNAse-free buffers from this point on, reasoning that the presence of DNAse could hamper our ability to recover DNA barcodes.
4. Samples were resuspended in 1 mL ACK-lysis buffer and incubated for 5 min at room temperature to deplete red blood cells, and washed the ACK lysis buffer with 20 mL HBSS.
5. We blocked samples with TruStain Fc block Plus (Biolegend, 156603; clone S17011E, 1:100) for 7 min at 4°C, followed by incubation with a sample-specific TotalSeq cell hashing antibody (Biolegend, 155832, 155833, 155835, 155837, 155839, 155841; clones M1/42; 30-F11). We incubated cell hashing antibodies at a 1:50 dilution for 30 min.
6. We washed samples 3 times with DNAse-free FACS buffer + STI, followed by filtering through a 40-μm strainer and FACS-based isolation of mKate2+/GFP+ cells (Kras^G12D^+ pancreatic epithelial cells expressing shp53 or shCtrl).
7. To prepare cells for scRNA-seq, we pooled samples from the same experimental conditions into the same tube, and resuspended cells at a 1000 cells μL^−1^. This strategy ensured our ability to interpret differences between conditions even if deconvolution of biological replicates failed. In practice, we didn’t experience problems with downstream deconvolution of biological replicates in these data.

##### Preparation of tissues for histology

For immunofluorescence and Xenium-based analyses, tissues were fixed overnight in 10% neutral buffered formalin (Richard-Allan Scientific), and embedded in paraffin. Formalin fixed paraffin embedded (FFPE) blocks were stored at room temperature, or more recently at 4°C as we started profiling RNA from these tissues using spatial transcriptomic technologies.

For smFISH-based analyses, we followed the fixation protocol by Farack and Itzkovitz^11^. Specifically, we fixed tissue in 4% PFA (Fisher / Electron Microscopy Sciences, 15710) 1X PBS at 4°C for 3 h, followed by overnight incubation in 4% PFA, 1X PBS, 30% w/v sucrose solution, verifying that tissues sank to the bottom of the tube before further processing. We washed tissues with 1X PBS and thoroughly dried them with a kimwipe before OCT embedding. We incubated tissues for 30 min to 1 h incubation in OCT at 4°C, since we observed that this decreases the likelihood of tissue detachment during sectioning as compared to immediate freezing. Lastly, we completed embedding by placing tissues in a mold, fully covering with OCT, and placing on dry ice for freezing. We stored frozen OCT blocks at −80°C.

#### SINGLE-CELL RNA SEQUENCING

##### Fresh dissociated samples

Cells were resuspended in 1X PBS and BSA (0.04%) and checked for viability using 0.2% (w/v) Trypan Blue staining (Countess II). All sequencing experiments were performed on samples with a minimum of 80% viable cells. Single-cell encapsulation and scRNA-seq library prep of FACS-sorted cell suspensions was performed on the Chromium instrument (10x Genomics) following the user manual (Reagent Kit 3’ v2 or v3). Each sample loaded onto the cartridge contained approximately 5,000 cells (non-hashed samples) or 15,000 cells (hashed samples) at a final dilution of ~500 cells μl^−1^. Transcriptomes of encapsulated cells were barcoded during reverse transcription and the resulting cDNA was purified with DynaBeads, followed by amplification per the user manual. Next, the PCR-amplified product was fragmented, A-tailed, purified with 1.2X SPRI beads, ligated to sequencing adapters and indexed by PCR. Indexed DNA libraries were double-size purified (0.6–0.8X) with SPRI beads and sequenced on an Illumina sequencer (R1 – 26 cycles, i7 – 8 cycles, R2 – 70 cycles or higher) to a depth of >50 million reads per sample (>13,000 reads per cell) at MSK’s Integrated Genomics Operation Core Facility.

##### Dissociated nuclei from FFPE samples

FFPE samples were preprocessed using a prototype Singulator^™^ system. Each sample was automatically processed in a NIC+^™^ cartridge (S2 Genomics, 100–215-389) through two 10-min deparaffinization steps using Deparaffinization Reagent (S2 Genomics), followed by rehydration through successive 1 mL ethanol washes (100%, 100%, 70%, 50%, and 30%). This was followed by two PBS washes. The sample was then centrifuged at 1,000 *g* for 3 min and resuspended in 0.5 mL of Nuclei Isolation Reagent (NIR, S2 Genomics, 100–063-396) containing 0.1 U μL^−1^ RNase inhibitor (Protector^™^, Millipore Sigma, 3335399001). All subsequent solutions contained RNase inhibitor at the same concentration. The sample was dissociated into single nuclei in a second NIC+ cartridge using “FFPE Nuclei Isolation” protocol, using 0.5 mL of NIR for 12 min of lysis, followed by a 2-mL wash with Nuclei Storage Reagent (NSR, S2 Genomics, #100–063-405). The single-nucleus suspension was centrifuged at 500 *g* for 5 min, resuspended in NSR, and counted using 0.2% (w/v) Trypan Blue staining on a Countess II instrument. This was followed by a second centrifugation at 850 *g* for 5 min.

Nuclei were then resuspended in 1 mL of Fixation Buffer (4% formaldehyde in 1× Fix & Perm Buffer, 10x Genomics, PN-2000517) and incubated at 4°C for 16–24 h. To stop the fixation, nuclei were centrifuged at 850 *g* for 5 min at room temperature and quenched with 1 mL of Quenching Buffer (1× Quench Buffer, 10x Genomics, PN-2000516). Fixed nuclei were then stained with 1 μg mL^−1^ DAPI and sorted for DAPI-positive nuclei.

Up to 300,000 nuclei were processed per hybridization according to 10x Genomics recommendations. Each hybridization was performed in 40 μL of hybridization mix, containing 10 μL of Mouse WTA probes (10x Genomics, PN-2001275) and 2.5 μL of custom probes targeting eGFP and mKate2 for a final concentration of 2 nM per probe. Custom probes were designed following the 10x Genomics technical note on probe design, with particular attention to GC content (see Data S1 for probe sequences). Hybridizations were carried out at 42°C for 16–24 h.

Following hybridization, samples were diluted in Post-Hybridization Wash Buffer and counted. For each experiment, an equal number of nuclei from each hybridization reaction was pooled to ensure equal sample representation. The pooled nuclei were then washed four times in Post-Hybridization Wash Buffer for 10 min at 42°C. After washing, nuclei were resuspended in Post-Hybridization Resuspension Buffer, filtered through a 30 μm Miltenyi Biotec filter, and counted to determine the appropriate volume for loading onto the Chromium X instrument.

GEM encapsulation was performed following the 10x Genomics Flex GEM-X (PN-1000782) protocol, using their guidelines for cell and reagent volumes per well based on the desired cell recovery. After loading the Chip FX and running it on the Chromium X, GEMs were recovered and processed according to the manufacturer’s instructions. Following GEM processing, the resulting product was pre-amplified and indexed to generate the sequencing library. All libraries were sequenced on an Illumina NovaSeq X+ (R1 – 28 cycles, i5 – 10 cycles, i7 – 10 cycles, R2 – 90 cycles) using standard dual indexing and demultiplexing. Raw BCL files were processed with Cell Ranger (9.0.0), and the resulting FASTQ files were quantified using a custom probe set reference for the mouse genome (GRCm39) within the Cell Ranger pipeline.

Table S1 provides details of the dissociated samples collected as part of this study.

#### SPATIAL PROFILING

Table S5 describes detailed information regarding the 10x Xenium spatial transcriptomics samples that we collected as part of this study. Data S4 contains source data and sample metadata of tissues analyzed with immunofluorescence.

##### Immunofluorescence

Immunofluorescence was conducted on 5-μm sections of FFPE blocks. Following deparaffinization and antigen retrieval (citrate buffer pH 6.0, Fisher / Vector Biolabs, H-3300–250), slides were blocked with 5% BSA 1X PBS for 1 h at room temperature, followed by overnight incubation with primary antibodies. Following primary incubation, we washed slides 3 times for 10 min with 1X PBS, and incubated with secondary antibodies diluted in blocking buffer for 1h. We washed slides for 10 min with 1X PBS 1 ug mL^−1^ DAPI, followed by two additional washes and mounting. Images were imaged with a Nikon T2i Eclipse system equipped with a 20X Plan APO objective (Nikon, MRD00205) equipped and an ORCA-FusionBT sCMOS camera. For quantification of HMGA2, VIM and TNC, we collected full tissue scans using the Nikon Elements image acquisition software.

For quantification of P53 levels, we stained adjacent tissue sections for the progenitor state marker MSN or P53, as well as GFP as a proxy for *Kras*^G12D^+/*p53*-proficient cells. We selected and acquired fields of view based on the presence of MSN+ and MSN− lesions and subsequently acquired the corresponding regions of interest in the p53-stained slide. We acquired images using a 20X Plan APO objective and the Crest X-Light V2 LFOV25 Spinning Disk Confocal attached to our Nikon T2i Eclipse microscope, collecting fields of view of 2000 × 2000 px.

We used the following primary antibodies: GFP (Abcam, ab13970; RRID:AB_300798, 1:1000), RFP (Evrogen, AB233; RRID:AB_2571743, 1:1000), HMGA2 (Cell Signaling Technology, 8179S; clone D1A7, RRID:AB_11178942, 1:500), Moesin (Proteintech, 26053–1-AP; RRID:AB_2880353, 1:100), E-cadherin (BD Biosciences, 610181; RRID:AB_397580, 1:500), Vimentin (Cell Signaling Technology 5741; RRID:AB_10695459, 1:500), mKate2 (generated in-house, #4007; rat isotype, 1:250), p19 (SantaCruz, sc-32748; RRID:AB_628071 1:100), Tenascin-C (R&D systems, MAB2138; RRID:AB_2203818, 1:250), p53 (Leica Biosystems, P53-CM5P; RRID:AB_2744683, 1:250), Phospho-p44/42 MAPK (Erk1/2) (Thr202/Tyr204) (Cell Signaling Technology, 9101; 1:250). We used the following secondary antibodies as part of this study: Donkey Anti-Mouse IgG Alexa Fluor 750 (Abcam, ab175738; 1:250), Donkey anti Rabbit IgG Alexa Fluor 555 (Invitrogen, A31572; 1:500), Donkey anti-Rat IgG Antibody, Alexa Fluor 647 (Thermo Fisher Scientific, A78947; 1:500), Donkey anti-Chicken IgY Alexa Fluor 488 (Thermo Fisher Scientific, A78948; 1:1000), Donkey Anti-Chicken IgY Alexa Fluor 647 (Sigma-Aldrich, AP194SA6; 1:1000), Donkey anti Mouse IgG Alexa Fluor 488 (Thermo Fisher Scientific, A21202; 1:1000), Donkey anti-Rabbit IgG Alexa Fluor Plus 647 (Invitrogen; A32795, 1:500), Donkey anti-Rat IgG Alexa Fluor Plus 555 (Invitrogen, A48270; 1:500).

##### Multiplexed Immunofluorescence using Lunaphore COMET

The Lunaphore COMET platform allowed us to probe for multiple markers of the progenitor-like state in the same tissue slide, overcoming limitations of isotype incompatibility between markers (e.g., MSN and HMGA2 antibodies are both derived from rabbit hosts). Tissue sections (5 μm) were trimmed from a FFPE block and placed at the center of a clean glass slide. The slide was air dried and baked at 42°C for 3 h and stored in a desiccator. Epredia PT Module was used to deparaffinize and retrieve epitopes (Epredia Dewax and HIER Buffer L). The slide was then washed twice with 1X Multistaining buffer (BU06) and loaded onto the COMET. Appropriate volumes of primary antibodies, secondary antibodies, 5 μg mL^−1^ DAPI (Thermo Fisher Scientific, D3571), Multistaining buffer, Quenching buffer (BU08-L), Imaging buffer (BU09), and Elution buffer (BU07-L) were freshly made and loaded into the fluidics compartment of the instrument. Fields of view (FOVs) of 12 mm × 12 mm were captured in a tiled fashion, only where the tissue was auto detected. The primary antibodies were used at the following dilutions: 1:1000 GFP (Abcam, ab13970), 1:300 HMGA2 (8179S, CST), 1:100 Moesin (ProteinTech, 26053–1-AP), 1:100 p19 (SantaCruz, sc-32748). The secondary antibodies were used at the following dilutions: 1: 100 Donkey anti-Rabbit AlexaFluor Plus 555 (Thermo Fisher Scientific, A32794), 1:200 Donkey anti-Rabbit AlexaFluor Plus 647 (Thermo Fisher Scientific, A32795), 1:200 Donkey anti-Rat AlexaFluor Plus 647 (Thermo Fisher Scientific, A48272), 1:200 Goat anti-Chicken AlexaFluor Plus 647 (Thermo Fisher Scientific, A32933).

##### Multiplexed Immunofluorescence using Leica CellDive

We used the Leica CellDive imaging system to conduct multi-IF experiments through cycles of staining, imaging and bleaching of fluorescent stains. This allowed us to probe for multiple microenvironment markers in the same tissue section while bypassing limitations of isotype incompatibilities, and leveraging the computational removal of autofluorescence in this imaging system. We imaged 5-μm FFPE sections following the manufacturer’s protocol. Briefly, after a 2-step antigen retrieval process, slides were blocked with 3% BSA, stained with DAPI, and imaged unstained to acquire background autofluorescence (AF). Samples were then stained and imaged using DAPI, Cy3, Cy5, and FITC channels on the CellDive (Leica) instrument with CellDive image acquisition and processing software. Each FOV was imaged in each staining round, followed by AF removal, registration with baseline DAPI, and stitching. Unconjugated primary antibodies were used in the first staining round, followed by secondary antibody staining: GFP (Abcam, ab13970; RRID:AB_300798, 1:1000), HMGA2 (Cell Signaling Technology, 8179S; clone D1A7, RRID:AB_11178942, 1:500) and Tenascin-C (R&D systems, MAB2138; RRID:AB_2203818, 1:250). After imaging, dye inactivation was performed using 0.1 M Na_2_CO_3_ 3% H_2_O_2_ solution for 15 min at room temperature, followed by 1 h blocking with rabbit serum (Sigma-Aldrich, R9133) at room temperature and washing with 1X PBS-T, before starting the next round of AF imaging and staining. Subsequent rounds of staining were conducted with primary antibodies conjugated to a fluorophore, and included the immunosuppressive myeloid cell marker ARG1 (Cell Signaling Technology, 35298; AlexaFluor 555 conjugated, 1:100). All rounds of imaging and slide storage were done in a solution of PBS with 50% glycerol. Staining quality and fluorescence removal were verified after each round. The fully stitched images were imported into HALO^®^ image analysis software (Indica Labs) for visualization.

##### Single-molecule FISH

###### Coverslip preparation for smFISH

Coverslips were prepared as described^12^. Briefly, 40-mm–diameter #1.5 coverslips (Bioptechs, 0420–0323-2) were cleaned in batches by arranging in a wafer boat (Entegris, A23–0215) and immersing in a 1:1 mix of 37% HCl and methanol at room temperature for 30 min. Coverslips were then washed twice with Milli-Q water, and once with 70% ethanol, followed by gentle drying with nitrogen gas. Cleaned coverslips were coated with a silane layer to allow stabilization of a polyacrylamide gel during smFISH staining, following published protocols^12^: they were submerged in 0.1% (vol/vol) triethylamine (Millipore, TX1200) and 0.2% (vol/vol) allyltrichlorosilane (Sigma, 107778) in chloroform for 30 min at room temperature, washed once with chloroform, washed once with 100% ethanol and dried using nitrogen gas. Coverslips were stored long-term in a desiccated chamber.

To prepare for staining individual samples, silanized coverslips were coated with 0.1 mg mL^−1^ Poly-D lysine (Thermo Fisher Scientific, A3890401) at room temperature for 1 h in a 6-cm tissue culture plate. They were then washed once with 1X PBS, and 3 times with nuclease-free water. Coverslips were lifted after each wash, using either tweezers or a needle, to ensure that both sides of the coverslips were exposed to the solution. Coverslips were left to dry for at least 2 h in a tissue culture hood before proceeding to tissue sectioning.

###### Tissue sectioning, fixation and permeabilization

Tissue section preparation was conducted following a published protocol^11^. Briefly, 10-μm tissue sections were cut using a cryostat and mounted into poly-D lysine coated coverslips, then placed face-up on a 6-cm tissue culture dish for all subsequent wash and incubation steps. Coverslips were dried for 5–10 min at 50°C, and placed on dry ice until all samples were sectioned. Next, plates with coverslips were transferred to ice, and treated with 3 mL 1X PBS to melt the OCT, and fixed at room temperature with 4% PFA 1X PBS for 10 min. Coverslips were then washed three times with 1X PBS, and treated with ice-cold 70% ethanol and maintained at 4°C overnight for permeabilization.

###### Pre-staining treatment of permeabilized tissues

After overnight ethanol incubation, coverslips were rehydrated with 1X PBS on ice for 10 min. To bleach endogenous fluorescence of lineage reporters, tissues were exposed to a bleaching solution of 3% hydrogen peroxide (Fisher, H325–500), 1:600 37% HCl (vol/vol) 1X PBS, and placed under a heat lamp for 1 h^13^. They were then washed twice with 1X PBS and once with 2X SSC. Next, they were treated with pre-warmed (37°C) digestion solution containing 20 μg mL^−1^ proteinase K (Sigma, 3115836001) in 2X SSC, and incubated at 37°C for 10 min. This step enhances the permeabilization of probes in an optimized protocol for RNA staining in pancreatic tissue^11^. To remove proteinase K, coverslips were washed 3 times with 2X SSC. To prepare coverslips for hybridization, they were treated with pre-hybridization solution, composed of 30% formamide (Thermo Fisher Scientific, AM9344) in 2X SSC and incubated for at least 3 h at 37°C, as previously described^11^.

###### Staining with primary probes

Computational probe design is described below (Probe design for multiplexed smFISH). Primary probes were diluted at a 100 nM final concentration per probe in 3H staining buffer, composed of 30% formamide, 10% dextran sulfate (Sigma Aldrich, D8906–50G), 1 mg mL^−1^ yeast tRNA (Thermo Fisher Scientific, 15401029) in 2X SSC^12^. In addition, this staining solution had a final concentration of 2 μM anchor probe, a 15-nt sequence of alternating dT and thymidine-locked nucleic acid (dT+) with a 5′-acrydite modification (Integrated DNA Technologies), designed to anchor all polyadenylated RNAs to a polyacrylamide gel in subsequent steps. Next, hybridization chambers were prepared by attaching parafilm on the surface of a 6-cm tissue culture dish. Upon completion of pre-hybridization incubation, a 100-μL droplet of hybridization solution and probes (100 nM per probe) was placed on the center of the hybridization chamber, and coverslips were placed face down so that the hybridization solution uniformly covered the tissue, taking care of removing bubbles that may have formed in the parafilm–coverslip interface. Hybridization chambers were placed on a 15-cm dish, with a wet Kimwipe used as a humidity buffer, and incubated at 37°C for 36h–48 h.

###### Post-hybridization wash

Upon completion of incubation with primary staining solution, post-hybridization wash buffer composed of 30% formamide in 2X SSC was prepared, and pre-heated to 37°C. Coverslips were then washed face-up with post-hybridization wash buffer at 47°C for 30 min. This washing step was repeated for a second 30-min incubation with fresh post-hybridization wash buffer. Lastly, coverslips were transferred to 2X SSC solution and maintained at 4°C until the next step.

###### Gel embedding

Samples were embedded on a thin layer of polyacrylamide gel, to allow subsequent tissue-clearing through digestion of protein and lipids. To prepare the workspace for gel embedding, microscope glass slides (Premier, 6101) were washed with 70% ethanol and RNAse away (Thermo Fisher Scientific, 21–402-178), placed on a benchtop, and covered with 0.5 mL gel slick (Lonza, 50640), cleaning excess with a Kimwipe. The gel solution was composed of 4% (vol/vol) of 19:1 acrylamide/bis-acrylamide (BioRad, 1610144), 60 mM Tris·HCl pH 8 (Invitrogen, 15568–025), 0.3 M NaCl (Boston Bioproducts, R-244), supplemented with the polymerizing agents ammonium persulfate (Sigma, 09913) and TEMED (Sigma, T7024) at final concentrations of 0.03% (wt/vol) and 0.15% (vol/vol), respectively, as described^12^. The solution was then degassed using a vacuum chamber (Thermo Fisher Scientific, 53050609) until bubbles stopped rising to the surface of the solution. Coverslips were rinsed twice with gel solution. A 100-μL droplet of gel solution was placed on a glass slide, and coverslips were placed face-down on the slide so that the gel solution spread evenly at the slide-coverslip interface. Polymerization was completed in 2 h at room temperature, after which gel-embedded coverslips were lifted from the glass slide with the aid of a razor-blade, and transferred to a 6-cm tissue culture dish with 2X SSC.

###### Digestion

Gel-embedded samples were subjected to an overnight treatment with digestion solution, aimed at clearing proteins and lipids from the samples, improving the signal to noise for RNA detection. Digestion solution was composed of 2% SDS (Invitrogen, AM9822), 0.25% TritonX (Acros organics, 327371000), 1:100 dilution of proteinase K (New England Biolabs, P8107S) in 2X SSC. Samples were incubated overnight in digestion solution at 37°C. Following overnight digestion, samples were rinsed once with 2X SSC, transferred into a separate plate with 2X SSC, and washed for 30 min with gentle agitation. The 2X SSC solution was replaced, for a second 30-min wash.

###### Staining with secondary probes

We used readout probes constituted by a 20-bp oligonucleotide conjugated to a fluorophore (Alexa Fluor 488, Cy3B, Cy5 or Alexa Fluor 750) via a disulfide bond. Fluorescent conjugated probes were purchased from Biosynthesis Inc. The secondary staining solution was composed of 5% ethylene carbonate (Sigma Aldrich, E26258–100G) in 2X SSC, and supplemented by 3 nM of a secondary readout probe for each fluorescent color and 1 μM DAPI. Secondary staining was conducted following the same procedure for the primary staining step with the exception that it was conducted for 20 min at room temperature, covering samples with aluminum foil. Following hybridization, samples were washed once with a 10% ethylene carbonate 2X SSC solution for 20 min with gentle agitation, and three times with 2X SSC for 5 min per wash.

###### Iterative smFISH imaging

We prepared the following buffers for iterative smFISH imaging: (1) Wash buffer: 10% ethylene carbonate 2X SSC, 2.5 mL per staining round; (2) Cleavage buffer: 10% TCEP (Sigma-Aldrich, 646547-10X1ML) 2X SSC, 3 mL per cleavage round. TCEP in the cleavage buffer allows reduction of disulfide bond linking fluorophores to oligonucleotides in readout probes for rapid extinction of fluorescent signal; (3) Imaging buffer: 10% glucose 2X SSC, supplemented with catalase (Sigma-Aldrich, C3515; 17.5 μg mL^−1^ final concentration) and glucose oxidase (Sigma-Aldrich, G2133; 1.4 mg mL^−1^ final concentration), 2 mL per imaging round. Imaging buffer was stored under a layer of 1.5 mL mineral oil to minimize oxygen in solution during sequential rounds of staining and imaging; (4) 2X SSC, 40–50 mL per experiment. Furthermore, we prepared readout probe mixes for each round of staining. Readout probes were diluted to a final 3 nM concentration per probe, in 5% ethylene carbonate 2X SSC, supplemented with Murine RNAse inhibitor (New England Biolabs, M0314S; 1:400 dilution). Buffers and readout probe mixes were loaded into a custom-build fluidics control system^14^ that can interface with the NIS Elements image acquisition software (v 5.31.02) using custom macros.

Coverslips were mounted in a commercial flow chamber (Bioptechs, FCS2) sandwiched between a 0.75-mm-thick flow chamber gaskets (Bioptechs, 1907–100; DIE# F18524), a micro-aqueduct slide (Bioptechs, 130119–5NC) and a second 0.75-mm-thick flow chamber gaskets (Bioptechs, 1907–100; DIE# 449673-A), as described^15^. We first cut the gel so that it would fit in its entirety within the rectangular opening of the flow chamber gasket. We placed the flow chamber for imaging on a Nikon Ti2 inverted microscope using the FCS2 stage adapter (Bioptechs, 060319–2-2611), and used our fluidics system to flow in 20X SSC into the sample in order to eliminate bubbles in the tubbing and chamber. Next, we flowed imaging buffer into the sample and generated a low magnification map of the entire tissue using a 20X Plan APO objective (Nikon, MRD00205). We then switch objectives to a high magnification 60X Plan APO immersion oil objective (N.A. 1.4, W.D. 0.13 mm, F.O.V. 25 mm, Nikon, MRD01605) to resolve individual mRNAs. We used tape to minimize the movement of the plate-holder during sequential rounds of imaging, which we found to be important to prevent positional drift throughout the experiment.

Imaging cycles were conducted using the following parameters:

- Staining. Flow staining buffer for 4 min at a rate of 0.5 mL min^−1^. Incubate for 20 min.
- Wash. Flow wash buffer for 5 min at a rate of 0.4 mL min^−1^.
- Imaging. Flow imaging buffer for 3 min 40 sec at a rate of 0.5 mL min^−1^. Take 7 z-stacks per field of view, using a 1-μm step size, for a coverage of −3 μm to 3 μm around the mid-plane, using perfect focus throughout the entire experiment.
- Cleavage. Flow cleavage buffer for 4 min at a rate of 0.5 mL min^−1^. Flow cleavage buffer for 10 min at a rate of 0.1 mL min^−1^. Incubate for 10 min. Flow 2X SCC for 5 min at a rate of 0.5 mL min^−1^.

We collected images from FOVs without tissue or sources of bright autofluorescence that would allow us to estimate non-uniform illumination and detection profiles in each fluorescent channel, and correct for these in downstream image processing steps.

##### Spatial transcriptomics using Xenium

FFPE blocks were sectioned and processed according to 10x Genomics user guidelines (CG000580, CG000582, CG000584). Briefly, 5-μm tissue sections were trimmed from FFPE blocks and placed within the fiducial frame of the Xenium slide (PN-1000460). The slides were air dried, baked at 42°C for 3 h and stored in a desiccator. Tissues were then deparaffinized, rehydrated and de-crosslinked using Xenium Sample Prep Reagents (PN-1000460). Tissues were hybridized overnight using a custom probe set (480 gene panel). The probes were ligated and amplified *in situ*. Tissues were quenched to remove autofluorescence and counterstained with DAPI. Slides with their corresponding decoding file were loaded and imaged on the Xenium instrument.

Table S5 details the samples we analyzed with the Xenium platform.

### II Computational Methods

#### GENE EXPRESSION SIGNATURE DERIVATION FROM EXISTING DATA

##### Premalignant state signatures

To derive gene expression signatures for major premalignant subpopulations, we computed pairwise differential gene expression between discretized premalignant states as defined by Burdziak, Alonso-Curbelo and colleagues^10^. We used the wald test in the diffxpy package (v0.7.4, https://github.com/theislab/diffxpy?tab=readme-ov-file) and library size as numeric covariates. We identified upregulated genes using the following thresholds: *qval* < 0.05, log_2_ fold-change > 1, mean expression > 0.05, and defined a signature as the set of genes upregulated in a specific subpopulation in every pairwise comparison between premalignant states.

##### SMAD4-dependent TGFβ induced genes

We reanalyzed published bulk RNA-seq data from SMAD4-proficient and SMAD4-deficient PDAC organoids stimulated with TGFβ or vehicle^16^. We used the R DESeq2 package (v1.32.0)^17^ to model gene counts as a function of treatment (TGFβ stimulation of vehicle) and SMAD4 status (SMAD4-proficient or SMAD4-deficient). We identified genes that are upregulated by TGFβ stimulation in a SMAD4-proficient context. Furthermore, we required that upregulation was sensitive to SMAD4 status. We used the following thresholds to identify upregulated genes: *padj* < 0.001 and *log2FoldChange* > 1.5, resulting in a gene signature of 88 genes.

##### Glycolysis (Warburg) signature

This signature is composed of a curated list of glycolysis-related enzymes (*Hk1, Hk2, Gapdh, Pgk1, Eno1, Pkm, Ldha*), including glucose, lactate and pyruvate transporters upregulated during the Warburg effect (*Slc16a1, Slc16a3, Slc2a1*)^18^, as well as the hypoxia master regulator *Hif1a*.

##### Public transcriptional signatures

The following table specifies the sources of other signatures used in this study. Signatures are provided in Data S3.

**Table:** 

Signature id	Description	Source
p53 Fisher	Curated targets of the tumor suppressive transcription factor p53	28288132
p53 TSAG	Effectors of p53-dependent tumor suppression that are also bound by p53 in irradiated MEFs	33157015
p53 restoration	Upregulated upon p53 restoration in PDAC cells *in vitro*	31534224
Senescence UP	Upregulated in IMR90 human fibroblasts upon Hras^V12^-induced senescence	27099234
HALLMARK EMT	Genes defining epithelial-mesenchymal transition, as in wound healing, fibrosis and metastasis	26771021
Kras signaling/Fosl1	Consistently upregulated genes in Kras mutant vs wild-type mouse and human tumors	28220783
Kras injury	Genes upregulated in Kras^G12D^+ pancreatic epithelial cells, compared to Kras^WT^ cells, both harvested 48h post-acute pancreatitis *in vivo*	33536616
GOBP wound healing	The series of events that restore integrity to a damaged tissue, following an injury	GO: 0042060
YAP signature	Genes activated by YAP overexpression in human mammary cells (MCF10A), and YAP overexpression in mouse liver tissues or in immortalized mouse fibroblasts	22078877
IFNγ response	Genes up-regulated in response to IFNG (HALLMARK Gene Sets)	26771021
p65-dependent	Upregulated in IMR90 human fibroblasts upon Hras^V12^-induced senescence, dependent on p65 proficiency	27099234

#### PROCESSING AND ANALYSIS OF SINGLE-CELL DATA

##### Data preprocessing and quality control

###### mRNA count matrix generation and demultiplexing

All scRNA-seq datasets were demultiplexed, barcode-corrected, aligned and UMI-corrected with SEQC^19^ using mouse genome mm10 and default parameters for samples generated using the v2 (spontaneous tumorigenesis samples) or v3 (injury-induced tumorigenesis samples) 3’ scRNA-seq kit. We summed all counts from genes that share the same gene symbol.

For samples subjected to cell hashing, we demultiplexed using an in-house method known as SHARP (https://github.com/hisplan/sharp). Hash labels were assigned to either identify a cell as belonging to a specific mouse or as a doublet or low-quality droplet. Doublet calls informed our cluster-based doublet filtering (see below). We excluded non-doublet cells without an assigned hash barcode from sample-specific analyses, but included them in condition-level analyses. This is possible because cells from distinct conditions (experimental time point × genotype) were sequenced independently, and our cell hashing strategy aimed to distinguish biological replicates within a condition.

###### Empty droplet removal and ambient RNA subtraction

We removed empty droplets using the remove-background function of cellbender (v0.2.0)^20^, with *expected_cells* = 5000 for spontaneous tumorigenesis datasets, and 8000 for injury-induced tumorigenesis datasets (based on the number of cells targeted for encapsulation, *total*-*droplets*-*included* = 20,000, *fpr* = 0.01 (default), *learning*-*rate* = 0.0001 (default) and *epochs* = 150 (default). We excluded droplets with fewer than 100 mRNA counts as input into subsequent quality control (QC) analyses, and used cellbender background-corrected count matrices for downstream applications. Ambient RNA subtraction was important for mitigating the effect of CD45+ cell spike-ins during the collection of rare pre-tumor *p53*-deficient cells, as revealed by inspecting immune-related transcripts in epithelial cells (not shown). Unless otherwise stated, we used the cellbender background-corrected count matrix for downstream analyses. We aggregated counts from genes that share the same gene symbol through summation. Preprocessed datasets published as part of our study contain raw and cellbender-corrected counts in the same AnnotationData object for ease of comparison.

###### Low-quality cell removal

For each sample, we used an iterative clustering-based approach to identify and remove low quality groups of cells. During each iteration, we applied scanpy (v1.9.1)^21^ to embed single-cell transcriptomes and identify clusters using standard library size normalization (sc.pp.normalize_per_cell), log transformation with pseudocount 1 (sc.pp.log1p), feature selection (sc.pp.highly_variable, *flavor* = ’seurat’ and default parameters), dimensionality reduction using PCA (*n_comp* = 100), kNN construction (sc.pp.neighbors, *num_neighbors* = 15) and visualization with UMAP (sc.tl.umap). Next, we used PhenoGraph to identify single-cell clusters^22^ (sc.external.tl.phenograph, *clustering_algo* = ‘leiden’) varying the parameter *k* during kNN construction (*k* = 10, 30), resulting in cluster assignments with different levels of resolution.

We removed the groups of cells with lowest summary QC metrics per cluster at each iteration, and stopped excluding when log_lib_size reached 7.5 and percent_mito fell below 20%. By varying cluster resolution, we could identify small clusters of low-quality cells that would otherwise be merged into large clusters. For some samples, we computed high-resolution clusters using *k* = 5 during the last iteration of cluster-based QC. We found that two or three iterations of this procedure per sample was sufficient to satisfy our bounding criteria, and removed all clusters with outlier QC metrics.

###### Doublet and contaminant identification

We used doubletdetection (v4.2, http://doi.org/10.5281/zenodo.2678041) with default parameters to infer doublets, using raw counts as input. For samples subjected to cell hashing, we consolidated computationally inferred doublets with doublets identified through the detection of two hash ids. Using PhenoGraph cluster assignments with different resolutions (*k* = 5, 10, 30), we identified and recorded clusters in which at least 50% of cells were inferred as doublets. At this preprocessing step, we only recorded doublets, but didn’t exclude them from the dataset. We reasoned that maintaining these annotations would be helpful for cross-sample identification of double-enriched clusters, as we have previously shown^10^.

Although our single-cell data was derived from epithelial cells sorted by fluorescent protein expression, we identified single-cell clusters corresponding to immune and stromal contaminants. We used gene expression marker of major cellular compartments to identify these clusters (*Col1a1* for fibroblasts, *Ptprc* for immune cells, *Pecam1* for endothelial cells, *Des* for pericytes), in combination with the absence of epithelial markers (*Epcam* and *Cdh1*, as well as mRNAs corresponding to fluorescent proteins used during sorting, *GFP* and *rtTA3*-*IRES*-*mKate2*). Similar to our strategy with doublet handling, we only annotated, but not excluded these clusters at this stage of analysis in an effort to identify contaminants in other samples from the same batch that are too rare to form a single cluster.

###### Gene exclusion for post-cleaning preprocessing

We excluded the following classes of genes for normalization and feature selection: (i) mRNAs corresponding to fluorescent proteins and shRNAs engineered into our mouse model, (ii) mitochondrial and ribosomal transcripts, (iii) the lncRNA *Malat1*, the inclusion of which was previously shown to distort single cell embeddings in our experimental system^10^. In excluding these genes, we aimed to minimize variation stemming from quality control metrics or hard-coded experimental conditions (e.g. inducible expression of a fluorescent protein). In addition, we excluded genes expressed in less than 10 cells across all batches. While we excluded these genes during single-cell embedding, we kept them in the count matrix, so that the information they contained could be used in downstream analyses.

###### Within-batch data consolidation

As the final step of QC and preprocessing, we merged count tables and annotations of all samples from the same batch. We merged AnnotationData objects using the concat function of this class with *join* = ‘outer’ (include genes present in any sample) and *fill_value* = 0 (assume that a gene not present in a sample has expression of 0). We computed within-batch single-cell embedding and clustering using the strategy detailed for single sample filtering. We used cluster-level doublet annotations of individual samples to exclude doublet-enriched clusters (those in which >50% of cells were predicted to be doublets). Similarly, we removed clusters enriched in cells annotated as contaminants.

Lastly, we used outlier detection and hard thresholding to exclude a small number of cells with low QC metrics that were not identified using cluster-based exclusion. Specifically, we computed two QC metrics for each cell, log-transformed library size and log-transformed number of detected genes (excluding ribosomal, mitochondrial, fluorescent protein and shRNA mRNA counts). Although correlated, these two metrics provide orthogonal information about transcriptional complexity, as a cell may pass a library size-based threshold even when few genes dominate its mRNA counts. To identify outliers whose QC metrics deviate from those of their nearest neighbors, we used the LocalOutlierFactor function from the sklearn.neighbors package (v1.0.2) with *n_neighbors* = 100 and *contamination* = 0.1 (number of assumed outliers). In the final step, we excluded low-quality cells based on log-transformed library size < 7.5, fraction mitochondrial counts > 0.2, or number of detected genes < 300.

###### Preliminary tumor and premalignant annotations

After integrating samples within batches, we identified clusters that exhibited transcriptional and genomic patterns consistent with being derived from PDAC (see Cell state annotation and Copy number inference for details). Conducting these preliminary annotations within individual batches was important for determining which cells to exclude from subsequent batch correction vector calculations.

##### Analysis of KP^LOH^ spontaneous tumorigenesis data

###### KP^LOH^ dataset details

Our spontaneous tumorigenesis data from KP^LOH^ mice charts PDAC progression through the benign-to-malignant transition, and is composed of three conditions: (1) *p53*-proficient cells from mice without a macroscopic tumor (pre-tumor *p53*-proficient), (2) *p53*-deficient cells from mice without a macroscopic tumor (pre-tumor *p53*-deficient), and (3) *p53*-deficient cells from mice with a macroscopic tumor (tumor *p53*-deficient). Tumor *p53*-deficient cells are derived from (i) tumor-bearing KP^LOH^ mice, isolated as GFP-/mKate2+ cells (see Mouse model genetics for details) and (ii) samples from tumor-bearing mice from Burdziak, Alonso-Curbelo and colleagues ^10^. For batch 8, we pooled multiple mice without hashing to minimize the time between harvesting and sorting during single-cell isolation. While we lack cell-to-mouse assignments in this sample, we note that pre-tumor cells generally do not cluster by biological replicate (Fig. S1a). Table S1 summarizes the number of cells per sample in our spontaneous tumorigenesis dataset.

Our prior data set^10^ did not include a reporter of *p53* genetic status; thus, a subset of cells from PDAC samples co-embedded with premalignant cells during integration. We also observed that a small fraction of cells sorted as *p53*-deficient from the KP^LOH^ model co-embedded with pre-malignant cells and expressed GFP mRNA, implying that they were indeed *p53*-proficient. We filtered out such contaminants from our dataset before embedding all samples. Note that most premalignant contamination comes from primary tumors rather than metastases, supporting the notion that these were non-cancer cells embedded within the tumor.

**Table:** 

Sample ID	Sample codename	Source (PMID)	Primary or metastasis	No. cells in pre-malignant clusters
DAC_D020_p5_Epi	Tumor p53 deficient 11	37167403	Primary	645
Ag-PDAC-PT-Kate	Tumor p53 deficient 10	37167403	Primary	180
53_LHRH_PDACreg_KATE	Tumor p53 deficient 7	This work	Primary	14
DACC963PT_Kate_plus	Tumor p53 deficient 9	37167403	Primary	7
PDAC-SP3	Tumor p53 deficient 6	This work	Primary	5
9268_PHLH_PDAC_SP	Tumor p53 deficient 8	This work	Primary	2
Ag-Lung-Mets-Kate	Tumor p53 deficient 10	37167403	Metastasis	2
DACC963LIVERmet	Tumor p53 deficient 9	37167403	Metastasis	0
DACC963_mKate_plus	Tumor p53 deficient 9	37167403	Metastasis	0

###### Feature selection and normalization

To identify a set of highly variable genes (HVGs) that capture the variability across samples in all 3 batches, we conducted within-batch feature selection using scanpy’s sc.pp.highly_variable_genes function with *n_top_genes* = 3000 and *flavor* = ‘seurat_v3’. The final set of HVGs comprised the union of genes selected in each batch, resulting in 5159 genes capturing variation along PDAC progression.

We conducted a batch-aware normalization approach similar to Haghverdi and colleagues^23^. We estimated per-cell size factors as the total counts after excluding mitochondrial, ribosomal, transgenic and *Malat1* mRNAs (see Gene exclusion for post-cleaning preprocessing), then calculated the median size factor per batch, and rescaled size factors to equalize medians across batches. We normalized data by dividing counts by rescaled size factors, and multiplying by the median rescaled size factor across all cells. Lastly, we applied a log transformation to the count matrix with pseudocount = 1.

###### Dimensionality reduction, batch correction and KNN construction

We computed a batch-corrected latent space for subsequent processing steps using mutual nearest neighbors in the batchelor R package (v1.8.1)^23^ with batches 1 and 2 as reference. These batches contained the majority of cells in the dataset, and spanned all timepoints and genotypes. We used the fastMNN function with *cos.norm* = FALSE, *d* = 100 (number of components to keep), *correct.all* = TRUE and *prop.k* = 0.1 (default), resulting in a corrected latent space of 100 components that capture 45% of reference batch variance. Although the inflection point in the cumulative explained-variance curve was at 62 PCs (explaining 42% of variance), we chose to keep more PCs because this dataset was composed of both cancer and premalignant states, and our subpopulations of interest (e.g. progenitor-like cells) were rare (1–2% of premalignant cells).

We computed this latent space on log-normalized counts, using only HVGs. In addition, when calculating correction vectors, we excluded cells corresponding to PDAC clusters; we previously showed that each tumor forms a distinct cluster in this model and reasoned that including PDAC cells could remove and distort true biological heterogeneity in the dataset. We note, however, that these clusters were subjected to batch correction using correction vectors estimated from non-PDAC cells. The count matrix remains unmodified in this approach. Lastly, we constructed a *k*-nearest neighbor graph (kNN) using the batch-corrected latent space as input to scanpy’s sc.pp.neighbors function, using *num_neighbors* = 30.

###### Single-cell data visualization

To visualize our single-cell data, we first appliedUniform Manifold Approximation and Projection (UMAP) for dimensionality reduction using scanpy’s sc.tl.umap function and the precomputed kNN graph as input (*k* = 30). We also computed a force-directed layout (FDL), which captures continuities in data and highlights transitional subpopulations in dynamic systems, using the forceatlas2 python package^24^. We used the diffusion operator of our single-cell data as input to compute force-directed layouts (Note S1). This strategy incorporates local changes in distances along the single-cell manifold into graph visualization.

###### Copy number inference

We inferred karyotypes from single-cell transcriptomes to better distinguish PDAC from premalignant status, and to characterize genomic diversification following spontaneous *p53* loss. To infer chromosome-level changes, we used a custom implementation of inferCNV (inferCNV of the Trinity CTAT Project. https://github.com/broadinstitute/inferCNV), as outlined below. inferCNV assumes that changes in copy number cause corresponding changes in gene expression which, though subject to variation by cell state and by other factors, can be detected as concerted changes in the expression of genes in local genomic neighborhoods.

We first selected a *p53*-proficient sample^1^ as a diploid or near-diploid reference, and computed the mean expression of each gene from library size normalized counts (without log transformation). We excluded genes with low expression (*min_threshold* = 0.1), reasoning that they are less likely to reveal robust gene expression differences caused by genomic changes. Next, we ordered genes by genomic coordinates (UCSC mm10). For each gene × cell pair in the KP^LOH^ dataset, we computed the log_2_ fold-change in gene expression (*pseudocount* = 0.1 for both numerator and denominator) over the mean expression of that gene in the reference cell set, clipping log_2_ fold-change estimates to [−3, 3] to limit the effect of outliers. We computed the sliding average log_2_ fold-change over a window of consecutive genes in the same chromosome (*window_size* = 100), trimming chromosome ends. Lastly, we recentered average log_2_ fold-change expression profiles by subtracting the values of each cell by their median, resulting in our final proxy for copy number changes. To cluster inferred karyotype profiles, we computed a simplified matrix, in which each cell is described by the average log_2_ fold-change expression of each chromosome. We clustered this simplified matrix using hierarchical clustering (*method* = ‘ward’, *metric* = ‘euclidean’) implemented in the cluster.hierarchy module of scipy (v1.7.3).

Our approach incorporated two modifications to the standard approach. First, our initial examination of inferred copy number profiles revealed that small groups of biologically related genes could distort estimates. For example, we identified a cluster of carboxypeptidases (*Cpa1, Cpa2, Cpa5, Cpa4*) on mouse chromosome 6 that are expressed at high levels in acinar and ADM cells, causing spikes in inferred copy number that could be mistakenly interpreted as focal amplification. We therefore removed all such gene groups from copy number inference through manual inspection of spikes in gene smoothed gene expression profiles with smaller window sizes (5–20 genes), as well as ribosomal and mitochondrial genes (see Data S3 for excluded genes). Our approach prioritizes robust estimation of chromosome-level copy number changes over the identification of more focal alterations and highlights an important opportunity for feature selection in developing copy number inference strategies.

Second, we incorporated iterative copy number inference to mitigate batch effects during karyotype estimation. We could use within-sample diploid references for pre-tumor stage inference, because we compared *p53*-proficient samples (expected to be near diploid) with *p53*-deficient samples. Notably, the only recurrent change in pre-tumor *p53*-proficient cells was the gain of chromosome 6, as reported in the KP^LOH^ model^1^. The distribution of the average smoothed log_2_ fold-changes in chromosome 6 was bimodal, providing a natural threshold to identify cells that gained this chromosome (average log_2_ fold-change > 0.17). We used cells without this event as diploid references within each mouse, reducing variability in inferred copy number profiles.

To classify pre-tumor *p53*-deficient cells as genomically ‘quiet’ or ‘rearranged’, we computed a simplified karyotype matrix in which each entry is the average copy number change of each chromosome in each cell. Next, we binarized this matrix by identifying entries > 0.16 (indicating gains) or < −0.16 (indicating losses). We selected these thresholds based on the distribution of average copy number changes across all cells and chromosomes in the dataset. We defined genomically ‘quiet’ cells as those that had less than 9 gain or loss events, and ‘rearranged’ cells as those with more than 9 events. These thresholds captured differences in transcriptional states that distinguished premalignant-like and cancer-like clusters (Fig. 1 and Fig. S2) in the presence of noisy karyotype inference from transcriptomes.

###### Refinement of condition assignment

We used copy number profiles and transcriptome-based PhenoGraph clusters to refine assignment of individual cells to cancer-like or premalignant. First, we identified a rare group of pre-tumor cells sorted as GFP+, but that lacked GFP mRNA expression and chromosome 11 loss (containing the *p53* locus). Given that these are criteria for detecting *p53* deficiency in this mouse model, we re-assigned them as pretumor *p53*-deficient cells (*n* = 4 cells reassigned).

###### Annotation of premalignant states

To annotate cell states in the premalignant pancreas, we first used the scanpy implementation of PhenoGraph sc.external.tl.phenograph (*k* = 30, *clustering_algo* = ‘leiden’) with gene expression signatures from our published dataset^10^ (see Premalignant state signatures). First, we standardized our count matrix by computing the z-score expression of each gene across all cells. To calculate a signature score per cell, we averaged the z-scored expression of signature genes in each cell. We aggregated signature scores at the cluster level by averaging, and standardized such average scores across clusters. Lastly, we used the searborn (v0.11.2) clustermap function to guide manual cluster annotation.

We noted that while some cell states were clearly separated from the bulk of the premalignant epithelium (e.g. ADM, tuft and neuroendocrine cells), the majority of epithelial cells varied along a phenotypic continuum linking gastric-like and progenitor-like states. To capture continuity between these states, we used diffusion component analysis^25,26^, as diffusion components represent axes of variation in the data and can describe successive cell-state transitions along the phenotypic manifold (Note S1). In our spontaneous tumorigenesis dataset, the second diffusion component (DC2) correlated with single-cell progenitor-like scores (Fig. S1g). To identify a boundary between gastric-like states and progenitor-like states along this phenotypic continuum, we used the triangle method to identify a threshold in the distribution of cell densities along DC2 (Fig. S1h). Cell state discretization in light of this continuum was helpful to interpret cell state-specific consequences of spontaneous *p53* loss.

###### Genes upregulated in malignant cells in the KP^LOH^ model

We leveraged our *p53*-proficient and *p53*-deficient single-cell data in the KP^LOH^ background to derive a signature of genes upregulated in PDAC compared to premalignant cells. By adopting a pseudobulking approach for differential gene expression, we could quantify changes in average transcript expression between premalignant and malignant cells in a manner agnostic to the subpopulation structure of each sample, while leveraging inter-replicate variability for the derivation of a robust expression signature. We restricted our analysis to samples from batches 1 and 2 (see Table S1), which were collected simultaneously and sequenced using the same reagents to avoid variation from technical sources. Furthermore, we excluded metastasis samples to focus on pancreas-derived cells. In total, we analyzed 4 pre-tumor *p53*-proficient samples and 6 tumor-derived *p53*-deficient samples. To identify differential expression, we aggregated unnormalized, cellbender-corrected counts per sample to construct a pseudobulk count matrix with genes as rows and samples as columns. Next we used the R package DESeq2 (v1.42.1) to test for differential gene expression between PDAC and premalignant conditions, using *design* = “~ condition” to model counts. We identified upregulated genes as those with padj < 0.001 and log_2_(fold change) > 1.5, resulting in a signature of 941 genes.

###### Simultaneous visualization of multiple signatures

To visualize multiple signatures in the same single-cell layout (Fig. 1e) we used a signature-based pseudo-coloring strategy as previously implemented^10^. We normalized scores for each signature by subtracting the minimum signature value and dividing by the 95^th^ quantile of such scores. We define the signature matrix *S*_*n*×*m*_ such that *S*_*i*,*j*_ is the normalized score for signature_j_ in cell_i_, and we define a color-encoding matrix *W*_*m*×3_ where *W*_*j*,∙_ is the RGB vector representation of the color associated with signature_j_. The pseudo-coloring of a cell is defined by the matrix multiplication *S* × *W*. Because RGB components are bounded between [0,1], we clip values to 1 after matrix multiplication. This approach is most effective in simultaneously visualizing multiple phenotypically distinct subpopulations and a limited number of mixed subpopulations, as pseudo-colors can saturate due to the effect of summation and clipping.

###### Diffusion distance analysis

To quantify transcriptional similarity between premalignant cells and PDAC, we used diffusion distance, a quantity that allows estimation of long-range cell–cell connectivities while respecting non-linearities in the phenotypic manifold (Note S1). To compute diffusion distance between premalignant and malignant cells (Fig. 1f), we used the eigenvectors associated with the 17 highest eigenvalues of the diffusion operator, based on the second eigengap as the threshold criterion. To calculate the similarity between pre-tumor *p53*-proficient (premalignant) and cancer cells, we computed the diffusion distance from every premalignant cell to the closest cancer cell (annotated as *p53*-deficient tumor or microtumor).

###### Differential gene expression

To assess cell state-dependent consequences of *p53* loss, we computed differential gene expression between pretumor *p53*-proficient cells and pre-tumor *p53*-deficient cells with ‘quiet’ genomes (see Copy number inference for details). We used the wald test in the diffxpy package (v0.7.4, https://github.com/theislab/diffxpy?tab=readme-ov-file), with raw counts as input and library size as a numeric covariate. We identified downregulated genes upon *p53* loss as those with qval < 0.05, log_2_(fold change) < −1 and mean expression > 0.05. To visualize gene expression as a function of cell state and *p53* status (Fig. 2a) we *z*-scored log-normalized counts using the mean and standard deviation of pre-tumor *p53*-proficient cells as reference. This standardization strategy aims to highlight deviations in gene expression attributed to *p53* deficiency and doesn’t depend on inclusion or exclusion of tumor-stage samples.

###### Quantification of p16^INK4A^ and p19^ARF^ isoforms

The *Ckdn2a* locus encodes two structurally and functionally distinct gene products, p16^INK4A^ and p19^ARF^, both of which mediate tumor suppression through different mechanisms. cDNAs for these gene products result from alternative use of the exon 1α (p16^INK4A^) or exon 1β (p19^ARF^), spliced into the shared exon 2. Most reads captured through 3’-end sequencing are unable to distinguish between these two gene products, but we found rare reads that spanned isoform specific splice junctions, allowing unambiguous determination of *Cdkn2a* isoforms. To identify such reads, we used our custom scRNA-seq processing pipeline SEQC to scan aligned bam files from pre-tumor *p53*-proficient samples for reads that (1) fell within the boundaries of exon 1β and exon 2 of the *Cdkn2a* locus (chr4:89276895–89276975), and (2) showed evidence of splicing as evidenced by a gap flag in the CIGAR string. Next, we aligned reads to the spliced p16^INK4A^ or p19^ARF^ sequences, to assess the isoform associated with each read.

##### Processing and analysis of *p53* knockdown data

###### Dataset details

Our injury shp53 cohort contained dissociated single-cell data from *Kras*^G12D^+ epithelial cells collected from KC^shp53^ or KC^shRen^ mice 3 weeks after injury to induce pancreatitis. This dataset, composed of two batches with 2–3 mice per genotype per batch, formed the basis of our investigations on the cell-intrinsic consequences of *p53* loss in the context of pancreatic injury. Table S1 summarizes the number of cells per condition in our *p53* perturbation dataset.

###### Cell filtering

Preliminary embeddings of filtered and merged objects during data cleaning (see Data preprocessing and quality control) revealed a PhenoGraph-defined (*k* = 30) subpopulation of 210 premalignant cells that coexpressed divergent cell-type markers—*Cpa1* for acinar, *Msn* for progenitor-like, *Muc6* for gastric-chief-like and *Anxa10* for gastric-pit-like cells. This subpopulation was present in only one batch, and 95% of its cells were derived from a single biological replicate. We excluded these cells from further analysis because the cluster was not reproducible between biological replicates.

###### Normalization, feature selection and dimensionality reduction

As we did not detect strong batch effects during exploratory analysis, we merged the two batches into the same AnnotationData object and computed embeddings as follows: (1) size factor estimation from library sizes, excluding *Malat1* and ribosomal, mitochondrial and transgenic mRNAs; (2) median equalization of size factors between batches; (3) normalization through division by size factors, scaling by the median size factor estimate, followed by log transformation with *pseudocount* = 1; (4) selection of top 3000 HVGs in each batch using the scanpy sc.pp.highly_variable_genes function with *flavor* = ’seurat_v3’; (5) dimensionality reduction using the scanpy PCA function sc.pp.pca., keeping 68 PCs (explaining 54% of total variance) based on the inflection point in the cumulative explained-variance curve; (6) kNN construction with *k* = 30; and (7) visualization using UMAP and FDL (see Single-cell data visualization). Our embedding recapitulated the structure that we previously identified in our published injury-induced tumorigenesis dataset^10^.

###### Visualization of cell state density in two-dimensional representation

To gain an intuition of how individual cell distributions change along the phenotypic manifold upon *p53* knockdown, we computed two-dimensional densities in the coordinates of our layouts. For each condition (*p53* knockdown or control), we computed a histogram summarizing cellular frequencies at different coordinates of the 2D projection of the UMAP embedding (100 bins for x and y axes). We smoothed the histogram using a 2D Gaussian kernel with *bandwidth* = 1 bin, and visualized the estimated distributions in a contour plot (Fig. 7c). We emphasize that this procedure does not accurately estimate cellular densities in high dimensional space; however, we found it useful for communicating results of high-dimensional computations (e.g. the accumulation of progenitor-like cells with mesenchymal properties upon *p53* knockdown).

###### Differential gene expression

To assess the consequences of *p53* knockdown in premalignant epithelial cells during injury, we adopted a pseudobulking approach. PhenoGraph (*k* = 30) grouped progenitor-like cells into three clusters—one from shRen samples (shRen progenitor 1) and two from shp53 samples (shp53 progenitor 1 and 2) (Fig. S11c). Diffusion component analysis suggested that different progenitor-like subpopulations lie along a phenotypic continuum and that *p53* knockdown facilitates persistence or progression of the more advanced progenitor 2 state.

We reasoned that comparing cluster pairs would allow us to distinguish direct effects of *p53* knockdown that are due to target gene activation from secondary effects that are due to changes in cell state. We therefore asked which gene expression programs change upon *p53* knockdown (1) for all progenitor-like cells, (2) for regions of the progenitor continuum with similar shp53 and shRen cell densities, and (3) that specifically characterize progenitor 2 cells.

We grouped cells by genotype (shp53 or shRen) and progenitor-class (progenitor 1, progenitor 2 or all progenitor-like cells) combination, then summed unnormalized counts to generate a pseudobulk sample. Our approach is conceptually similar to marker-based cell sorting followed by bulk RNA sequencing, and illustrates how differential gene expression results change depending on the resolution at which single-cell communities are computationally or experimentally isolated. To compute differential expression, we used DESeq2 (v1.42.1) with pseudobulk counts as input, and genotype or cell state as contrasts. We used the GSEA implementation of gseapy (v1.1.2) to query gene sets differentially expressed between different progenitor-like clusters in shp53 cells, using the MSigDB Hallmark 2020 database, log_2_ fold-change estimates as the rank variable, and FDR < 0.1 as a significance threshold.

###### Condition-aware imputation and gene signature scoring

We used MAGIC-imputed counts^26^ to compute gene expression signatures. MAGIC uses the diffusion operator (Note S1) to share gene expression information in local neighborhoods in cell state space, mitigating the effect of dropouts in sparse single-cell datasets. Formally, the imputed count matrix is obtained by exponentiation of the diffusion operator, and multiplication with the count matrix. The exponent of the diffusion operator *t* corresponds to the number of diffusion steps (*t* = 3 in our specific implementation). Increasing *t* increases the distance over which gene expression information of any given cell influences imputed counts of another cell in the phenotypic manifold.

To avoid sharing gene expression information between cells from different genotypes, we carried out imputation separately for shp53 and shRen samples, using the same PC space to construct the kNN graph (*k* = 30) as input to the imputation process. To score gene signatures, we first computed a *z*-scored imputed gene expression matrix. We used the mean and standard deviation of imputed counts in shRen samples for *z*-scoring. This strategy aims to highlight deviations in shp53 cells relative to control cells.

###### Diffusion component analysis

Diffusion component analysis captures continuous, non-linear variation in data; we applied it to identify dominant axes of variation in our premalignant pancreas data. We constructed a diffusion operator using a kNN graph (*k* = 30) built on cells from both shp53 and shRen samples. The third eigenvector of the diffusion operator (diffusion component 3 or DC3) captured variability between the two most abundant subpopulations in the premalignant pancreas, consisting of gastric-like and progenitor-like states. To plot gene signatures as a function of diffusion component, we discretized DC3 into 100 equally sized bins and computed the average signature score for each bin, separately for shp53 and shRen genotypes (Fig. 7d). We excluded bins with fewer than 10 cells from visualization.

##### Analysis of KRAS inhibitor data

We aimed to systematically quantify changes in the premalignant epithelium from acute oncogenic KRAS inhibition by MRTX1133 treatment in the context of pancreatic injury. This unbiased characterization complements our targeted analyses of the effects of this treatment on the abundance of progenitor-like cells (Fig. 6b). The three biological replicates from each condition (MRTX1133 or vehicle-treated) were pooled, encapsulated and sequenced together, followed by sample deconvolution using cell hashing.

###### Single-cell embeddings

Starting from QC-filtered count matrices (see Data preprocessing and quality control), we merged data from two conditions into a single object, and generated single-cell embeddings by: (1) size factor estimation from library sizes, excluding *Malat1* and mitochondrial, ribosomal and transgenic mRNAs; (2) standard library size normalization, followed by scaling by median library size; (3) log transformation using pseudocount = 1; (4) selection of top 3000 HVGs using scanpy’s sc.pp.highly_variable function on cellbender counts with *flavor* = ‘seurat_v3’ (excluding mitochondrial, ribosomal, transgene and *Malat1* mRNA); (5) dimensionality reduction using the scanpy PCA function sc.pp.pca., keeping 57 PCs (explaining 51% of total variance) based on the inflection point in the cumulative explained-variance curve; (6) kNN construction using *k* = 30; (7) UMAP visualization using sc.tl.umap and default parameters; (8) computation of diffusion operator on the kNN graph (Gaussian kernel width determined adaptively based on distance to each cell’s 10th nearest neighbor) and (9) FDL visualization using the diffusion operator as input and initialization using UMAP coordinates. We visualized conditions and cell states as 2D density maps projected on our FDLs (Fig. S9b). Table S1 summarizes the number of cells contained in our single-cell object.

###### Cell type annotation

We used PhenoGraph clustering and marker gene expression to annotate premalignant subpopulations. Most subpopulations relied on highly specific established markers (e.g., *Cpa1* for ADM cells, or *Pou2f3* for tuft cells, or *Syp* for neuroendocrine cells). For gastric-like states, we used a refined set of markers, since oncogenic KRAS inhibition led to shifts in the spectrum of these states. To refine gastric-like states, we also used a smaller than typical *k* (*k* = 10) for kNN construction when inferring PhenoGraph clusters. We used the following markers to annotate premalignant states:

**Table:** 

Cell state	Marker genes
Progenitor-like	*Hmga2, Msn, Itga3, Nes*
ADM	*Cpa1, Rbpjl, Nr5a2*
Tuft	*Pou2f3, Alox5, Ptgs1*
Neuroendocrine	*Syp, Scg5, Chga, Chgb*
Cycling	*Mki67, Bub1, Cdk1*
Duct	*Rgs5, Cp, Prox1*
Gastric-general	*Dmbt1*
Gastric pit-like	*Anxa10, Tff1*
Gastric chief-like	*F5, Muc6*

###### Differential abundance analysis

To test for differential abundance of distinct premalignant subpopulations as a result of MRTX1133 treatment, we used the Milo algorithm^27^. This method first identifies communities of cells on a kNN graph that partially overlap between conditions (Milo transcriptional neighborhoods), then models the cell counts from different experimental conditions in each neighborhood using a generalized linear model with negative binomial residuals. This allows testing for differences in the abundance of cells from different conditions within granular cellular states in the data. We used the miloR implementation with a precomputed kNN graph (*k* = 30) and PCA (*n_pcs* = 57), using the makeNhoods function for Milo neighborhood construction with *prop* = 0.01 and *refined* = TRUE. This approach uncovered a set of granular cell states that are either enriched or depleted (SpatialFDR < 0.1) in the premalignant pancreas upon acute oncogenic KRAS inhibition. To visualize these results, we annotated transcriptional neighborhoods by their most common cell state label, and plotted their estimated log-fold change in MRTX1133-treated vs vehicle treated mice as a function of cell state (Fig. S9c). Furthermore, it showed that the progenitor-like state is most dependent on persistent *Kras* signaling among premalignant subpopulations.

###### Differential gene expression analysis

We reasoned that dissecting gene expression changes *within* premalignant states as a consequence of acute oncogenic KRAS inhibition could reveal programs dependent on persistent KRAS signaling and cell-state transitions mediated by loss of oncogenic signaling. We focused on ADM cells, as this subpopulation was not depleted but showed a marked shift in transcriptional state. We used the wald-test in the diffxpy package (v0.7.4, https://github.com/theislab/diffxpy?tab=readme-ov-file) to compute differential gene expression between MRTX1133 and vehicle-treated ADM cells, using library size as a numeric covariate, and filtering to only include genes with mean expression > 0.05 that are expressed in at least 20 cells. These filters aimed to exclude low-expressed genes with potentially large fold changes that do not represent the strongest biological differences between states. To identify molecular programs upregulated or downregulated upon MRTX1133 treatment, we used the GSEA implementation of gseapy (v1.1.2), using log_2_ fold-change estimates as the rank variable, and FDR < 0.1 as a significant threshold. We independently queried multiple databases, including the MSigDB_Hallmark_2020 database for general shifts in cellular signaling and the TF_Perturbations_Followed_by_Expression database to reveal molecular regulators that may mediate cell-state shifts following oncogenic KRAS inhibition. Our focus on these gene sets was motivated by results from the literature, showing that chronic genetic or pharmacological inhibition of oncogenic KRAS signaling leads to restoration of a normal pancreatic histology in the premalignant pancreas^28,29^.

###### Gene expression signatures and statistical analysis

Oncogenic KRAS engages cancer-associated and tumor suppressive responses in the premalignant pancreas; thus, we asked whether removing the signal was sufficient to downregulate these programs. We compared the gene expression signatures used to characterize oncogenic and tumor suppressive responses in premalignant cells during spontaneous tumorigenesis (Fig. 2d) in MRTX1133 and vehicle-treated mice. Specifically, we first standardized log-transformed normalized counts over all cells unambiguously assigned to a specific biological replicate through cell hashing. Then, we computed signature scores per cell by averaging *z*-scored gene expression of signature genes. We grouped cells by biological replicate (Fig. S9f,g), testing for differences in the average signature score as a function of experimental condition using a Two Tailed Wilcoxon Rank Sums test. Note that p-values from this test only consider the rank of observations, explaining why p-values are the same for every comparison in (Fig. S9f). This analysis showed that MRTX1133 treatment reduces the expression of oncogenic and tumor suppressive responses in the premalignant pancreas when considering all cells together. To complement this approach, we grouped cells by treatment and cell state, computing group averages of signature scores. Visualization of these summarized scores in a heatmap (Fig. S9g) revealed that regardless of treatment status, cell state continued to be a dominant variable in shaping oncogenic and tumor suppressive responses in the premalignant pancreas.

##### Processing and analysis of premalignant tumor microenvironment data

We used Flex Gene Expression (10x Genomics) to gain insights into transcriptome-wide heterogeneity in gene expression across cellular compartments in the premalignant pancreas. These data allowed us to contextualize compositional and molecular properties of cellular states associated with the progenitor niche, as identified with Xenium-based measurements, including information regarding the expression of mRNAs that were not measured in our Xenium panel, but that have important roles in myeloid and stromal compartments in the context of tissue injury and cancer.

Our dissociated single-cell data was composed of samples from KC^shp53^ mice (*n* = 2) or KC^control^ mice (*n* = 2, KC^shRen^ or KC^shp53^ without doxycycline induction of shp53). All samples came from tissue harvested 3 weeks post-pancreatitis, followed by nucleus isolation from FFPE blocks (see Dissociated nuclei from FFPE samples). Data from these samples was subjected to (1) QC and embedding of single-cells from all samples, and (2) computation of compartment-specific embeddings.

###### Cell and gene filtering

We used cellbender (v0.3.2) for ambient RNA correction and prediction of empty droplets. Examination of library sizes of cellbender-filtered cells revealed a unimodal distribution of ln(library size) with mean = 7.04 and standard deviation = 1.17. We conservatively filtered out cells with ln(library size) < 5, corresponding to the bottom 3.6% of the dataset. We excluded mitochondrial and transgene mRNAs (GFP and mKate2) from influencing normalization and feature selection. Furthermore, we used doubletdetection v4.2, http://doi.org/10.5281/zenodo.2678041 for prediction of doublets, removing any PhenoGraph cluster (*k* = 30 or 10) in which at least 50% of cells within the cluster were predicted to be doublets. Table S1 summarizes the number of cells included in this dataset for each biological replicate.

###### Embedding of single cells from all samples

We generated single-cell embeddings as for other datasets, by (1) size factor estimation from library sizes, excluding *Malat1* and mitochondrial, ribosomal and transgenic mRNAs; (2) standard library size normalization, followed by scaling by median library size; (3) log transformation of count matrix with pseudocount = 1; (4) selection of top 3000 HVGs using scanpy’s function sc.pp.highly_variable with *flavor* = ‘seurat_v3’ (excluding mitochondrial, ribosomal, transgene and *Malat1* mRNA); (5) dimensionality reduction using the scanpy PCA function sc.pp.pca., keeping 100 PCs (explaining 53% of total variance). We used many more PCs than the knee point of the cumulative explained-variance curve because we sought to capture both intercompartment and intracompartment heterogeneity in this dataset, containing the full diversity of premalignant cell types; (6) kNN construction using *k* = 30; (7) Computation of PhenoGraph clusters with different resolutions (*k* = 30 or 10), using Leiden for clustering of the Jaccard similarity matrix; (7) visualization using UMAP. This strategy resulted in a consolidated dataset for the extraction of select cellular compartments for further analysis.

###### Compartment and condition-specific embeddings

Our main goal in collecting and analyzing this dataset was to provide a transcriptome-wide contextualization of the gene expression changes that we identified in Xenium-based spatial transcriptomics data. Thus, it was important to compute compartment-specific and condition-specific embeddings. We focused specifically on fibroblast and myeloid compartments, which represent the two most abundant microenvironmental cells in the premalignant pancreatic parenchyma, and those in which we identified the strongest changes in gene expression as a function of which niche they were encountered in. To compute these embeddings, we (1) isolated subpopulations based on condition filtering and identification of PhenoGraph clusters labeled by marker genes; we followed the strategy outlined above (Embedding of single cells from all samples), except that we used an adaptive strategy for determining the number of PCs to retain at each step, based on the inflection point in the cumulative explained-variance curve. Table S3 summarizes the intermediate processing steps that we took to compute compartment and condition-specific embeddings.

##### Analysis of human pancreatic epithelial data

###### Computation of *p53* activity and gene expression signatures

To investigate *p53* activity in human data, we used the scRNA-seq dataset published in Carpenter*, Elhossiny*, Kadiyala* *et* al, 2023^30^. We split the data into acinar or epithelial cell subsets, as determined by cell type annotation. To calculate transcription factor activity score for TP53, we used run_viper() function in decoupleR R Package (v2.6.0) using default parameters.

###### Signature-based annotation of epithelial subpopulations

For signatures derived from mouse datasets, we converted mouse symbols to their corresponding human orthologs using convert_mouse_to_human_symbols() function from nichenetr R package (v2.0.1). We used AUCell R Package (v1.22.0)^31^ to score the gene sets of interest in each cell using default parameters except for aucMaxRank which was set to include 10% of the number of genes in the rankings. We decided to use this method for scoring gene signatures based on our prior experience with this data and the fact that AUCell is not sensitive to the scale factor used during normalization or the inclusion/exclusion of specific cell populations.

#### SPATIAL DATA PROCESSING AND ANALYSIS

##### Image processing and quantification

Our image collection and analysis aimed to quantify (1) signaling proteins in progenitor-like or other premalignant cells (i.e., P53 and phospho-ERK (p-ERK)), and (2) progenitor-like epithelial cells in response to perturbing the premalignant pancreas (i.e., HMGA2- or VIM-positive cells). For the latter, we collected whole tissue scans, reasoning that this strategy would most faithfully quantify the progenitor-like cell population, by minimizing bias and variability stemming from spatial heterogeneity in progenitor-like lesions (documented in Fig. S12c). To quantify how premalignant states differ in P53 abundance, we selected FOVs containing lesions rich in progenitor-like cells (MSN+) and spatially adjacent lesions devoid of this state (MSN−), providing an internally controlled setup for comparison. All analyzed tissues were collected at 20X magnification with a pixel size of 0.34 μm px^−1^.

To process and analyze whole tissue scans, we divided images in non-overlapping FOVs of 500 × 500 px for *p53* perturbation, HMGA2 and VIM datasets, or 1000 × 1000 px for KRAS inhibitor datasets. We used smaller FOVs for the *p53* perturbation cohort to minimize the inclusion of large empty regions in images derived from small fractions of the pancreas.

###### Generation of nuclear and whole-cell segmentation masks

To quantify HMGA2-positive cell abundance, P53 protein and p-ERK intensity, we used nuclear segmentation masks generated with Mesmer, implemented in the deepcell python package (v 0.12.9)^32^. This deep learning method was pretrained with a large number of manually annotated cell and nuclear masks from diverse imaging modalities and biological sources, aimed at generalizing mask prediction in new data, and it uses boundary predictions with a watershed approach to refine segmentation masks. For nuclear segmentation, we used the standard rolling ball background subtraction approach (*radius* = 10 px) on DAPI images, which we then fed to Mesmer with *compartment* = 'nuclear', *preprocess_kwargs* = {*'percentile'*: 99.9, *'threshold'*: True, *'normalize'*: True, *'kernel_size'*: 10}, and postprocess_kwargs_nuc = {*'maxima_threshold'*: 0.001, *'maxima_smooth'*: 1, *'interior_threshold'*: 0.1, *'interior_smooth'*: 0, other parameters as default}. Non-default parameters were manually tuned to recover accurate nuclear segmentation results in our datasets.

As VIM localizes outside the nucleus, we undertook whole-cell segmentation using Cellpose (v3.0.6)^33^ with DAPI (nucleus) and E-cadherin (membrane) stains to quantify VIM+ epithelial cells. Visual inspection revealed that Cellpose generated better whole-cell segmentation masks than Mesmer in our data. Like Mesmer, Cellpose is a deep learning method that is pretrained on a large collection of ground-truth masks; it leverages both cytoplasmic and nuclear stains to predict cell boundaries. Although we were interested in cells with mesenchymal properties, we noticed that progenitor-like cells with upregulated VIM expression retain membrane-localized E-cadherin. This suggests that our cells of interest lie at an intermediate point in the epithelial-to-mesenchymal spectrum, and enables the use of an epithelial marker such as E-cadherin to generate masks. Following background subtraction with the rolling ball approach (*radius* = 10) on DAPI and E-cadherin channels, we normalized images (saturating at the 99th quantile of the intensity histogram), and blended these channels into an RGB image. Next, we applied the pretrained segmentation model (*model_type* = 'cyto3') to generate segmentation masks using the model.eval function of the cellpose package with *diameter* = None, *channel_axis* = 0, *normalize* = True, *channels* = [2,3], *flow_threshold* = 0, and *cellprob_threshold* = 0.

Visual inspection of both Mesmer and Cellpose masks confirmed that these approaches better adapt to heterogeneous cellular densities and signal intensities in the tissue, compared to traditional approaches that rely on thresholding, morphological operations and watershed-based segmentation.

###### Signal quantification and quality control

We used segmentation masks to quantify fluorescent signal intensity, applying standard rolling ball background subtraction for markers with subcellular and stereotyped localization (e.g., HMGA2, VIM and P53). We avoided this background subtraction for p-ERK and TNC, as a single radius may fail to adequately capture the diverse sizes and geometries of epithelial and stromal structures that they mark; instead, we measured raw intensities at this step, and leveraged the full distribution of measured states to conduct background subtraction at the whole-slide or single-FOV level (see below).

In addition to measuring fluorescence intensity in the segmentation mask, we quantified geometric parameters such as area and solidity (a measure of roundness, calculated as the fraction of the convex hull of each segmented object covered by the actual mask). Geometric parameters are particularly helpful for QC, as they provide information about putative segmentation errors. They were computed using the regionprops function of the measure module in the python scikit-image package (v0.22.0). The resulting multidimensional representation of each cell in the dataset consisted of both intensity and geometric measurements, allowing downstream QC and *in silico* isolation and quantification of specific cell subpopulations.

Geometric features can be used to identify segmentation errors; for example, pairs of adjacent cells that segmentation fails to separate tend to have increased area, and decreased solidity. We used morphological parameters to identify putative low quality cells, filtering out cells with low solidity (< 0.8) or high nuclear area (>750 px), which excluded an average of 3% of cells in the data. **Data S4** summarizes results from QC filtering in whole-slide scan datasets.

###### Identification of epithelial cells

In our mouse models, RFP and GFP are activated for lineage tracing upon Cre-mediated recombination and doxycycline induction of shRNA alleles, thus serving as proxies for oncogenic KRAS activation (see Mouse model genetics). To identify premalignant epithelial cells, we computed the distribution of log-transformed (pseudocount = 1) average nuclear GFP or RFP intensity. Log-transformation of intensity values compresses the tails of the distributions, making bimodality more apparent and facilitating the identification of thresholds to isolate marker-positive cells. We used otsu thresholding on the log-transformed intensity to binarize the signal, resulting in the identification of premalignant epithelial cells (29% of cells, on average, across datasets). **Data S4** summarizes results of epithelial cell identification in whole-slide scan datasets.

###### Quantification of HMGA2- and VIM-positive cells

We used HMGA2 and VIM expression to quantify the abundance of progenitor-like premalignant cells in premalignant tissues. Similar to our treatment of GFP and RFP signals, we log-transformed (pseudocount = 1) the intensity distributions of HMGA2 and VIM followed by signal binarization using the Otsu thresholding method, capturing the second mode of the distribution. We summarized HMGA2 and VIM measurements as the average number of positive epithelial cells per field of view in each slide, and tested for differences between experimental conditions (KC^shp53^ and KC^shCtrl^) with a Two-Tailed Wilcoxon Rank Sums Test. **Data S4** provides detailed information regarding sample metadata and source data shown in Fig. 6b, Fig. 7b and Fig. 7e.

###### Quantification of p-ERK levels

We assessed the effectiveness of oncogenic KRAS inhibition *in vivo* through staining and quantification of p-ERK, a canonical downstream effector on MAPK signaling. To do so, we calculated the average p-ERK signal and non-epithelial cells per tissue. We used the average p-ERK intensity in non-epithelial cells as our estimate of the background signal, subtracting it from our measurements of p-ERK intensity in epithelial cells. The use of non-epithelial cells to estimate a global background per sample is motivated by selective p-ERK upregulation in the *Kras*^G12D^+ cells; on the other hand, non-cell autonomous effects leading to upregulation of MAPK signaling in non-epithelial cells would lead to underestimation of signal intensity in the epithelium, and thus our results should be interpreted as a lower bound estimate of the difference in p-ERK engagement between vehicle and MRTX1133-treated samples. We tested for differences between experimental conditions using a Two-Tailed Wilcoxon Rank Sums Test. **Data S4** provides detailed information regarding sample metadata and source data shown in Fig. S9a.

###### Quantification of P53 levels

Our single-cell data revealed the selective upregulation of p53 target genes in progenitor-like premalignant cells during both spontaneous and injury-induced tumorigenesis (Fig. 2a,b,7d and Fig. S11c,d). Protein stability is a key mechanism by which p53 is regulated^34^; thus, we quantified protein levels in premalignant cells, staining for P53 and the progenitor-like marker MSN separately in serial sections to avoid antibody isotype incompatibility. We first identified progenitor lesions (MSN+) in one section, considering only epithelial structures and remaining blind to p53 signal, and manually annotated the equivalent region in the adjacent section. Next, we quantified p53 abundance in epithelial cells from all MSN+ or all MSN− lesions. As p53 staining has a lower signal-to-noise ratio than cell-state markers such as HMGA2, MSN, VIM and GFP, we conducted local signal normalization to control for systematic intensity bias across the tissue. Specifically, for each FOV, we normalized nuclear p53 intensity in epithelial cells by its average intensity in non-epithelial cells, then computed the average normalized signal in MSN+ or MSN− epithelial cells for each tissue sample. We used a two-tailed Wilcoxon rank sums test to identify significant differences in P53 levels between MSN+ and MSN− cells. **Data S4** provides detailed sample metadata and source data information for Fig. S4b.

###### Quantification of TNC levels

Acute oncogenic KRAS inhibition led to collapse of the progenitor niche within 48 h of treatment, as evidenced by depletion of microenvironmental transcriptional states associated with progenitor-like cells (Fig. 6c,d). To test whether remodeling events were reflected at the protein level at these early time points, we quantified TNC, an injury- and cancer-associated ECM component that is upregulated in activated myofibroblasts in the progenitor niche. We opted for segmentation-free pixel quantification in the entire tissue section, given that TNC generates fibers not strictly associated with cellular or nuclear segmentation masks. Furthermore, we normalized by GFP-positive signal, as it represents the area of pancreatic parenchyma in the image. To identify positive and negative pixels, we analyzed intensity distributions of TNC and GFP per tissue section. First, we set a hard threshold of ln(pixel intensity) > 5.5, empirically determined to avoid empty areas in the image for both markers. Next, we computed the histograms of log-transformed TNC and GFP intensity values. We fit a Gaussian centered on the first mode of the intensity distributions using scipy.optimize to determine a background distribution. Using the mean and standard deviations of our background estimates, we standardized the intensity distributions, effectively equalizing the background distribution in all tissue slides. Lasly, we used triangle thresholding to identify pixels positive for TNC or GFP. We normalized the fraction of TNC-positive pixels by the fraction of GFP-positive pixels, and tested for differences between conditions using a two-tailed Wilcoxon rank sums test. **Data S4** provides details of sample metadata and source data in Fig. 6e.

##### Probe design for multiplexed smFISH

We built upon published software^15^ to design custom panels for multiplexed smFISH. This design strategy relies on pre-computation of all possible 30mer sequences found in mouse cDNAs (Ensembl GRCm38.p6), augmented with coding sequences of fluorescent proteins engineered into our mouse model. We excluded pseudogenes from the potential pool of mRNAs used for probe design. We compute multiple scores for each 30mer, including Tm, GC content, and potential for hybridization with rRNAs and tRNAs. We used the following parameters to include a 30mer into our candidate probe-set: GC-content (43–63%), Tm (66–76°C), excluding 30mers that contain at least a 15mer present in an rRNA or tRNA.

In addition, we computed expression-informed penalties to estimate the specificity of each candidate probe. We adapted published software to include single-cell information into the estimation of specificity scores, reasoning that it would decrease the chances of selecting probes with off-target binding to highly-expressed genes in rare cell populations. To do so, we considered our single-cell data from epithelial and immune compartments of the injured pancreas. Furthermore, we leveraged a published single-cell time course of *Kras*-driven transformation in pancreatic tissue to incorporate information about fibroblast, pericyte and endothelial gene expression^35^. Since our spatial analysis is focused on the premalignant-stage of pancreatic tumorigenesis, we excluded cancer-associated samples for the purpose of computing specificity scores.

To summarize single-cell gene expression as a function of cell state in distinct cellular compartments (epithelial, immune, fibroblast, pericyte and endothelial), we used our SEACells algorithm (v0.2.0)^36^ for aggregating cells into statistically equivalent cell states, known as metacells. SEACells metacells improve the robustness of analyses by overcoming sparsity in single-cell data, while still capturing its full heterogeneity, including rare cell states. For each cellular compartment, we first used standard log library-size normalization and dimensionality reduction using PCA (*n_pcs* = 100) for preprocessing. We then ran SEACells using *n_waypoint_eigs* = 10 (default) and *waypoint_proportion* = 0.9 (default). We selected the number of metacells per compartment such that the median number of cells per metacell ranged within a similar range:

- Epithelial: 300 metacells, median size = 79 individual cells
- Immune: 150 metacells, median size = 69.5 individual cells
- Fibroblasts: 100 metacells, median size = 88 individual cells
- Pericytes: 15 metacells, median size = 63 individual cells
- Endothelial: 50 metacells, median size = 85.5 individual cells

Next, we computed a summarized gene expression matrix *X* of dimensions *n* × *m*, where *n* is the number of metacells across all cellular compartments, and *m* is the number of genes in the dataset. In this matrix, *x*_ij_ is the average normalized linear counts of gene *j* across individual cells in metacell *i*. We normalized *X* by total counts per metacell, and scaled by an arbitrary factor of 2000. Lastly, we identified the maximum expression per gene across all metacells, and used these to compute specificity penalties during probe design. This strategy penalizes off-target binding to highly expressed genes, even when such high expression occurs in rare cell subpopulations.

We computed transcription-wide specificity as published^15^, with the exception that we assumed all isoforms of a gene contribute uniformly to its total expression (our single-cell data lack isoform-specific information). To compute the specificity score, each 30mer was represented as a collection of overlapping 17mer sequences in sliding windows with 1-bp shift. For each 17mer, we calculated the fraction of occurrences in the on-target gene (any isoform) out of all occurrences in the transcriptome, weighted by gene expression to penalize off-target binding to highly-expressed genes. We computed a final specificity score ranging from 0 (no occurrence from on-target gene) to 1 (all occurrences from on-target gene) for each 30mer by averaging the scores of its constituent 17mers, and selected 30mers with scores above 0.75 as candidates for our panels.

Candidate 30mers were used to compile primary probes for a set of query genes. We selected Ensembl canonical isoforms to design probes targeting a particular gene, aiming for 92 non-overlapping probes per gene. Whenever this was not possible due to transcript length, homology to other genes, or other sequence properties, we allowed a maximum overlap of 20 bp between probes. The use of overlapping probes was previously reported to maximize smFISH signal^37^ due to the probabilistic nature of probe–mRNA binding. Lastly, we appended readout sequences to each probe, which serve as recognition sequences for fluorescently labeled readout probes. In the case of genes for which we were not able to generate at least 75 probes, we added two or four copies of the selected readout sequence in order to amplify the fluorescent signal coming from such probes. Sequences of probes used in this study are included in Data S2 and Table S2.

##### Custom probe set design for spatial transcriptomics

Spatially resolved transcriptomics provides the opportunity to localize the rich transcriptional heterogeneity uncovered by single-cell experiments in tissue context. In the premalignant pancreatic epithelium, this includes transcriptional gradients that connect cellular states and key signaling axes such as *Kras* and *p53*. The premalignant epithelium undergoes dramatic microenvironmental remodeling, including the formation of fibrotic and inflammatory niches rich in myeloid cells. We sought to understand (1) the interplay between the adoption of distinct pancreatic epithelial states and changes in the microenvironment, (2) the spatiotemporal dynamics of niche transitions, and (3) what intercellular signaling circuits may mediate the formation and stabilization of progenitor niches. Thus, we designed a custom Xenium probe panel targeting 480 genes (**Table S4**) to capture:

1. Transcriptional heterogeneity in major cellular compartments, including cluster-level heterogeneity and transcriptional gradients connecting disparate cellular states.
2. Activation of major signaling programs (e.g., *p53*, *Kras*, *Yap*, interferon) operating in the benign-to-malignant transition in pancreatic cancer.
3. Genes that may construct intercellular circuits through juxtacrine and paracrine interactions.

###### Reference single-cell datasets for spatial expression profiling

Marker selection for imaging-based spatial transcriptomics should consider gene expression estimates for all cell populations in the target tissue. Optical crowding from abundant mRNA species not only hampers their accurate quantification, but also that of other mRNAs in the vicinity, due to the combinatorial encoding of mRNA identity through sequential imaging^14^. To inform our custom Xenium probe set, we leveraged the premalignant pancreas expression atlas we compiled for smFISH probe design (see Probe design for multiplexed smFISH), which includes epithelial and immune cell profiles in response to injury (with or without *p53* perturbation) as well as fibroblast, endothelial and mural cell data (excluding tumor-stage samples to better match premalignant cell stated distributions) from Schlesinger, Yosefov-Levi, Kolodkin-Gal *et al*.^35^.

Our compiled dataset also leveraged SEACells to generate more robust estimates of gene expression distributions across cell states, including rare cell states (v0.2.0)^36^. We reasoned that information from rare cell states would help us to retain biologically important mRNAs expressed in a small fraction of cells, and to exclude abundant mRNAs prone to optical crowding in rare cells and adjacent cells. To this end, we used the maximum expression per gene across all SEACells to guide marker selection for imaging-based spatial transcriptomics.

###### Gene expression constraints on marker selection

We avoided selecting mRNAs that lacked discrete foci due to optical crowding in smFISH staining of the premalignant pancreas (Fig. S5a,b; e.g. *Cpa1* in acinar cells, *Muc6* in gastric chief-like premalignant cells, *Tff1* in gastric pit-like premalignant cells or *Acta2* in activated stroma, *Fn1* in progenitor-like cells). Moreover, we identified ideal expression thresholds based on genes that we successfully imaged using multiplex smFISH (e.g. *Ptprc* in immune cells, *Adgre1* in macrophages, *Msn* in progenitor-like cells, *Anxa10* in gastric pit-like cells). Guided by experience with individual markers, we set the lower bound (max expression across metacells > 0.5), upper bound for subpopulation markers identified through unbiased clustering (max < 2.5), upper bound for gene-set derived markers such as communication genes (max < 4) and upper bound for biologically curated markers (max < 10), though we did make exceptions for certain markers of biological interest (e.g., expression of type I interferons, *Pecam1* endothelial marker).

###### Marker selection for cellular compartments and microenvironmental subpopulations

We used a combination of prior knowledge and unbiased marker gene identification, guided by expression constraints as described above. For example, we selected well-recognized markers of epithelial (*Cdh1*), immune (*Ptprc*), macrophage (*Adgre1*, *Csf1r*), fibroblast (*Pdgfra*, *Col5a1, Vim*), endothelial (*Pecam1*) and pericyte (*Des*, *Pdgfrb*) cells. Although excluded from our premalignant atlas, we included markers for a rare subpopulation of glial cells (*Fabp7*, *Plp1*) identified by Schlesinger, Yosefov-Levi, Kolodkin-Gal, *et al*.^35^, as well as *Fabp4*, an adipocyte marker.

To dissect heterogeneity within cellular compartments, we computed differential gene expression to identify markers of manually curated cell subpopulations (immune dataset^10^) or PhenoGraph clusters (*k* = 30) (fibroblast, endothelial and mural cells). We prioritized known markers of subpopulations (e.g. *Tnc*, *Dpt* or *Gli1* in fibroblasts) or communication pathways (e.g., *Csf1* in Gr-MDCs, *Lifr* in fibroblasts, *Kitl* in endothelial cells or *Csf2rb* in mural cells), as well as genes that independently mark subpopulations in distinct compartments (e.g. *Prox1* as a marker of normal duct cells and lymphatic endothelial cells). Collectively, this marker selection strategy allowed us to identify subpopulations across multiple compartments, while maintaining the biological interpretability of gene expression patterns in our spatial data.

###### Marker selection for premalignant epithelial cells

We aimed to capture the major premalignant states that we identified in dissociated single-cell data, including the cluster of progenitor-like cells with mesenchymal phenotypes that accumulate upon *p53* loss. We started with markers that have been reproducibly identified in independent single-cell datasets, including acinar (*Rbpj1*, *Nr5a2*), tuft cell (*Pou2f3*, *Dclk1*), neuroendocrine (e.g. *Syp*, *Ascl1*), gastric-like (*Elf3*, *Onecut2*)^35^, and progenitor-like (e.g., *Msn, Hmga2, Nes*) cell-state markers. We next used differential expression in PhenoGraph clusters to select markers that capture heterogeneity in gastric and progenitor-like states; for example, distinct subpopulations of gastric-like cells that correspond to chief-like (*F5*) or pit-like (*Anxa10*) states. In addition, we included *Cp* and *Rgs5*, two genes expressed in premalignant cells most resembling ducts. Lastly, we included a number of genes that transiently increased at the boundary of gastric-like and progenitor-like states (e.g. *Msln*, *F3*), as well as genes that progressively increased in expression along the progenitor axis (e.g. *Zeb2*, *Grem1* and *Piezo2*). We made the conservative decision not to probe for mRNAs of fluorescent proteins to distinguish premalignant and normal cells in our samples, due to the potential of these constitutively expressed mRNAs to hamper decoding the rest of the panel. Despite this, we note that we were able to identify structural and molecular features corresponding to normal ducts and islets, allowing us to focus on lesions consistent with premalignant phenotypes.

###### Marker selection for signaling pathways and biological processes

We manually selected markers for major signaling pathways, prioritizing negative feedback genes known to constitute some of the earliest transcriptional responses to pathway activation. Specifically, we probed for *p53* (e.g., *Mdm2, Cdkn1a, Bax*), MAPK (e.g., *Dusp4, Dusp6, Dusp5, Spry1, Spry2*), interferon (e.g., *Socs1, Socs3, Irf7, Oasl1*), YAP (*Ccn1, Ccn2*) and TGF-β (e.g., *Smad7, Il11, Has2*) signaling. In addition, we probed for cell cycle regulators, including major cyclins and cyclin-dependent kinases (CDKs) (e.g., *Ccnd1, Ccne1, Ccna1, Cdk1, Cdk2, Cdk4, Cdk6*) as well as CDK inhibitors (e.g., *Cdkn1b, Cdkn1c, Cdkn2a, Cdkn2b*).

###### Selection of genes involved in cell-cell interactions

To prioritize genes involved in cell-cell communication, we leveraged our previously identified communication modules—groups of ligands and receptors that are selectively upregulated upon *Kras* signaling in the premalignant pancreas and are predicted to form multiple interactions between cell states^10^. These communication modules involve a plethora of cytokines and receptors with known and predicted roles in tissue remodeling during tumor initiation (e.g., *Il33, Il18, Il1a, Ccl2, Csf2, Lif, Vegfa, Fgf*) and their cognate receptors. In addition, we probed for ligands and receptors from *Wnt*, *Shh*, *Bmp* and *Notch* signaling, due to their roles in development and tissue repair. Lastly, we included genes involved in cell adhesion (e.g., claudins, cadherins, integrins) and juxtacrine signaling (e.g., ephrin and semaphorin signaling), prioritizing genes based on gene expression constraints and HVG status (*n* = 3000) within each cellular compartment.

###### Computational design of probe set

We used 10x Xenium Designer (10x Genomics) with vendor assistance to generate isoform-specific probes for *Cdkn2a*. We used three independent reference datasets to compute cell-type utilization scores, which inform the potential for optical crowding as a function of target mRNA abundance in specific cellular states. Specifically, we used in-house single-cell data for premalignant epithelial cells after injury with or without *p53* knockdown (this work), immune cells in the injured premalignant pancreas^10^ and a PDAC progression atlas that includes all cellular compartments^35^. Given that these datasets were generated using different Ensembl annotation versions, we mapped *gene_symbols* to *ensembl_ids* to match those of the Ensembl build Mus_musculus.GRCm38p6.102. We excluded any gene above the recommended cell utilization score recommended by 10x Xenium Designer.

##### Spatial transcriptomics embedding and annotation

In our spatial data, each cell is represented by three primary features: (1) a transcript count matrix, (2) an x-y spatial coordinate (in μm), and (3) a polygon describing its segmented nucleus. In addition, we derive secondary features describing the morphological and molecular properties of the local niche of a cell, such as the structure and size of premalignant lesions, and the average gene expression of a given cell type in the vicinity of a given cell. These multiple viewpoints allow us to identify molecular and morphological correlates between the intrinsic state of a cell and its microenvironment. Our data is composed of 33 tissue samples, spanning 9 conditions, including different collection timepoints in the response to acute pancreatitis, as well as pharmacological inhibition of oncogenic KRAS, and *p53* knockdown in premalignant cells. In total, this comprises 9,463,399 cells (excluding cells with mixed phenotype), with an average of 92.83 mRNA counts per cell, and 57.92 detected genes in each cell. Table S5 shows the details of the samples included in our Xenium data.

In this section, we first describe our strategy to compute cell-level and niche-level representations from single-cell-resolved spatial transcriptomics data. Then we describe how we integrate these different viewpoints to study the dynamic interplay between premalignant epithelial cells and their microenvironment.

###### Transcript-to-cell assignments

Assigning transcripts to cells is a critical first step in recovering comprehensive and accurate cell states from imaging-based spatial transcriptomics data. Because cell boundary estimates based on pure geometric constraints, such as nuclear segmentation expansion followed by Voronoid tessellation (default 10x Xenium processing), result in pervasive transcript cross-contamination from adjacent cells, we opted to construct a count table composed only of transcripts that overlap nucleus masks (10x nucleus transcripts). This approach successfully eliminated inter-cell transcript contamination, as evidenced by our recovery of Leiden clusters with reasonable cell type purity based on marker expression (Fig. S5). However, it also resulted in an average loss of 57% of transcripts per slide (interquartile range 53%–60%). Although the limited sensitivity of this conservative approach is not ideal for all contexts, it was sufficient to recover expected cell-state heterogeneity in every cellular compartment (e.g., myofibroblast, myeloid, lymphoid and endothelial subpopulations) in our data (Fig. S5), including transcriptional gradients, such as the gastric–progenitor continuum in the premalignant epithelium (Fig. 3e,f). This is likely due to our use of probes targeting highly expressed marker genes in our panel, and to the fact that we focused on cell types that were not exceptionally small and therefore contained relatively high transcript counts.

###### Gene filtering, cell filtering and normalization

We excluded negative control probes from library size estimation and dimensionality reduction steps, but left them in the data for possible use in subsequent processing steps. Furthermore, we excluded cells with fewer than 25 mRNA counts. On average, we excluded 9.5% of cells per slide (interquartile range 7–10%).

For normalization, we estimated size factors per cell as the total 10x nucleus counts per cell. We normalized count matrices by dividing by size factors, and scaling by the median size factor in the data. We used linear, as opposed to log transformed counts, as this led to faster and reproducible convergence during the computation of UMAP embeddings. Linear counts may be more appropriate for this data modality given that our panel is enriched in communication genes, signaling proteins and transcriptional regulators, gene classes that tend to have lower expression than the abundant house-keeping genes in transcriptome-wide datasets.

###### Single-cell embeddings for spatial transcriptomics

We computed an initial single-cell embedding using concatenated count matrices from all tissue slices. To compute this embedding, we used GPU-based implementations of PCA, kNN graph construction and UMAP functions in rapidsai (v24.2.0). We conducted dimensionality reduction using PCA on linear normalized counts, keeping the top 136 PCs that captured 75% of variance. In addition, we noted that the use of more than 100 PCs was important to isolate molecularly and spatially distinct subpopulations that were nonetheless rare in the dataset (i.e., adipose, glial and non-pancreatic epithelial cells). Use of a smaller number of PCs collapsed these subpopulations into stromal and epithelial compartments, hindering the purity of our initial cell type annotation. We constructed a kNN graph using the GPU-based nearest-neighbor graph implementation of cuML python package (v24.02.00). We computed the kNN graph using euclidean distance on PC space (*k* = 30) as our metric, followed by UMAP for visualization (*n_neighbors* = 10), and computation of clusters using GPU implementations of leiden (*resolution* = 1.0). We used this integrated embedding to annotate coarse and refined cell states (see below). These annotations were transferred into any condition-specific embedding to maintain consistency in downstream analyses.

###### Cell type annotation

We first annotated major cellular compartments, then determined more refined cell states (details below). We leveraged spatial patterning to inform cell type annotation, for example, by identifying cellular states associated with lymph nodes, the edge of the tissue (e.g., mesothelial) or the pancreatic parenchyma.

####### Major cellular compartments.

To identify major cellular compartments, we annotated clusters based on known markers, then refined cell states within cell types by re-embedding and clustering cellular subsets. Specifically, we first computed clusters using the Leiden algorithm on a kNN graph (*k* = 30) constructed on PC space, with *resolution* = 1.0. We manually assigned clusters to major compartments based on markers in our panel: epithelial (*Cdh1, Itgb4, Cpa1, Onecut2, Prox1, Cp, Krt7*), endothelial (*Pecam1*), immune myeloid (*Ptprc, Adgre1, Csf1r, Csf3r*, *Cd68, Itgam, Itgax*), immune lymphoid (*Ptprc, Cd3g, Cd4, Cd8a, Foxp3, Cd19, Cd79a, Lman1, Gata3, Rora, Trdc*), fibroblast (*Col5a1, Pdgfra, Dpt, Pdpn*), mural cell (*Des, Rgs5, Tagln, Fhl1*), adipose (*Fabp4*), mesothelial (*Msln*) and glial/nerve (*Plp1, Fabp7, Ncam1, Ncam2, Syp, Ngfr*).

####### Refined cellular states and condition-specific embeddings.

To identify more granular cell states, we re-embedded cells and recomputed clusters within each compartment, then annotated clusters based on the expression of marker genes from the literature, manually curated single-cell atlases of the premalignant pancreas, or unbiased identification of cluster marker genes using scanpy’s sc.tl.rank_genes_groups. The latter approach highlighted genes with higher expression in a cluster relative to all other clusters; among these, we selected markers that would provide the most interpretable cell-state label (e.g., *Cd55* in endothelial cells or *Ccn2* in myofibroblasts). In addition, we computed condition- and subpopulation-specific embeddings for downstream analyses. For example, Figs. 3–5 focus on variation in epithelial cells and their niches in unperturbed samples to gain insights into tissue remodeling around progenitor-like cells.

To compute refined single-cell embeddings, we first excluded negative control probes and genes expressed in fewer than 1% of cells within a compartment, so that they would not influence normalization and dimensionality reduction. Next, we normalized the data by dividing counts by library size in each cell and re-scaling by the median library size, applied PCA on normalized linear counts, and kept the top PCs that captured 75% of total variance. We constructed a kNN graph on Euclidean distance in PC space (*k* = 30) and visualized our embeddings using UMAP (*n_neighbors* = 10, *min_dist* = 0.1).

Next, we computed clusters at different resolutions to dissect heterogeneity within cell types. We used Leiden clustering on the kNN graph with *resolution* = 1.0 for initial cluster annotation, then computed PhenoGraph clusters on the kNN graph by leveraging GPU implementations of Jaccard similarity matrix construction and Louvain clustering at multiple resolutions (*resolution* = 1.0, 0.75, 0.5) in the cugraph module of rapidsai. The use of multiple resolutions allowed us to distinguish closely related cell states during cell type refinement, while avoiding noise from over-clustering.

See Table S6 for all cell-type or condition-specific embeddings in our study; methods for annotating specific cell states are presented below.

####### Epithelial states.

We first annotated Leiden clusters based on major premalignant subpopulation markers identified during PDAC initiation (^10,35^ and this work): progenitor1 (*Msn, Hmga2, Itgb4*), progenitor2 (*Vim, Piezo2, Tnc*), gastric-like (*Elf3, Onecut2, Lgals4*), gastric chief-like (*F5*), gastric pit-like (*Anxa10*), tuft (*Pou2f3, Dclk1*), neuroendocrine (*Syp, Hepacam2*), duct-like (*Cp, Prox1, Rgs5*), ADM (*Rbpjl*), and cycling cells (Mki67).

To distinguish normal ducts from premalignant cells with a duct-like phenotype, we re-embedded the duct subpopulation using the above strategy. We identified clusters corresponding to normal ducts based on *Rgs5* and *Prox1* expression^35^. Cells in this cluster formed morphological structures characteristic of normal duct, providing support for our annotation.

####### Fibroblast states.

We used fibroblast signatures from cancer contexts^38,39^, together with the spatial organization of stroma in the premalignant pancreas, to assign Leiden clusters:

1. myCAFs. Most fibroblasts in the premalignant pancreas (86%) resembled myofibroblast cancer-associated fibroblast (myCAF) states (*Sdc1, Tagln, Tgfb1, Tnc, Epha4, Igf2, Itgbl1*). These cells populated the pancreatic parenchyma, and were in close proximity to premalignant epithelial cells (Fig. S5). We identified marker genes expressed in myofibroblast subclusters using the scanpy function sc.tl.rank_genes_groups, and used these markers to label specific subpopulations (e.g., *Tnc+, Ccn2+, Gli1+, Igf2+*).
2. iCAFs. A group of cells were consistent with the inflammatory cancer associated fibroblast (iCAF) phenotype (*Cxcl1, Cxcl12, Tnxb*) and expressed the universal fibroblast marker *Dpt*^40^. These fibroblasts were excluded from the parenchyma (Fig. S5).
3. Trf+ CAFs. A small subset of fibroblasts (0.2%) expressed antigen-presenting CAF (apCAF) markers (*Trf, Sdc4*). We conservatively labeled these as Fibroblast_Trf given that our panel did not include additional apCAF markers.

####### Myeloid states.

We used our single-cell immune atlas of the premalignant pancreas^10^ to group myeloid cells into three broad categories, followed by cell-type refinement:

1. Monocyte/macrophages. Most myeloid cells in the premalignant pancreas (91%) resembled monocyte/macrophages (*Csf1r* and *Adgre1*). Spatially patterned clusters within this compartment guided the annotation of refined states. Specifically, *Cd163*+ cells localized outside the pancreatic parenchyma and *Nlrc5*+ cells localized to lymph nodes; *Maf* and *Itgax* characterized clusters at two extremes of the continuum of macrophage states; and a cluster of *Itgax*+, *Cd274* (PD-L1)^high^ cells appeared upon *p53* knockdown in the premalignant epithelium. Thus, granular myeloid subsets were characterized by spatial patterning in addition to transcriptional variation in the premalignant pancreas.
2. Granulocyte–myeloid derived cells (GrMDCs). A cluster defined by high *Csf3r* and low *Csf1r* expression characterized GrMDCs.
3. Dendritic cells. Dendritic cells were defined by *Itgax* (CD11c) expression, lack of *Csf1r* and *Adgre1*, and expression of dendritic cell markers in the panel (*Ifi205, Itgae, Jak2*).

####### Lymphoid states.

We used markers from our single-cell immune atlas of the premalignant pancreas^10^, along with cluster refinement and marker identification using the scanpy sc.tl.rank_genes_groups function to define lymphoid cells groups. We re-embedded non-B-cell-related lymphoid cells (see Refined cellular states and condition-specific embeddings), followed by PhenoGraph clustering and cell-state annotation to define the following populations: (1) B cells (*Cd79a, Cd19*) and plasma cells (*Lman1*), (2) parenchyma associated lymphoid cells, including Tregs (*Cd4, Foxp3*), Th17 cells (*Il23r*), gdT cells (*Trdc*), innate lymphoid cells (*Gata3*), NK cells (*Ncr1*) and mast cells (*Kit*), and (3) lymph-node-associated CD4 and CD8 T cells.

####### Endothelial and mural states.

Endothelial cells were broadly divided into vascular (*Pecam1+/Prox1−*) or lymphatic (*Pecam1+/Prox1+*) subpopulations. Within the vascular subpopulation, we labeled clusters based on known biology (e.g., activated endothelial cells expressing *Selp*) or marker expression (e.g., *Piezo2*+ endothelial cells). We annotated mural cell clusters based on markers identified using the scanpy sc.tl.rank_genes_groups function. Endothelial and mural cells exhibited their expected spatial colocalization, and clusters of these cellular compartments also showed spatial patterning related to vessel size (Fig. S5).

####### Mixed cell states.

Some clusters showed evidence of cross-contamination between cell types, particularly between fibroblasts and myeloid cells (e.g., expression of macrophage marker *Csf1r* with fibroblast-specific *Igf2*). These artifacts can emerge from errors in nuclear segmentation or from 2D projection of transcripts, both of which are expected to increase in tightly packed tissues such as the premalignant pancreas. We flagged clusters in these mixed states, kept them in the dataset for visualization of tissue architecture, but excluded them from biological analyses involving cell type proportions or changes in gene expression between niches. Altogether, mixed cell states encompassed 3.1% of our data, and were dominated by cells sharing fibroblast and myeloid profiles (1.7% of all cells). Table S7 summarizes the fraction of cells in our dataset corresponding to mixed states.

###### Compartment-aware gene censoring

Despite restricting our analyses to 10x nucleus transcripts and excluding clusters with mixed cell states, we observed some transcript contamination between cell types. For example, *Igf2* was expressed in myeloid cells, despite its absence in dissociated reference datasets^10^. As expected, these effects were larger for highly expressed markers of specific subpopulations or cellular compartments, which can result from projecting expression onto two dimensions from a 3D section (https://doi.org/10.1101/2025.03.14.643160, https://doi.org/10.1101/2025.01.20.634005), or from transcript diffusion.

To mitigate the effect of contaminants in downstream analyses, we leveraged dissociated reference datasets^10,35^ to identify the fraction of cells within each cellular compartment with non-zero counts for any given gene. For downstream analyses, we censored genes in each cellular compartment that were expressed in < 1% of cells in every reference dataset. This threshold was meant to exclude genes with little evidence for expression in single-cell data, suggesting that their presence in our spatial dataset could be due to cross contamination between cell types. On average, 303 (67%) of the genes included in our panel passed this filter.

To complement filtering based on summary statistics from dissociated cells, we identified robustly expressed genes based on the fraction of cells within a subpopulation of a cellular compartment that express a given gene (gene detection rate) in our spatial data. Assessing per-subpopulation rather than per-compartment statistics ensures that no positive populations are missed.

For a specific cell type, this resulted in a matrix *P*^cxm^ where *c* is the number of subpopulations within the cell type, and *m* is the number of genes. To identify a ‘robust expression’ threshold within a cell type, we flattened the matrix *P*, and computed the histogram of subpopulation-level gene detection rate (*n_bins* = 25). Next, we used triangle thresholding to identify a cutoff that distinguished genes with high or low detection rates. On average, this strategy nominated genes as robustly expressed if they were detected in at least 9% of cells within a compartment subpopulation. We applied it to all coarse cell types in our data to identify compartment-specific gene sets with evidence of robust expression. Across cell types, an average of 262 (54%) of genes were classified as robustly expressed. For a given cell type, an average of 240 (50%) of genes passed both the spatial and dissociated data detection thresholds. We used this high-confidence gene set to analyze within-cell-type spatial heterogeneity in expression (Figs. 5a,b, 7f and Figs. S7f, S8a,b,d). We note that for future work, newer methods can correct transcript misassignment resulting from contamination in spatial transcriptomics data (https://doi.org/10.1101/2025.01.20.634005).

Table S8 summarizes the results of compartment-aware gene censoring across all cellular compartments.

##### Spatial transcriptomics transcriptional and cell-state gradients

Our single-cell characterization revealed that premalignant epithelial phenotypes do not exist as discrete states. Rather, we and others observe pervasive continuity in the transcriptional heterogeneity of individual premalignant cells (Fig. 1e)^10,35^. These continuums are reflected by the presence of cells with mixed states (Fig. 1e), which suggest cellular plasticity. Embracing continuity in cell states allowed us to connect these features of plasticity at the single-cell level with tissue remodeling events encoded in spatial transcriptomics data. We took two complementary approaches to characterize transcriptional gradients in premalignant cells: (1) we used gene signatures of canonical premalignant subpopulations^10^ to visualize their continuous variation along an epithelial-specific phenotypic manifold and (2) we used diffusion component analysis to identify the major axes of transcriptional variation in epithelial states.

###### Visualization of gene expression signatures in epithelial cells

We asked whether epithelial cells in our spatial data recapitulated the spectrum of premalignant transcriptional states we observed in dissociated scRNA-seq. We used markers of premalignant subpopulations in our Xenium panel as transcriptional signatures (**Table S4**), and computed a signature score matrix X^nxz^ where *n* is the number of epithelial cells in non-perturbed samples (*n* = 1,388,199) and *z* is the number of epithelial states (*z* = 6, progenitor-like, gastric-like, duct-like, neuroendocrine, tuft, adm). To compute the signature score matrix, we first standardized our log-transformed count matrices (*pseudocount* = 1) by computing the *z*-score of each gene over all epithelial cells. The signature score *x*_i,j_ is the average standardized expression of genes in *signature*_j_ for *cell*_*i*_. We normalized the signature score matrix column-wise, such that signature scores ranged between [0,1], saturating the signature score at the 90th quantile. We visualized signatures by plotting epithelial cells in both spatial and transcriptional (UMAP) domains, pseudocoloring each cell by aggregating RGB color vectors weighted by each signature score (see Simultaneous visualization of multiple signatures). This visualization strategy showed that our spatial data captured the transcriptional heterogeneity in epithelial states in single-cell data (compare to Figs. 1e, 3e, 4b in ref. ^10^). Furthermore, the mixture of signatures at intermediate points connecting extreme cellular states highlighted continuity between subpopulations of premalignant states, such as the presence of epithelial cells with mixed gastric and progenitor signatures.

###### Diffusion component analysis in spatial data

To identify the major axes of continuous variation in premalignant states, we used diffusion maps, which model gradual cell-state transitions along the phenotypic manifold as a diffusion process in a kNN graph. Starting from a kNN graph (*k* = 30) constructed on a single-cell embedding for gastric-like or progenitor-like cells from unperturbed samples (see Refined cellular states and condition-specific embeddings), we constructed a diffusion operator, followed by eigendecomposition to identify diffusion components (Note S1). The first diffusion component, representing the dominant axis of transcriptional variation among gastric-like and progenitor-like cells, captured gradual changes in gene expression linking gastric-like epithelial states on one extreme, and progenitor-like on the other, including mixed phenotypes at intermediate points (Fig. S4d). This approach positioned each of the 838,581 gastric-like or progenitor-like cells in our data along a continuous axis of variation between these subpopulations.

##### Spatial transcriptomics niche analyses

###### Definition and identification of cellular niches

Spatial transcriptomics makes it possible to represent a cell not only by its intrinsic properties, but also by the properties of its cellular neighbors and surrounding tissue structure. The spatial niche framework provides a simple and elegant approach to connect a cell with its surroundings^41,42^. In general, a niche can be defined as a set of cells in shared physical space, represented by a spatial neighborhood graph *Gn* × *n*, where *n* is the number of cells in the data, and *G*_i,j_ is a measure of spatial distance between *cell*_i_ and *cell*_j_.

We used 2D Euclidean distance as our metric, and defined a niche as the set of cells within a radius *r* of a reference cell, which we refer to as the anchor cell. In using a fixed radius to define a niche, we can ensure biological length scales that are interpretable and represented by well-defined physical units; dissociated single-cell data, by contrast, does not report physical distance, with implications for our understanding of the spatial extent of cellular influences. We use a radius of 60 μm throughout this work as this length scale captured glandular structures in the premalignant pancreas, establishing individual lesion size as a unit of analysis. However, we recognize different choices of *r* are bound to highlight different emergent properties of intercellular communities, such as the formation of large spatial domains of progenitor-like cells upon *p53* knockdown (Fig. S11f).

We extracted three types of features from our spatially defined niches: (i) morphological parameters of premalignant lesions, (ii) cell composition vectors of cellular niches, and (iii) compartment-specific locally averaged niche expression matrices (detailed below). These features enabled us to characterize tissue remodeling events that are coupled to gradual changes in premalignant cell states.

###### Quantification of structural and morphological parameters

To study morphological changes associated with the adoption of distinct premalignant cell identities, we quantified three parameters of local tissue organization in individual cell niches: lesion size, epithelial fraction, and luminal area. Our computational approach relied on graph and pixel-based representations of epithelial structures, enabling tissue properties to be estimated from cell centroids alone.

####### Lesion size.

We defined a lesion as a contiguous set of physically adjacent epithelial cells in the tissue. We first built a spatial neighbor graph connecting all epithelial nuclei centroids within 20 μm. The use of this length scale, which is within the range of a cell diameter, maximized the probability that two cells are interacting physically in the tissue. Next, we identified connected graph components in our spatial neighbor graph. We defined epithelial lesion size as the number of cells in each connected component.

####### Epithelial fraction.

The epithelial fraction represented the fraction of epithelial cells out of all cells in a niche.

####### Luminal area.

To quantify luminal area, we adopted classical image processing procedures aimed at identifying holes in the tissue surrounded by epithelial cells. We identified (1) non-empty regions covered by tissue; (2) closed holes within the tissue; and (3) empty space that was not closed and may have resulted from epithelial disruption during tissue processing:

1. To identify empty space, we generated a tissue image starting from single-cell centroids. For each tissue slice, we constructed a 5 μm px^−1^ mesh grid covering all cellular centroids using numpy (v1.23.4) linspace and meshgrid functions. Next, we calculated the number of cell centroids that overlapped each xy coordinate of the mesh grid, resulting in a bitmap-based representation of the tissue, where values correspond to the cell density at each pixel. Using a 2D Gaussian filter with an isotropic kernel of 3-px bandwidth (15 μm), we denoised cell density estimates in the image. Lastly, we applied an empirically determined threshold of 0.01 to identify pixels with high cell density (foreground) from empty pixels (background).
2. To identify closed holes surrounded by epithelial cells, we computed a bitmap-based representation of epithelial cells. Starting from the mesh grid in step 1, we set any pixel overlapping with an epithelial centroid to 1, and all other pixels to 0. To close small gaps between pixels associated with a single glandular epithelial lesion, we dilated our binarized image of the premalignant epithelium using the dilate function in the cv2 package (v4.10.0) with an 10-px disk kernel and *n_iterations* = 1. Next, we filled holes in the image using the cv2 floodFill function, followed by erosion using the erode function to counterbalance the original dilation, returning the boundaries of epithelial lesions to their original scale. Luminal pixels correspond to empty points that lie within filled epithelial masks.
3. To identify empty luminal regions that are not enclosed, we used a pixel propagation procedure, whereby luminal pixels are identified as empty points within 20 μm of an anchor epithelial cell. We used this propagation strategy iteratively, such that luminal points identified in the first iteration lead to the identification of the next layer in the lumen. The use of a 20-μm threshold was empirically determined to prevent classification of the tissue edge as lumen due to the presence of epithelial cells close to the tissue edge.

We defined lumen area as the number of pixels classified as lumen within a radius *r* of a cell (*r* = 60 *μm*) for Fig. 3h.

####### Structural changes along the gastric–progenitor DC.

To quantify how structural features in the vicinity of premalignant cells change along the gastric–progenitor DC, we discretized this DC into 11 equally sized bins, and computed the distribution of niche morphological parameters as a function of an epithelial cell’s DC bin (Fig. 3g–j).

###### Quantification of cell-state proportions in niches

The distribution of discretized cellular states in niches provide one description of the compositional heterogeneity of cellular communities in the tissue. To quantify the distribution of cell states across niches, we constructed a cell-state count table, represented as a *n* × *m* matrix, where *n* is the number of niches (centered at every single cell) and *m* is the number of discretized cell states in the dataset. Then, we computed cell-state distributions at two resolutions, coarse cell-type and cellular compartment.

###### Compartment-specific niche expression matrices

The active molecular programs within a niche provide an additional description of the compositional and transcriptional heterogeneity of cellular communities. The first challenge in characterizing spatial patterns of transcriptional variation at the single-cell level is sparsity; each cell in our data expressed only ~60 transcripts on average (Table S5). Moreover, genes in positive cells (cells with at least one mRNA count for that gene) had a median of 1.3 raw counts (interquartile range, 1.2–1.6). Sparsity persisted even when restricting our analysis to genes with known robust expression in a specific cell state. For instance, *Msn* had a median of 2 raw counts (interquartile range of 1–3) in *Msn*-positive cells. This contrasts with smFISH measurements of the same gene, which average 55 transcripts per progenitor-like cell (Figs. 2b,3c and Fig. S5). Dropouts, or unobserved transcripts, dilute differences between cell subpopulations and limit the estimation of gene–gene covariance matrices^36^. We and others have developed approaches to overcome sparsity in dissociated data, including gene count imputation through signal sharing in kNN graphs^26^ and aggregation of gene counts in metacells—sets of cells that occupy the same transcriptional state, with minor residual variation between cells due to technical as opposed to biological sources^36,43^.

To overcome sparsity in Xenium spatial transcriptomics, we spatially aggregated mRNA counts, leveraging coarse cell-type annotations to obtain a compartment-specific summarized gene expression vector for each cellular niche. To do so, we first constructed a cell type x niche x gene count table, where each entry corresponded to the number of mRNA molecules in cells from a specific cell type in every niche in the dataset. We computed size factors as the total number of mRNA molecules in cells from the specified cell type in the niche, and used them to normalize our compartment-specific count matrix, followed by scaling by the median library size. Using this approach, each niche is represented by *m* independent count matrices, where *m* is the number of coarse cell types queried in the niche.

Spatial aggregation overcomes sparsity while preserving information about coarse cell types that express a given gene, facilitating the analysis of cell-type-specific expression differences between niches. On the other hand, aggregation loses single-cell resolution, and may mix heterogeneous states. However, our analyses at single-cell resolution showed concerted shifts in cell-state distributions within cell types when comparing niches dominated by gastric-like or progenitor-like cells (Fig. 4e), suggesting that our spatial aggregation approach is well suited to capture the average changes in gene expression that accompany such shifts. In addition, while heterogeneity in cell density can introduce artifacts in these compartment-specific count matrices, as denser neighborhoods are more likely to have fewer collective dropouts in gene expression, our choice of *r* = 60 *μm* mitigates this problem due to the large number of cells (> 100 on average) within each niche.

###### Molecular and compositional changes along the gastric–progenitor DC

Oncogenic KRAS activation leads to the transcriptional diversification of the pancreatic epithelium, as well as profound tissue remodeling events linked to inflammatory responses^44–46^. We hypothesized that the identity and local distribution of premalignant cells in tissue profoundly impact the morphological, cellular and molecular properties of their surrounding microenvironment. To investigate the relationship between cell-state changes in the premalignant epithelial cells and remodeling of the surrounding microenvironment, we integrated two viewpoints of our spatial data: (1) our quantification of niche features (see Definition and identification of cellular niches and subsequent sections) and (2) our characterization of gastric–progenitor cell-state continuum in premalignant cells (see Diffusion component analysis in spatial data). This strategy allowed us to connect the gradual transcriptional changes in premalignant cells with gradual changes in morphological, cellular and molecular properties of their niche.

####### Ordering cells and niches by average gastric–progenitor DC.

We noted that gastric and progenitor-like cells within the same niche were at similar positions in the gastric-progenitor DC axis, suggesting spatial coordination in the adoption of divergent premalignant cell identities. The spatial coupling of premalignant heterogeneity allowed us to average the gastric-progenitor DC value of gastric or progenitor-like cells in the niche, resulting in a niche analog to the gastric-progenitor DC axis that we defined at the single cell level. In order to minimize variation due to averaging of small numbers of cells, we restricted our analyses to niches anchored at any cell with at least 10 gastric or progenitor-like cells in their niche. This newly constructed axis positioned each of the 2,772,533 niches in our data along a niche continuum linking the canonical gastric or progenitor niches.

####### Summarization of niche features along the gastric–progenitor continuum.

We used three complementary approaches to quantify how the cell-state composition of niches changes as a function of variation along the average gastric–progenitor DC, which we discretized into 100 uniform bins.

For our first approach, we quantified cell-type proportions in niches as a function of the DC axis bin, by computing the median and interquartile range of the relative frequencies of coarse cell types (Fig. S6a), or cell states within a cell type (Fig. S6c). For cell states, we included niches harboring at least 10 cells of the specified coarse cell type in the niche.

For our second approach, we disregarded discrete cell-state labels and instead visualized changes in the distribution of transcriptional states within cellular niches as a function of the DC axis bin (Fig. 4e). Given niche matrix *Gr* × *n*, where *r* is the number of niches, *n* is the number of cells in the data, and *G*_*i*,*j*_ = 1 if *cell*_*j*_ belongs to *niche*_*i*_, otherwise 0, we:

1. Extracted cell-state composition vectors for niches in each gastric–progenitor bin. We identified all niches that fall in a given bin in *G*, and extracted all cells that appear in these niches. This set of cells represents the neighborhood composition vector of niches in a specified bin along the gastric–progenitor axis.
2. Visualized cell-state densities in UMAP representations. We projected 2D cell density estimates onto a UMAP of tumor microenvironmental cells (Fig. 4e bottom) (see Visualization of cell state density in two-dimensional representation). As a reference, we visualized the density of gastric- or progenitor-like epithelial cells in the bin (Fig. 4e top). This approach provided a label-free visualization of changes in niche cell-state distributions coupled to changes in epithelial anchor-cell states.

For our third approach, we quantified gene expression changes in specific cellular compartments as a function of the DC axis bin (Fig. 4f). Given compartment-specific niche expression matrix *X*^*c* × *n* × *m*^, where *c*, *n* and *m* represent the number of cell types, niches containing at least 10 gastric- or progenitor-like cells, and genes, respectively, and x_*s*,*i*,*j*_ is the average expression of *gene*_*j*_ in cells of *type*_*s*_ in *niche*_*i*_ (see Compartment-specific niche expression matrices), we:

1. Standardized compartment-specific niche expression matrices. We log-transformed the niche expression matrix (*pseudocount* = 1) to stabilize variance and reduce the impact of outliers in downstream quantification. We standardized *X* by *z*-scoring log-transformed niche expression over all niches, for every cell type and gene pair. The resulting matrix represents cell-type-specific heterogeneity in gene expression across niches.
2. Summarized gene expression along niche bins. For a given cell type, bin and gene, we averaged our standardized compartment-specific niche expression matrix over all niches in the bin, resulting in a summarized niche expression matrix *X*^*c* × *b* × *m*^, where *b* is the total number of bins along the DC axis (*b* = 100). The resulting matrix represents the continuous gene expression shifts along the gastric–progenitor niche continuum in each cell type, analogous to the quantification of gene trends in pseudotime analysis^25,47^.
3. Visualized gene trends. To examine trends in niche gene expression, we first sorted genes based on how early their expression changed along the gastric–progenitor DC axis—when it reached a maximum value, minimum value, or changed in average *z*-score sign. We chose to visualize markers of gastric-like (e.g., *Anxa10, F5, Onecut2, Elf3*) and progenitor-like (e.g., *Msn, Hmga2, Vim*) cells, as well as myeloid (e.g., *Maf, Mab, Itgax*) and fibroblast (e.g., *Tnxb, Dpt, Postn, Tnc*) subpopulations that changed in abundance along the DC axis. In addition, we visualized genes that suggest shifts in signaling along the niche axis (e.g., *Oasl2*, *Irf7* for interferon signaling; *Dusp4, Dusp6, Dusp5* for MAPK signaling; *Mdm2, Cdkn1a* for P53 signaling). Lastly, we selected genes related to communication and wound healing that were upregulated in distinct cellular compartments of the progenitor niche.

These three approaches reveal changes in cell-state frequencies, cell-state densities, and compartment-specific gene expression as niches progress from gastric to progenitor-like epithelial anchor states, providing complementary viewpoints on the spatiotemporal dynamics of tissue remodeling during progenitor niche formation.

###### Comparison of spatial niche expression with dissociated data

Our spatial analyses revealed a coupling between epithelial states and fibroblast and myeloid states within corresponding niches along the gastric–progenitor continuum (Fig. 4f). These results were based on a handful of markers in our Xenium panel. To gain insights into broader programs across the full transcriptome, we reasoned that scRNA-seq data from our premalignant samples (see **Analysis of premalignant tumor microenvironment data**) should reflect similar axes of continuous variation, and thus searched for these axes in non-epithelial cell types in the niche microenvironment.

In the fibroblast compartment, we used our myofibroblast cell embeddings, as these are the major stromal components of the pancreatic parenchyma (see Table S3 for embedding details). We applied diffusion component analysis to the scRNA-seq data (*k* = 30, adaptive Gaussian kernel with distance to tenth neighbor as bandwidth) to identify the major axis of transcriptional variation (DC1). We ordered cells along this axis and grouped them into 50 bins of equal cell number, then averaged *z*-scored gene expression over all cells in each bin to compute gene trends along DC1. Plotting genes in this orthogonal dataset (Fig. 4f) revealed concordant trends: progressive downregulation of genes associated with inflammatory fibroblasts and universal fibroblast state (e.g., *Tnxb, Dpt, Cxcl12*), induction of activated myofibroblast genes near the progenitor-like cell extreme (e.g., *Tnc, Tgfb1*), and upregulation of Shh-related signatures in the middle of the axis (e.g., *Gli1, Ptch1, Ptch2*). By leveraging transcriptome-wide information, we confirmed the induction of additional fibroblast activation markers at the end of DC1, including *Acta2, Timp1* and *Spp1*, as well as tumor suppressor *Cdkn2a*, recently highlighted as a marker of senescent myofibroblasts that promote pancreatic cancer progression^38^ (Fig. S7e).

In the myeloid compartment, we examined PhenoGraph clusters expressing *Maf* or *Itgax*, which each mark an extreme point in our niche continuum, and exist at intermediate levels along this axis in our spatial data. Notably, diffusion component analysis revealed that the *Maf*–*Itgax* dichotomy also captured the major axis of variation in scRNA-seq data (DC1). Projection of genes upregulated alongside *Itgax* and *Cd274*, markers of myeloid cells in the progenitor niche, revealed the engagement of a plethora of genes associated with immune suppressive and pro-fibrotic myeloid subsets (e.g., *Spp1, Arg1, Il1b, Fn1*). Taken together, this analysis allowed us to contextualize cell-state changes in the progenitor niche with transcription-wide information, some of which are known to mediate fibrotic responses and cancer progression (Fig. S7d).

###### Discretization of canonical gastric and progenitor niches

We found it useful to identify a single canonical progenitor and gastric-like niche, in order to simplify the computation of gene expression differences between these two communities (in wound-healing and communication gene programs, for example). We defined the canonical progenitor niche as the set of niches belonging to niche bins in which the median proportion of progenitor-like cells was higher than the median proportion of gastric-like cells. This corresponded to the top 22% of niches along the average gastric–progenitor DC niche axis. We defined the canonical gastric-like niche as the bottom 22% of niches ordered along the same axis. For simplicity, we term these sets gastric niche and progenitor niche.

###### Wound-healing signatures in the progenitor niche

We noticed that a plethora of genes with roles in wound healing processes (e.g., *Tnc* and *Postn* ECM remodeling, *Pdgfb* signaling, plasminogen processing) were upregulated in the progenitor niche across cellular compartments, motivating us to systematically test for wound-healing gene upregulation in our curated gene panel. We used all genes in our panel that appear in the Gene Ontology wound-healing response gene set (GO:0042060) as a wound healing signature, grouped niches by canonical gastric or progenitor label and by biological replicate, and averaged compartment-specific *z*-scored niche expression of these genes. Differences in signatures (Fig. 4g) or individual signature genes (Fig. S6f) between gastric and progenitor niches were tested using a two-tailed Wilcoxon rank sums test per compartment. This strategy identified differences in wound healing responses between gastric and progenitor-niches and the top genes that drive this signature in each cellular compartment.

##### Spatial transcriptomics communication module analysis

Our previous work showed that different cell types in the premalignant pancreas undergo concerted upregulation of communication modules (sets of receptor and ligand genes), and that this modularity that can be exploited to identify crosstalk between cell types that involves multiple cognate ligand and receptor pairs, suggesting robust and stable cell-cell interaction circuits^10^. To find evidence of communication in our spatial data, we identified pairs of communication modules that not only shared multiple cognate ligands and receptors, but were also upregulated in the same niches, a requirement for positive communication potential.

To compute cell-type-specific communication modules, we started from compartment-specific niche expression matrices of non-perturbed samples (excluding KC^shp53^ and MRTX1133-treated mice). The use of niche expression matrices overcomes sparsity in our spatial transcriptomics data, ensuring that dropouts do not dominate the signal and dilute out correlation estimates. We log-transformed the niche matrices (*pseudocount* = 1), standardized them over all niches and computed the gene–gene correlation matrix of communication genes, as defined by the CellChat database^48^ and distributed in the COMMOT repository^49^. Note that we only included communication genes with evidence of expression in the specified cellular compartment in our scRNA-seq data, and robust expression in our spatial data (see Compartment-aware gene censoring). Hierarchical clustering of the gene–gene correlation matrix revealed a modular architecture (Fig. 5a), which was preserved in single-cell correlation matrices, implying that compartment-specific average niche expression matrices largely aggregate coherent cell states within a cell type (Fig. S8a). As in our prior work^10^, large blocks of off-diagonal correlations suggested shared communication genes, motivating a soft-clustering approach that allows individual genes to participate in multiple communication modules.

To infer communication modules, we followed the same graph-based community detection strategy that we applied to dissociated single-cell data^10^. Briefly, we first constructed a gene–gene graph in which genes were connected if the Pearson correlation of their niche expression was larger than *ρ* = 0.2. Next we used a graph refinement approach based on each edge’s Jaccard similarity—the fraction of neighbors shared between two connected nodes in the graph, relative to the union of all neighbors of such nodes. Intuitively, this metric reveals tight communities of genes that are not only correlated in their expression, but also share many correlated genes. We removed edges with potentially spurious correlations (Jaccard similarity < 0.05) and added edges for genes that may not have reached the correlation threshold during graph construction, but that belong to a larger community of correlated genes (Jaccard similarity greater than 0.95). Lastly, we applied the Order Statistics Local Optimization Method (OSLOM) algorithm^50^ to identify compartment-specific communication modules, following prior work^10^. This algorithm finds potentially overlapping communities of genes in the refined gene–gene correlation graph. Application of OSLOM to gene–gene graphs constructed on niche expression of epithelial, immune and fibroblast compartments resulted in three distinct sets of communication modules (Fig. 5a).

The spatial dimension of our data allowed us to incorporate spatial co-expression when interpreting compartment-specific communication modules. For each module, we calculated the compartment-specific average *z*-scored niche expression of module genes in either progenitor or gastric niches (see Discretization of canonical gastric and progenitor niches). In each cellular compartment, we identified one or two modules with enriched expression in the progenitor niche, as quantified by an average *z*-scored expression > 0.2 (Fig. 5a). Thus, our approach defined a progenitor niche communication module in each cellular compartment.

To identify potential channels of intercellular communication in the progenitor niche, we identified cognate ligand–receptor pairs^48^ between progenitor niche communication modules in distinct cellular compartments. This approach resulted in a set of candidate communication channels that satisfied two criteria: (1) spatial co-occurrence in the progenitor niche, and (2) upregulation in progenitor niches, relative to gastric niches. We leveraged our standardized compartment-specific niche expression matrices (see *Summarization of niche features along the gastric–progenitor continuum*, third approach) to visualize concerted changes in the expression between cognate receptor–ligand pairs that may mediate heterotypic crosstalk in the progenitor niche (Fig. 5b and Fig. S8b). Specifically, we plotted the expression of a ligand in a specified cellular compartment across different niches. Our data suggested that collective upregulation of multiple communication genes in spatially co-occurring cell states may contribute to the formation and stabilization of the progenitor niche.

###### Tissue-level consequences of genetic and pharmacological perturbation

Our characterization of the injured premalignant pancreas revealed the tissue remodeling events that accompany the formation of progenitor niches at the morphological, cellular and molecular levels. These cancer-like wound-healing niches centered around progenitor-like cells, a subpopulation that simultaneously exhibited the highest engagement of tumor suppressive and oncogenic transcriptional signatures in the premalignant pancreas. Perturbation of *Kras* and *p53* signaling in epithelial cells profoundly impacted the abundance and state of this unique premalignant subpopulation: *p53* inactivation led to its expansion and adoption of advanced mesenchymal phenotypes (Fig. 7b–e), whereas KRAS inhibition rapidly depleted this cellular state (Fig. 6b). Next, we investigated how perturbation-induced changes in progenitor-like cells impacted non-epithelial cell types, particularly those associated with progenitor-like cells in the absence of additional interventions (e.g., *Tnc*+ myofibroblasts, *Itgax*+ macrophages/monocytes).

####### In silico dissection of pancreatic parenchyma.

An immediate challenge for analysis was the sample-to-sample heterogeneity in tissue structures outside of the pancreatic parenchyma, including lymph nodes and adipose tissue. These structures were larger and more prevalent when derived from whole-pancreas FFPE blocks than from samples comprising a fraction of the pancreas (collected as part of a cohort with multiple types of readout). Given that interlobular spaces and cell-dense lymph nodes could introduce technical variability into downstream analyses (due to tissue size and dissection strategy), we used spatial information to focus only on cells directly associated with the pancreatic epithelium. This *in silico* dissection is analogous to the manual removal of mesenteric lymph nodes in our prior work with dissociated cells, which was critical to avoid having the sheer density of their immune cells dominate cell-state quantification. Our strategy was to:

1. Define the pancreatic parenchyma as the set of all epithelial cells, plus neighboring cells within a 200-μm radius. Any cell outside this set was ignored.
2. Computationally identify lymph nodes. Starting from centroids corresponding to lymph-node-associated immune cells (B, CD4 T and CD8 T cells), we constructed a spatial neighbor graph connecting all cells within 30 μm of each other. We identified lymph nodes as connected components in the spatial neighbor graph with > 250 cells, a parameter that we manually tuned to capture visually identified lymph nodes. Cells in lymph nodes were computationally ignored.

After *in silico* dissection, the premalignant pancreas epithelium corresponded to 84% of cells in the data. We used this subset of cells to analyze differential abundance of tumor microenvironment cells in response to perturbation.

####### Shifts in microenvironmental states upon oncogenic KRAS inhibition.

Acute inhibition of oncogenic KRAS led to a dramatic reduction in progenitor-like cell abundance within 48 h of the first dose. To characterize the effect of this reduction on microenvironmental states and communication potential, we (1) quantified progenitor-like cell enrichment in the vicinity of different cell states, and (2) computed differential abundance with MiloR and interpreted results in light of the spatial association of enriched or depleted states with progenitor-like cells. We quantified and visualized changes in parenchyma cell-state abundance, excluding lymph nodes and interlobular spaces (see In silico dissection of pancreatic parenchyma).

We derived a quantitative scoring scheme to identify cellular states enriched near progenitor-like cells. For each niche anchored at a non-epithelial cell, we computed the fraction of progenitor-like or gastric-like cells relative to all epithelial cells within the niche (Fig. S9b–i). Next, we defined ‘progenitor enrichment’ as the log-ratio of the progenitor-like cell fraction, relative to the fraction of progenitor-like cells in the dataset. We defined ‘gastric enrichment’ following the same logic for gastric-like cells.

To identify a set of microenvironment states associated with progenitor-like cells, we selected cells with a progenitor enrichment score > 2, a threshold beyond which the gastric enrichment score sharply decreased. Furthermore, we required a gastric enrichment score < 0, thereby selecting for niches specifically enriched for progenitor-like cells relative to other premalignant states. This analysis resulted in (1) a continuous feature for each microenvironment cell, describing the extent of enrichment of progenitor-like cells in their vicinity, and (2) a discrete set of cells tightly associated with progenitor-like cells.

Density estimates on tumor microenvironment UMAP embeddings revealed that cell niches enriched with progenitor-like cells were depleted in MRTX1133-treated samples (Fig. 6c). To determine the significance of depletion, we applied the Milo algorithm^27^. Milo first constructs a set of tight and potentially overlapping cellular neighborhoods defined by transcriptional similarity on a kNN graph (makeNhoods function from miloR package, *prop* = 0.01, *k* = 30, *refined* = TRUE, *reduced_dims* = “PCA”, using pre-computed kNN and PCA), then tests for differences in cell counts between experimental conditions in each neighborhood using a generalized linear model with negative binomial residuals. Using Milo, we identified significantly enriched or depleted cell states upon acute oncogenic KRAS inhibition (SpatialFDR < 0.1).

To interpret differential abundance in light of defined cellular states, we annotated Milo neighborhoods by cell-state label, and found that 28% were composed of a single discrete label, representing pure cell states. For the remaining 72% of neighborhoods, we calculated the enrichment of the most frequent cell state relative to the second most frequent cell state. We conservatively excluded the 5% of Milo neighborhoods with lowest enrichment score (frequency of the most abundant cell state was less than 2.89-fold that of the second most abundant cell state), and labeled remaining neighborhoods by the predominant state. This strategy resulted in a set of transcriptional neighborhoods with a single cell-state label.

Lastly, we integrated spatial information into the interpretation of our differential abundance analysis. For each cell in a Milo neighborhood, we identified the cells in its niche, hereby termed Milo niche cells. Next, we calculated a progenitor enrichment score as the log ratio of the frequency of progenitor-like cells among Milo niche cells, relative to the frequency of progenitor-like cells in the dataset. We used this enrichment score to visualize the relationship between enrichment or depletion of neighborhoods, and their association with progenitor-like cells. Milo neighborhoods depleted upon acute oncogenic KRAS inhibition are strongly associated with progenitor-like cells in their niches (Fig. 6d).

###### Tissue-wide cell state proportions in KC^shp53^ and KC^shCtrl^ samples

To assess the tissue-wide consequences of *p53* loss in the injured pancreas, we quantified the frequency of cell-state proportions relative to their coarse cell type in each biological replicate. We restricted our analysis to the pancreatic parenchyma, to avoid potential confounding by large histological structures such as lymph nodes (see In silico dissection of pancreatic parenchyma). We used a two-tailed Wilcoxon rank sums test to assess the significance of differences in the tissue-wide frequency of cell states as a function of *p53* status.

###### Quantitative relationship between cell state frequencies in cellular niches

Knockdown of *p53* in premalignant epithelial cells led to the accumulation of progenitor-like cells with advanced mesenchymal phenotypes (Fig. 7b–f). These cells formed large tissue domains that could encompass entire pancreatic lobes (Fig. S11b,c), and were accompanied by the accumulation of *Itgax*+/*Cd274*^*high*^ (PD-L1^high^) macrophages/monocytes. Remarkably, the same tissue could contain regions devoid of progenitor-like cells and their associated microenvironments. This heterogeneity suggested that the local density of progenitor-like cells could determine properties of their microenvironment. To quantify such relationships, we determined cell-state frequencies relative to their coarse cellular compartment in cellular niches of different sizes. We log-transformed cell state frequencies, setting as pseudount the minimum non-zero value in each frequency vector. Next, we computed the joint distribution of the log frequency of progenitor-like cells (relative to epithelial cells in the niche) and the log frequency of *Itgax*+/*Cd274*^*high*^ cells (relative to myeloid cells in the niche). To visualize the cell-state relationship in a manner that is agnostic to the marginal distribution of the progenitor-like state frequency, we computed the distribution of *Itgax*+/*Cd274*^*high*^ cell frequencies conditioned on the frequency of progenitor-like cells in the niche, following the strategy outlined in DREVI^51^. Our analysis revealed that changes in the local density of progenitor-like cells are statistically related to changes in the frequency of their associated *Itgax*+/*Cd274*^*high*^ cells, suggesting also the presence of non-linearities in the quantitative relationship between the relative abundance of these two cell states.

###### Molecular programs in *Itgax*+/*Cd274*^*high*^ cells

To better understand the molecular properties of *Itgax*+/*Cd274*^*high*^ macrophages/monocytes that accumulated upon *p53* knockdown in premalignant pancreatic epithelial cells, we leveraged our myeloid-specific embedding of dissociated single cell data from KC^ctrl^ or KC^shp53^ mice three weeks post injury (see Analysis of premalignant tumor microenvironment data). First, we identified *Cd163*, *Maf*, *Itgax* and *Cd274* as four landmark genes that described progressive gene expression changes along the average gastric-progenitor niche axis in myeloid cells. Plotting and visualization of these four genes in UMAP embeddings of dissociated myeloid cells revealed that they captured gradual shifts in cell myeloid cell states in these data, consistent with spatial transcriptomics analyses (Fig. 11e). Moreover, we observed accumulation of Itgax+/Cd274^high^ cells in KC^shp53^ samples, corroborating our findings in spatial data, and providing the opportunity to study this subpopulation in greater depth. We used the wald test in the diffxpy package (v0.7.4, https://github.com/theislab/diffxpy?tab=readme-ov-file) and library size as numeric covariates. We identified upregulated genes using the following thresholds: *qval* < 0.05, log_2_ fold-change > 1. To highlight the top upregulated and downregulated genes from this analysis, we identified genes with mean_expression > 1, thus reporting changes in genes that are robustly expressed in at least one of the subpopulations (Fig. 11g).

## Supplementary Material

Supplement 1

Supplement 2

Supplement 3 **Data S2**. Oligonucleotides used for probing select mRNA expression using smFISH.

Supplement 4 **Data S1**. Custom probes for detection of GFP and mKate2 reporters in FFPE-based scRNA-seq.

Supplement 5 **Data S3**. Gene signatures used in this study.

1 **Supplementary Figure S1. Annotation of spontaneous tumorigenesis single cell data**. (**Related to Main** Figure 1). **a,b**. Projection of cells from pre-tumor (a) or tumor (b) stage samples into force directed layouts of scRNA-seq data. Each dot is a single cell colored by sample of origin. **c**. Representative FACS plot showing frequency of mKate2+/GFP+ (p53 proficient) or mKate2+/GFP− (p53 deficient) cells harvested from 4.5 months old KP^LOH^ mouse. **d**. Projection of GFP mRNA expression in individual Kras^G12D^+ epithelial cells visualized in a force-directed layout. **e**. Expression of transcriptional signatures from major premalignant cell states derived from Burdziak, Alonso-Curbelo et al.^37^ in premalignant cells from pre-tumor stage mice. p53-deficient cells from PDAC samples, or microtumor clusters are grayed-out. **f**. Expression of transcriptional signatures from major premalignant cell states in premalignant cell clusters from pre-tumor stage mice. Clusters were identified using PhenoGraph (k=30). Signatures were computed as the average z-scored expression of signature genes in each cell. Scores were then averaged over all cells from a single cluster. Average signatures were standardized over all PhenoGraph clusters for cluster annotation and visualization. **g**. Visualization of diffusion component 2 (DC2) in force directed layout. DC2 captured continuity between gastric-like and progenitor-like premalignant cells. **h**. Distribution of number of cells along DC2. Dashed line represents the DC2 threshold value used to identify progenitor-like cells. **i**. Discretization of the gastric-progenitor continuum using threshold DC2 threshold identified in (h). **j**. Expression of marker genes for distinct premalignant states and PDAC in distinct subpopulations of Kras^G12D^+ epithelial cells. Dot size represents the fraction of cells in the specified cell state that express the gene. Color represents the average gene expression in cells that express the gene. **k**. Distributions of premalignant states as a function of sample and p53 status.
**Supplementary Figure S2. Identification of microscopic PDAC in pre-tumor stage mice**. (**Related to Main** Figure 1). **a**. Inferred karyotypes from single cell transcriptomes. Each row in karyotype matrices corresponds to a single cell, and columns correspond to genes ordered by chromosomal location. Colors represent estimates of copy number change. Cells are grouped in distinct blocks based on PDAC development stage (pre-tumor vs tumor), p53 status (proficient vs deficient), and the extent of karyotype changes. Within each block, cells are grouped by sample of origin, and clustered by karyotype profile. Dashed lines relate cells from the same sample of origin in different karyotype classes. **b**. Representative immunofluorescence images of microscopic PDAC detected in KP^LOH^ mice. Slides were stained for mKate2 and GFP to assess p53 genetic status, as well as the progenitor state marker HMGA2. **c**. Karyotype profiles of p53 deficient cells in a mouse harvested at the pre-tumor stage. A subset of cells show karyotype and transcriptional profiles consistent with PDAC (microtumor cells). Another subset shows karyotype and transcriptional profiles consistent with progenitor-like and other premalignant states. Boxes highlight groups of individual cells with premalignant transcriptional profiles that share a subset of karyotype changes present in microtumor cells. **c**. Projection of individual p53 deficient cells from a single sample, colored based on their karyotype status. Pink cells with dark outline denote premalignant-like cells with a subset of karyotype changes shared with microtumor cells.
**Supplementary Figure S3. Progenitor-like signatures in individual mouse and human samples** (**Related to Main** Figure 2). **a**. Expression of canonical p53 targets and progenitor state markers as a function of p53 status. Each row documents summarized expression within individual samples. Parenthesis show the number of cells aggregated in each row. **b**. Gene set enrichment analysis computed using log_2_ fold changes in gene expression between p53 proficient progenitor-like cells and other p53 proficient premalignant cells, as estimated by differential gene expression analysis (see Methods). **c**. Expression of representative genes upregulated in progenitor-like cells, corresponding to tumor suppressive and oncogenic signatures shown in Main Fig. 2d. **d**. Fraction of isoform-specific *Cdkn2a* reads in pre-tumor p53 proficient samples. **e**. Distribution of oncogenic and tumor suppressive signature scores shown in Main Fig. 2d in p53 proficient cells, as a function of cell state (progenitor-like or other). **e**. Average z-scored expression of representative genes from oncogenic and tumor suppressive signatures associated with the progenitor-like state in human-derived single cell data from Carpenter, Elhossiny, Kadiyala, et al.^3^. Rows in the middle block correspond to PhenoGraph clusters of ADM/duct-like cells (see Methods). Box highlights PhenoGraph clusters with the highest expression of progenitor-like signatures in Main Fig. 2f. **e**. Projection of individual cells from human pancreatic samples in UMAP. ADM/duct-like cells are outlined. Cells in PhenoGraph clusters with highest progenitor-like signatures are colored based on donor of origin.
**Supplementary Figure S4. Identification of progenitor-like lesions upon pancreatic injury** (**Related to Main** Figure 3). **a**. Staining of progenitor-like markers in tissue section from a 6 weeks old pre-tumor stage KP^LOH^ mouse 2 days post pancreatitis. Data was collected using the Lunaphore COMET multiplex immunofluorescence platform. White box highlights a progenitor lesion. Side fields of view show overlays of individual progenitor markers with counterstains for premalignant epithelial (GFP) and nuclei (DAPI). **b**. Immunofluorescence staining and quantification of p53 protein levels in 6–7 weeks old KC^shRen^ mice 2 days post pancreatitis. Images show staining for MSN to identify progenitor lesions (left) and staining for p53 in an adjacent lesion (right). Cyan and magenta outlines highlight epithelial lesions manually identified as either MSN− or MSN+, respectively, based on comparison between adjacent tissue sections, blinded for p53 staining. **c**. Immunofluorescence staining of the mesenchymal marker VIM in KC^shRen^ mice harvested 3 weeks post pancreatitis.
**Supplementary Figure S5. Use of reference single cell dataset and smFISH staining for determining expression thresholds for Xenium panel design** (**Related to Main** Figures 3 **and** 4). **a**. Distribution of gene expression in single cell reference datasets. Each dot represents a meta-cell computed using SEACells^121^ (see Methods). Expression values are plotted in linear scale. Genes are ordered by their highest expression in any SEACell. Dashed lines represent expression thresholds that guided selection of markers to include in our Xenium panel (see Methods). **b**. Representative smFISH staining of genes shown in (a) in the premalignant pancreas. Regions of interest, highlighting positive cells, are shown in high resolution below each tissue image. Scale bars 20 μm.
**Supplementary Figure S6. Spatial patterning of transcriptional heterogeneity in distinct cellular compartments of the premalignant pancreas** (**Related to Main** Figures 3–5). (Left panels) Representative images of spatial organization of transcriptional clusters identified in major cellular compartments in Xenium data (see Methods). Each dot represents a single cell centroid, and cells are colored based on transcriptional state. The tissue shown is derived from a KC^shRen^ mouse, 3 weeks post-pancreatitis. (Middle panels) UMAP visualization of transcriptional clusters depicted in left panels. (Right panels) Xenium-based quantification of representative marker genes for transcriptional clusters identified in each cellular compartment.
**Supplementary Figure S7. Molecular and compositional properties of the progenitor niche** (**Related to Main** Figure 4). **a**. Distribution of coarse cell types in niches as a function of the average gastric-progenitor DC in the niche (see Main Text and Methods) in Xenium samples from mice with Kras mutant, p53 proficient pancreatic epithelium (pre-tumor KC^shRen^ or KP^LOH^), pooling tissue samples 2 days and 3 weeks post injury. **b**. Expression of select mRNA markers of microenvironment states associated with gastric or progenitor niches. Each dot is an mRNA. Circles represent niches highlighted in Main Fig. 4d. **c**. Frequency of select cell populations as a function of average gastric–progenitor DC in niche epithelial cells. Bold lines, median fraction; shaded areas, interquartile range. **d-e**. Analysis of gene expression heterogeneity in (d) monocyte/macrophages and (e) myofibroblasts from genome-wide dissociated single cell data. Data was collected from 2 mice with Kras mutant, p53 proficient pancreatic epithelium (KC^shRen^ or KC^shp53^ without doxycycline induction of shp53), 3 weeks post pancreatitis. Cells are aligned and grouped in 50 bins along the major axis of variation within each cellular compartment, as defined by diffusion component 1 (DC1). (Top panels) Expression of genes measured in our Xenium panel and depicted in Main Fig. 4f, preserving the order of rows for ease of comparison. Bottom panels include expression of genes not measured in our Xenium panel that inform broader expression programs activated in the progenitor niche. **f**. Average expression of wound-healing response genes (GO Biological Processes) with highest upregulation in progenitor relative to gastric niches, in different cellular compartments. Each dot represents a single biological replicate (n=15 mice). Values denote the average z-scored expression of wound-healing genes in either gastric or progenitor niches. P-values, two-tailed Wilcoxon Rank Sums Test.
**Supplementary Figure S8. Concerted upregulation of cognate ligand-receptor pairs in the progenitor niche** (**Related to Main** Figure 5). **a**. Expression of representative cognate ligand-receptor pairs with coordinated changes in expression as a function of the average gastric-progenitor DC in the niche. Each dot is the averaged z-scored niche expression of the specified genes in their corresponding cellular compartment (Methods). The color and size of the dots corresponds to the relative frequency of gastric or progenitor-like cells in the niche. **b**. Expression of markers associated with the progenitor niche in epithelial, immune and fibroblasts. Each dot is an individual mRNA, colored depending on the producer cell type. **c**. Log normalized niche expression of *Tgfbr1* and *Tgfbr2* in gastric (navy) or progenitor-like (red) niches, as a function of cellular compartment. Dashed lines denote 0 expression. **d**. Dot plot of dissociated single cell expression data in pancreatic epithelial cells. First row: acinar cells from uninjured mice^37^; second row: ADM cells 2 days post pancreatic injury in the context of WT Kras^37^; third row: KrasG12D+ cells outside of the progenitor-like state, harvested 3 weeks post-injury (this work); fourth row: KrasG12D+ progenitor-like state harvested 3 weeks post-injury (this work). Dot size represents the fraction of cells that express a given mRNA in the group. Dot color represents the level of expression in positive cells within the group.
**Supplementary Figure S9. Consequences of acute oncogenic Kras inhibition in premalignant cells** (**Related to Main** Figure 6). **a**. Representative images and quantification of p-ERK staining in KP^LOH^ mice treated with vehicle or MRTX-1133d. Median p-ERK signal 2 days after vehicle (n = 3) or MRTX1133 treatment (n = 4). **b**. Force-directed layout visualization of single cell transcriptomic data from Kras^G12D^+/p53 proficient pancreatic epithelial cells derived from MRTX1133 (n=3) or vehicle (n=3) treated mice during injury-induced pancreatitis. Panels show two-dimensional density maps of premalignant states (left) or treatment condition (right). **c**. Differential abundance testing for differences between MRTX1133 or vehicle-treated mice using MiloR^84^. Each dot is a transcriptional neighborhood of single cells. The size of the dots scales with SpatialFDR. Outlined dots show transcriptional neighborhoods that were differentially abundant between conditions (SpatialFDR < 0.1). Transcriptional neighborhoods are grouped and colored by the most abundant cell state in the neighborhood. **d**. Differences in the expression of transcriptional signatures in ADM cells as a function of treatment (MRTX1133 vs vehicle). The log_2_ fold change in gene expression between conditions, as estimated by differential gene expression analysis using diffxpy (https://github.com/theislab/diffxpy), was used as the ranking variable for gene set enrichment analysis. All signatures shown are enriched or depleted with FDR < 0.1. Top up- and down-regulated pathways from unbiased analysis are shown. **e**. Expression of representative genes for select gene sets identified in (d) as a function of treatment. Each row represents a biological replicate, denoting the number of cells in the group in parenthesis. **f**. Differences in the expression of oncogenic and tumor suppressive signatures shown in Main Fig. 6c, as a function of treatment (MRTX1133 or vehicle, n=3 mice each). Dots represent individual biological replicates. Expression signatures were computed as the average z-scored expression of signature genes in individual cells. Signatures were then averaged across all cells in a biological replicate for a final sample-level score. P-values result from rank sums test. **g**. Expression of oncogenic and tumor suppressive signatures in single cell data from MRTX1133 (n=3 mice) or vehicle (n=3 mice) treated KP^LOH^ mice. Cells were aggregated by condition, or cell state. Parentheses indicate the total number of cells per group.
**Supplementary Figure S10. Consequences of acute oncogenic Kras inhibition in premalignant niches** (**Related to Main** Figure 6). **a**. Representative Xenium images of whole tissue sections from vehicle (left) or MRTX1133 (right) pre-tumor stage KP^LOH^ treated mice. Each dot is a single cell centroid colored by select cell states that demarcate differences between gastric and progenitor niches. **b**. Integration of niche-level analysis with differential abundance testing (i) For each cell, we calculate the fraction of epithelial cells annotated as either progenitor-like or gastric-like in their niche. The plot highlights two fibroblast cells that differ in the fraction of progenitor-like cells in their vicinity. The progenitor enrichment score is defined as the log ratio of the fraction of progenitor-like cells in the niche and the fraction of progenitor-like cells in the tissue. (ii) Systematic quantification of the progenitor enrichment score results in a tissue map that reflects continuous spatial variation in the abundance of progenitor-like cells in the tissue. Fibroblasts in the image are colored by their progenitor enrichment score. (iii) Construction of transcriptional neighborhoods as implemented in MiloR. For each cell in a Milo neighborhood, we extract epithelial cells in their niche (bottom). (iv) We use the fraction of progenitor-like cells relative to other epithelial cells in the niche of cells identified in (iii) to compute a progenitor enrichment score for every Milo neighborhood (see Methods). **c**. Two-dimensional density representation of Xenium single-cell gene expression data from mice treated with vehicle (n = 2) or MRTX1133 (n = 4). Purple contours, density of transcriptional states in the vicinity of progenitor-like epithelial cells; gold contours, density of states depleted after MRTX1133 treatment; royal blue contours, density of states enriched after MRTX1133 treatment.
**Supplementary Figure S11. Transcriptional consequences of *p53* loss in the context of oncogenic Kras activation and pancreatic injury** (**Related to Main** Figure 7). **a**. Immunofluorescence-based quantification of the number of HMGA2+ (left) or VIM+ (right) epithelial cells in KC^shp53^ or KC^shCtrl^ mice 3 weeks post-pancreatitis. Each represents a single mouse, bars are grouped by cohort, matching pooled data in Fig. 7a,e. **b**. scRNA-seq based quantification of progenitor-like cells or Vim+ progenitor-like cells as a function of p53 knockdown status, 3 weeks post-pancreatitis. Each dot is a single biological replicate. p-value results from Two-tailed Wilcoxon Rank Sums Test. **c-e**. (Left) Force-directed layouts of Kras^G12D^+ pancreatic epithelial cells derived from KC^shCtrl^ (n=4 mice) or KC^shp53^ (n=5 mice) samples, 3 weeks post caerulein-induced pancreatitis. Box highlights progenitor-like clusters. Cells are colored by *p53* knockdown status (a,b) or by cell state (c). (Right) expression of canonical p53 targets and epithelial markers in progenitor-like clusters. Each row represents a biological replicate. The number of cells in each group is denoted in parenthesis. **a**. All progenitor-like cells in individual biological replicates were aggregated to summarize gene expression regardless of progression along an epithelial-mesenchymal plasticity axis. **b**. The PhenoGraph cluster that accumulates in KC^shp53^, but is rare in KC^shCtrl^ was excluded from aggregation. **c**. KC^shp53^ cells were aggregated as a function of PhenoGraph cluster assignment (termed progenitor1 and progenitor2). **d**. Differences in the expression of transcriptional signatures in progenitor-like cells from KC^shp53^ mice as a function of treatment (progression along the epithelial-mesenchymal plasticity axis). Differential gene expression analysis using diffxpy (https://github.com/theislab/diffxpy) was used to estimate log2 fold changes in gene expression between progenitor 1 and progenitor 2 shp53 cells—as defined by PhenoGraph clustering. The log2 fold change in gene expression was used as the ranking variable for gene set enrichment analysis. All signatures shown are enriched or depleted with FDR < 0.1. **e**. Expression of representative genes of select signatures enriched in progenitor2 vs progenitor1 cells, as identified in (d). Columns represent individual biological replicates, grouped by premalignant cell state. Parentheses denote the number of cells per group.
**Supplementary Figure S12. Tissue remodeling upon p53 knockdown in the context of oncogenic Kras and pancreatic injury** (**Related to Main** Figure 7). **a**. Frequency of cell states in epithelial, myeloid cells and fibroblasts, as a function of p53 proficiency in the premalignant epithelium: shp53 (n=9) or ctrl samples (n=5). All samples were harvested 3 weeks post-pancreatic injury. Each dot denotes a single biological replicate. P-values, Two-tailed Wilcoxon Rank Sums Test. **b**. Representative Xenium data of epithelial composition in control or p53 knockdown tissue. Each dot is a single cell centroid, colored by cell state. **c**. Visualization of progenitor niches in our entire collection of KC^shRen^ and KC^shp53^ tissues collected 3 weeks post-pancreatitis. Each dot is a single cell colored by cell state. Boxes denote regions highlighted in (b). **d**. UMAP visualization of macrophage/monocyte cells in dissociated single cell data from the premalignant pancreas. Cells were harvested from mice with p53 knockdown in the premalignant epithelium or (n=2) or control (n=2), 3 weeks post-injury. Cells are colored by condition of origin. **e**. Visualization of gene expression of select markers of myeloid subpopulations. Each cell is pseudocolored by the collective expression of specified markers. (top) log-normalized expression, (bottom) MAGIC imputed counts (kNN with k=30, kernel width = 10, t=1). **f**. Distribution of the abundance of *Itgax*+/*Cd274*^high^ cells (fraction of myeloid), conditioned on the abundance of progenitor-like cells (fraction of epithelial) in the niche. Red line denotes. Axes are presented in log scale (left) or linear scale (right). **g**. Subset of genes differentially expressed in *Itgax*+/*Cd274*^high^ vs *Itgax*+/*Cd274*^low^ cells. The first gene group shows differentially-expressed genes highlighted due to known roles in immune regulation and tissue injury. The second and third groups show the top 10 upregulated and downregulated genes in *Itgax*+/*Cd274*^high^ cells.
**Note S1**. Construction of diffusion operator and computation of diffusion maps.
**Table S1**. Metadata of scRNA-seq produced in this study.
**Table S2**. smFISH probe metadata (Related to Fig. S5)
**Table S3**. Embedding of TME subsets in injury induced tumorigenesis dissociated scRNA-seq (Related to Fig. S7 and Fig. S12)
**Table S5**. Xenium sample information and embeddings (Related to Fig. 3, Fig. 4, Fig. 5, Fig. 6, Fig. 7).
**Table S6**. Compartment- and condition-specific embeddings for Xenium samples.
**Table S7**. Statistics on mixed cell states in Xenium data (Related to Fig. S6)
**Table S8**. Gene censoring in Xenium data (Related to Fig. 4, Fig. 5, Fig. S7, Fig. S8)

**Table S4**. Custom 10x Xenium library design with annotations of cellular compartment or biological processes probed by each gene.

**Data S4**. Source data for immunofluorescence.
