# Supplementary material for: Oncogenic and tumor-suppressive forces converge on a progenitor-orchestrated niche to shape early tumorigenesis
Source: bioRxiv. 2025 Jun 12:2025.06.10.656791. Preprint. [Version 1] doi: 10.1101/2025.06.10.656791 (PMC12259082; doi:10.1101/2025.06.10.656791)
Supplement: 1 — Supplementary Figure S1. Annotation of spontaneous tumorigenesis single cell data. (Related to Main Figure 1). a,b. Projection of cells from pre-tumor (a) or tumor (b) stage samples into force directed layouts of scRNA-seq data. Each dot is a single cell colored by sample of origin. c. Representative FACS plot showing frequency of mKate2+/GFP+ (p53 proficient) or mKate2+/GFP− (p53 deficient) cells harvested from 4.5 months old KPLOH mouse. d. Projection of GFP mRNA expression in individual KrasG12D+ epithelial cells visualized in a force-directed layout. e. Expression of transcriptional signatures from major premalignant cell states derived from Burdziak, Alonso-Curbelo et al.37 in premalignant cells from pre-tumor stage mice. p53-deficient cells from PDAC samples, or microtumor clusters are grayed-out. f. Expression of transcriptional signatures from major premalignant cell states in premalignant cell clusters from pre-tumor stage mice. Clusters were identified using PhenoGraph (k=30). Signatures were computed as the average z-scored expression of signature genes in each cell. Scores were then averaged over all cells from a single cluster. Average signatures were standardized over all PhenoGraph clusters for cluster annotation and visualization. g. Visualization of diffusion component 2 (DC2) in force directed layout. DC2 captured continuity between gastric-like and progenitor-like premalignant cells. h. Distribution of number of cells along DC2. Dashed line represents the DC2 threshold value used to identify progenitor-like cells. i. Discretization of the gastric-progenitor continuum using threshold DC2 threshold identified in (h). j. Expression of marker genes for distinct premalignant states and PDAC in distinct subpopulations of KrasG12D+ epithelial cells. Dot size represents the fraction of cells in the specified cell state that express the gene. Color represents the average gene expression in cells that express the gene. k. Distributions of premalignant state [file NIHPP2025.06.10.656791V1-supplement-1.pdf]

bioRxiv preprint doi: https://doi.org/10.1101/2025.06.10.656791; this version posted June 12, 2025. The copyright holder for this preprint (which was not certified by peer review) is the author/funder, who has granted bioRxiv a license to display the preprint in perpetuity. It is made available under aCC-BY 4.0 International license.

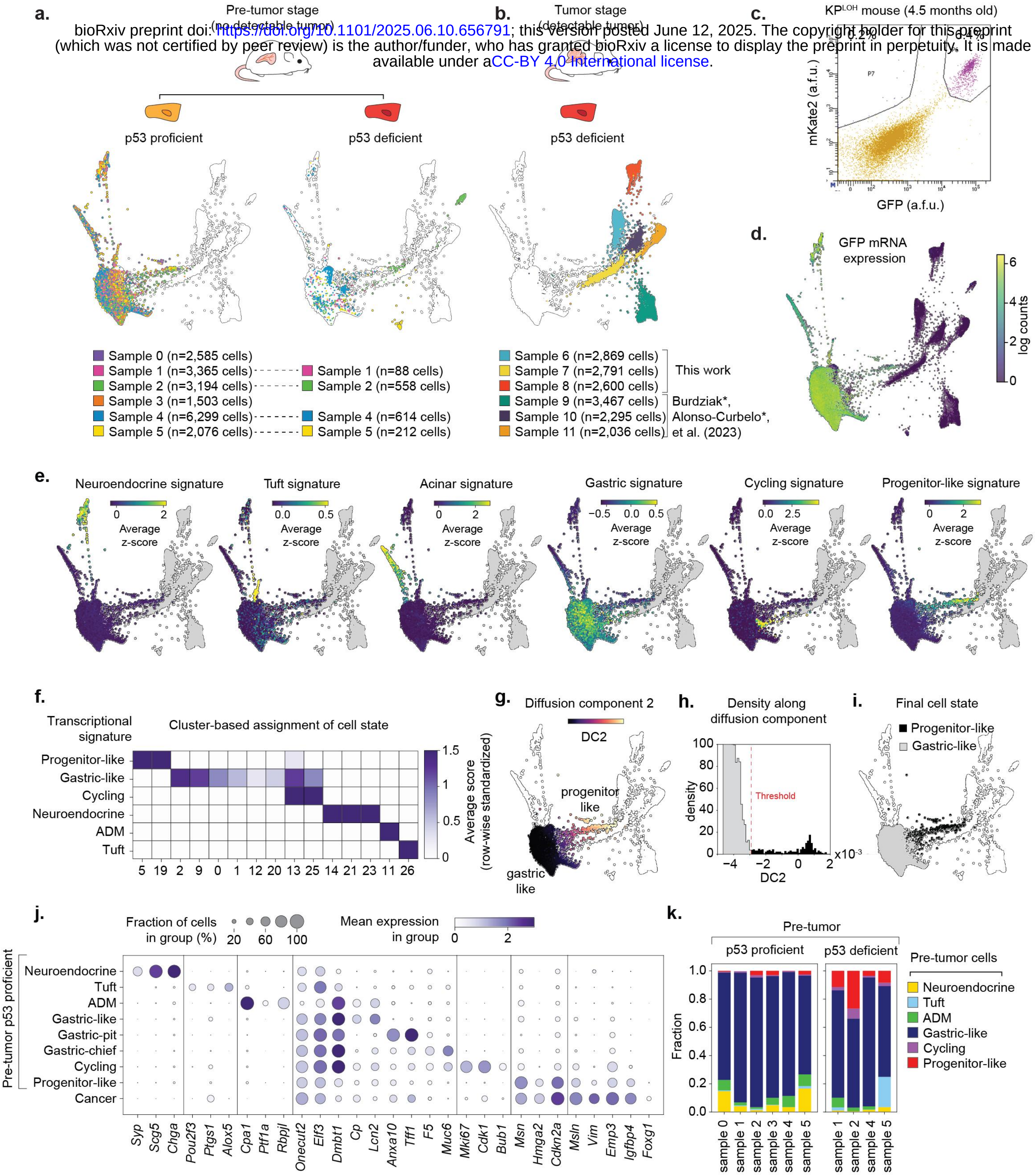

Supplementary Figure S1. Annotation of spontaneous tumorigenesis single cell data. (Related to Main Figure 1).

bioRxiv preprint doi: https://doi.org/10.1101/2025.06.10.656791; this version posted June 12, 2025. The copyright holder for this preprint (which was not certified by peer review) is the author/funder, who has granted bioRxiv a license to display the preprint in perpetuity. It is made available under aCC-BY 4.0 International license.

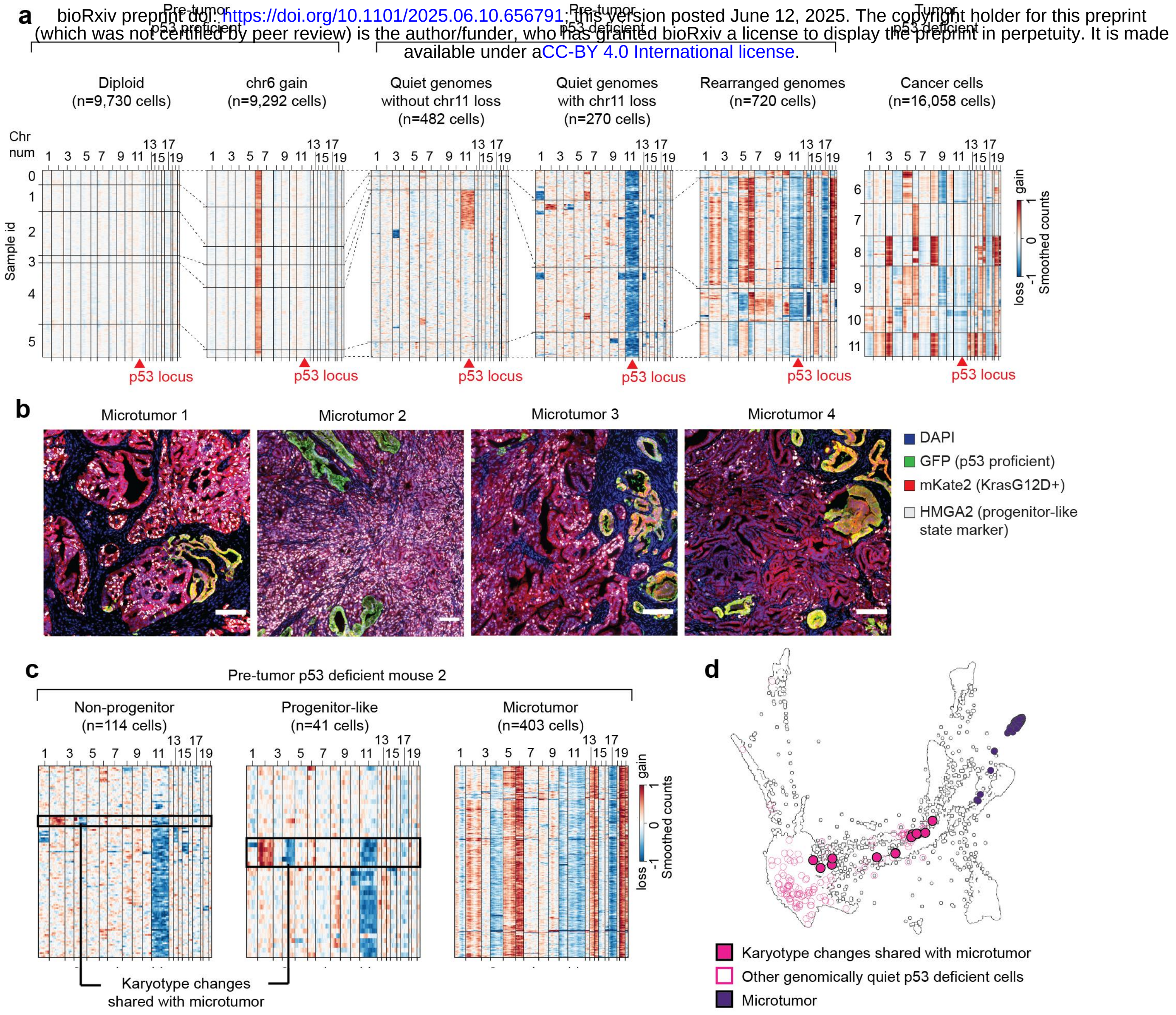

Supplementary Figure S2. Identification of microscopic PDAC in pre-tumor stage mice. (Related to Main Figure 1).

bioRxiv preprint doi: https://doi.org/10.1101/2025.06.10.656791; this version posted June 12, 2025. The copyright holder for this preprint (which was not certified by peer review) is the author/funder, who has granted bioRxiv a license to display the preprint in perpetuity. It is made available under aCC-BY 4.0 International license.

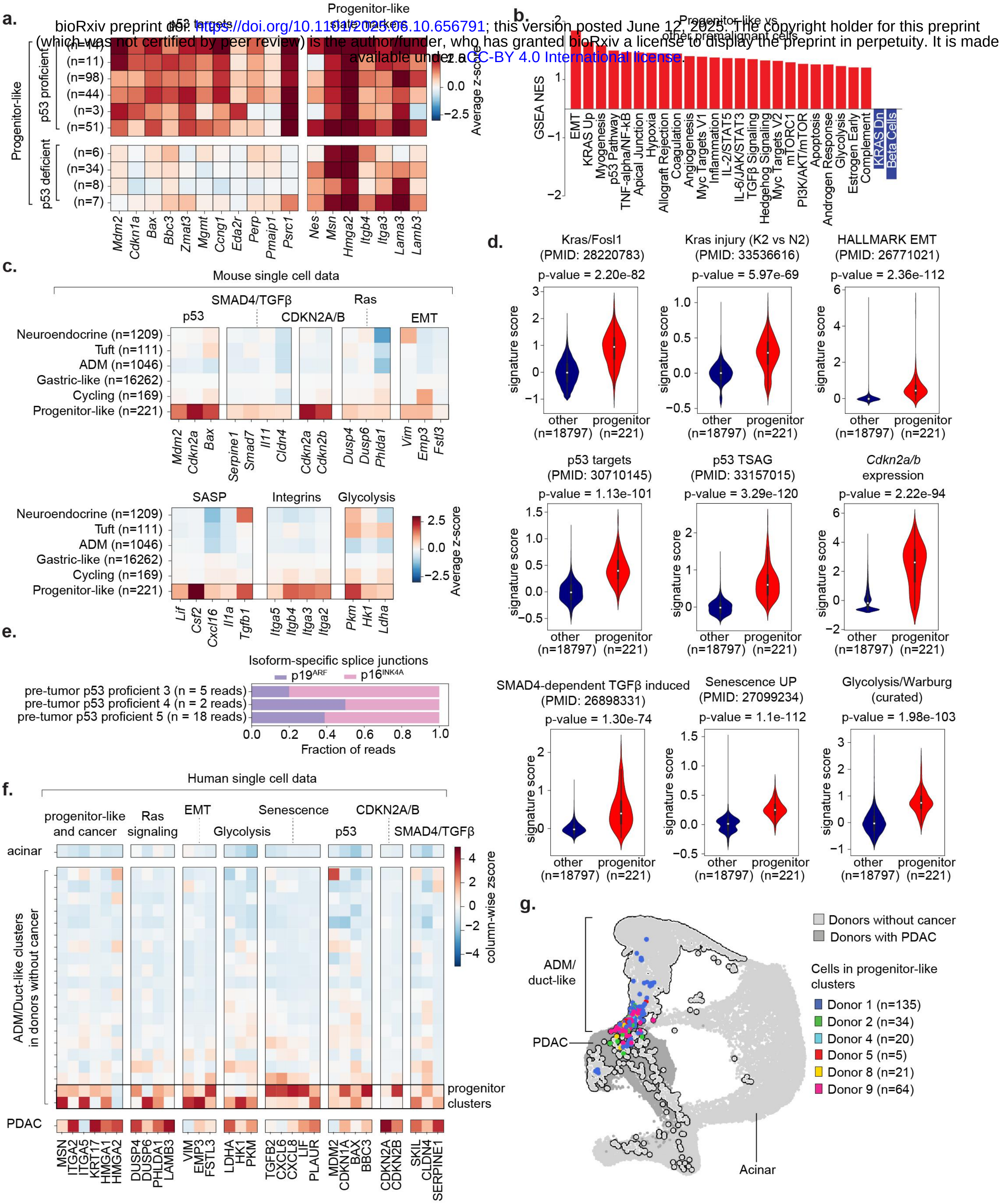

Supplementary Figure S3. Progenitor-like signatures in individual mouse and human samples (Related to Main Figure 2).

bioRxiv preprint doi: https://doi.org/10.1101/2025.06.10.656791; this version posted June 12, 2025. The copyright holder for this preprint (which was not certified by peer review) is the author/funder, who has granted bioRxiv a license to display the preprint in perpetuity. It is made available under aCC-BY 4.0 International license.

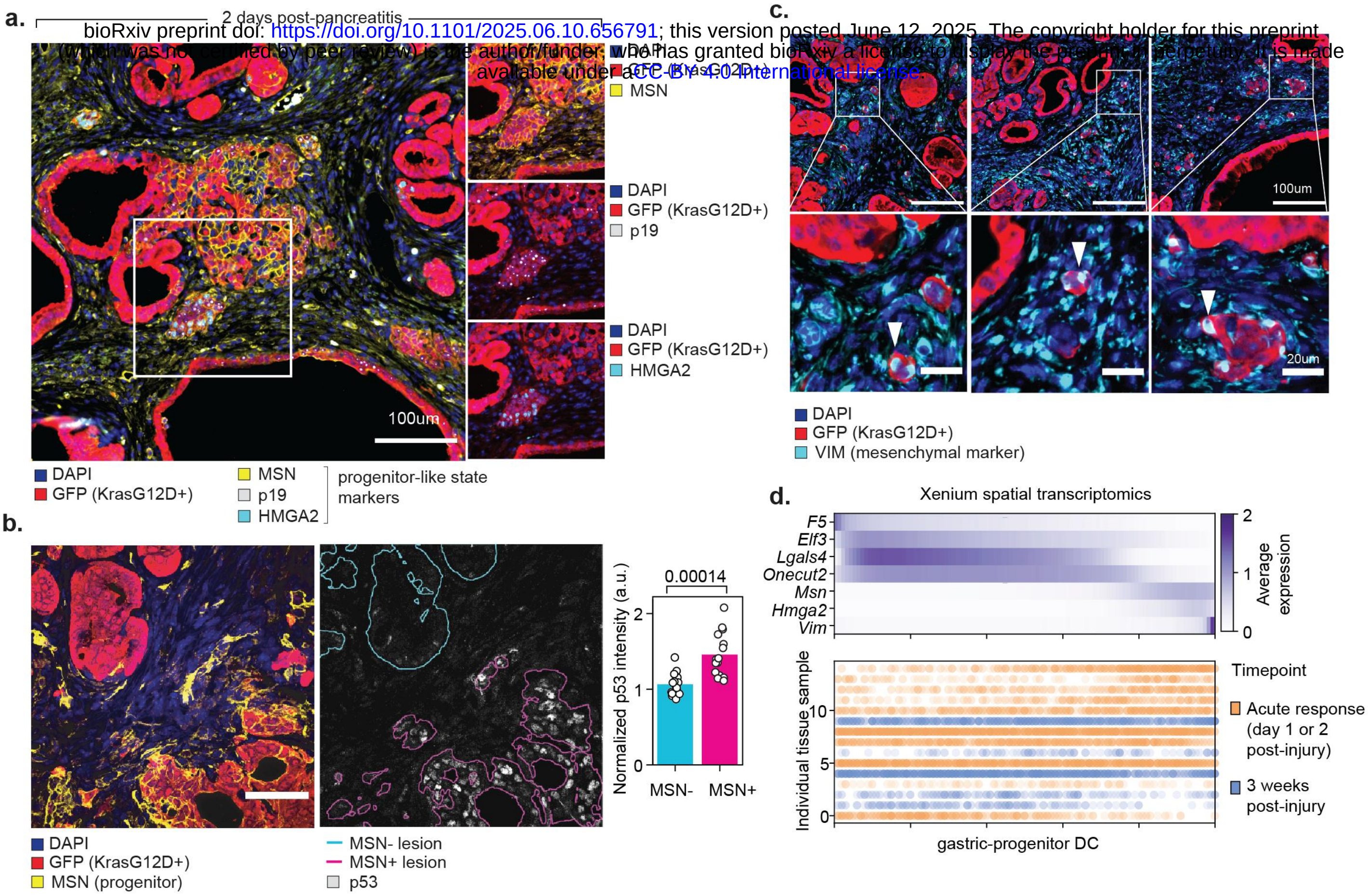

Supplementary Figure S4. Identification of progenitor-like lesions upon pancreatic injury (Related to Main Figure 3).

bioRxiv preprint doi: https://doi.org/10.1101/2025.06.10.656791; this version posted June 12, 2025. The copyright holder for this preprint (which was not certified by peer review) is the author/funder, who has granted bioRxiv a license to display the preprint in perpetuity. It is made available under aCC-BY 4.0 International license.

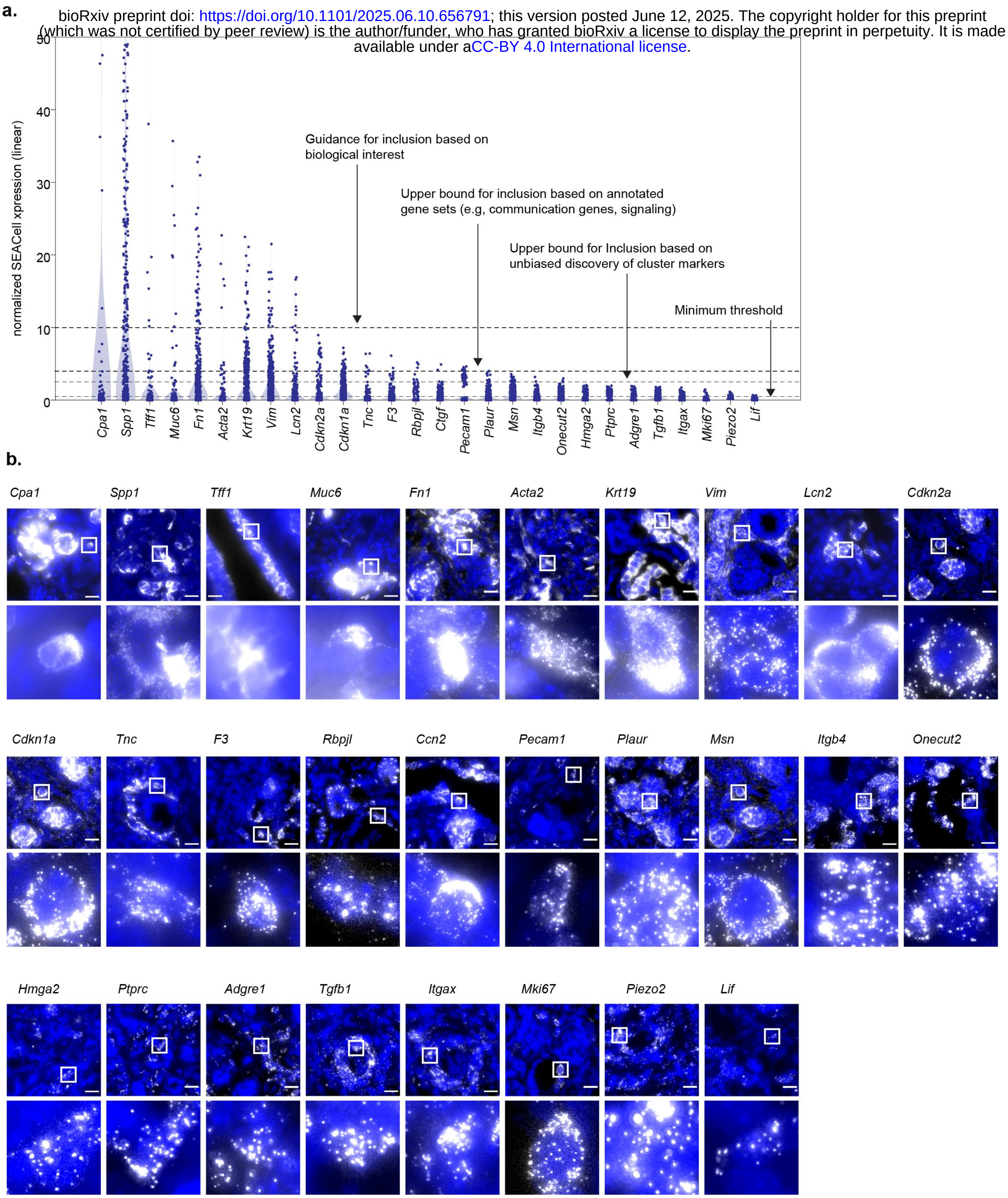

Supplementary Figure S5. Use of reference single cell dataset and smFISH staining for determining expression thresholds for Xenium panel design (Related to Main Figures 3 and 4).

bioRxiv preprint doi: https://doi.org/10.1101/2025.06.10.656791; this version posted June 12, 2025. The copyright holder for this preprint (which was not certified by peer review) is the author/funder, who has granted bioRxiv a license to display the preprint in perpetuity. It is made available under aCC-BY 4.0 International license.

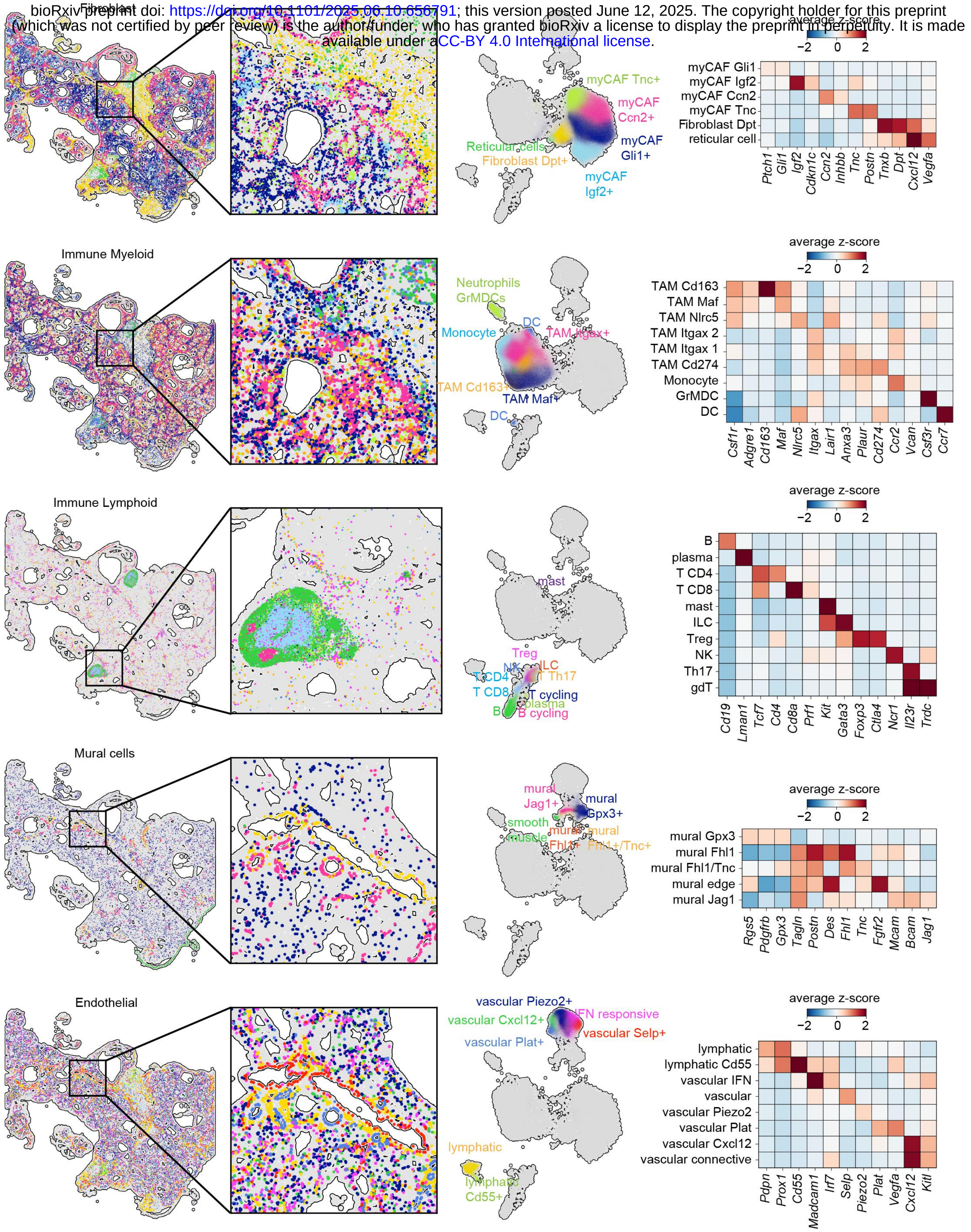

Supplementary Figure S6. Spatial patterning of transcriptional heterogeneity in distinct cellular compartments of the premalignant pancreas (Related to Main Figures 3-5).

bioRxiv preprint doi: https://doi.org/10.1101/2025.06.10.656791; this version posted June 12, 2025. The copyright holder for this preprint (which was not certified by peer review) is the author/funder, who has granted bioRxiv a license to display the preprint in perpetuity. It is made available under aCC-BY 4.0 International license.

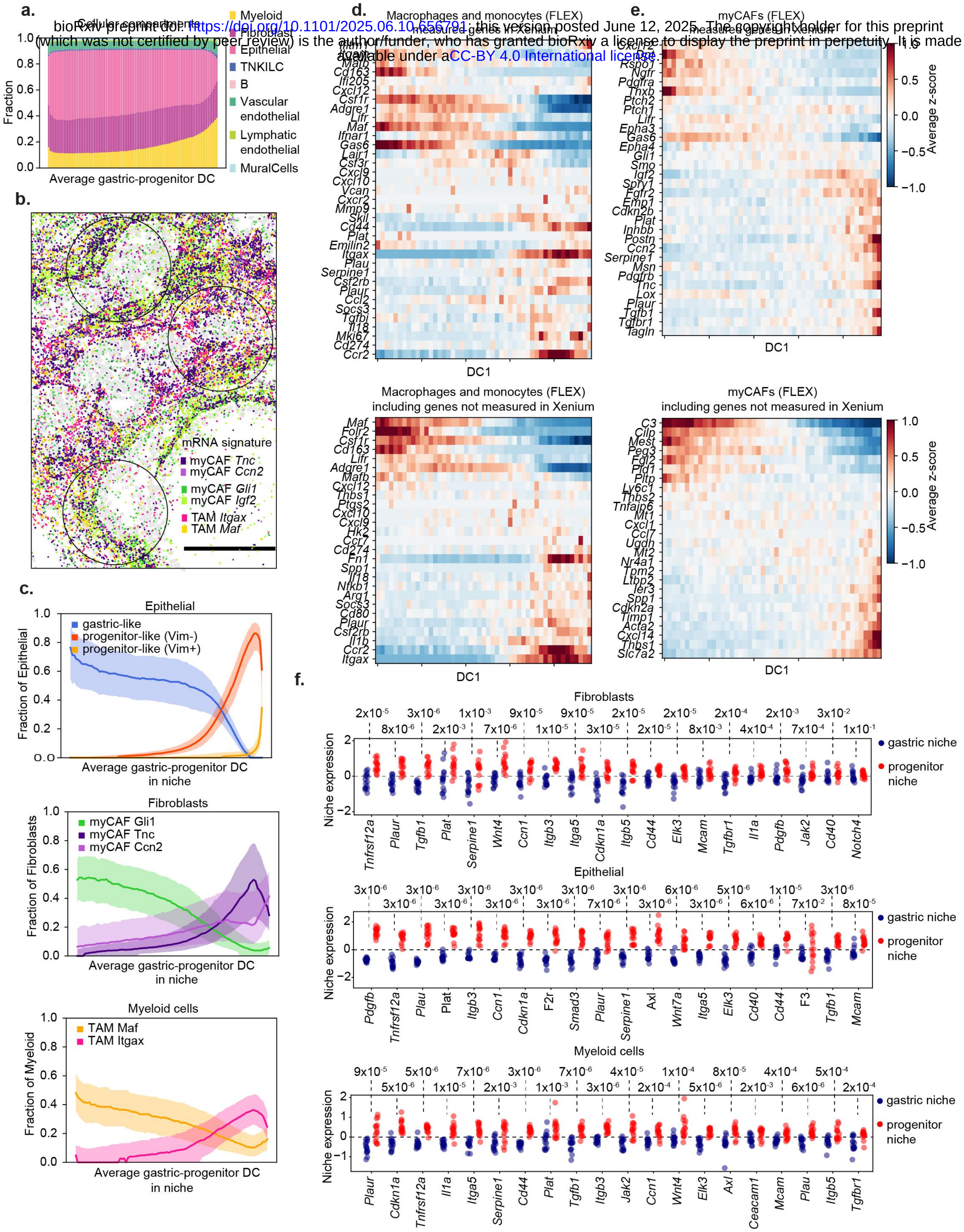

Supplementary Figure S7. Molecular and compositional properties of the progenitor niche (Related to Main Figure 4).

bioRxiv preprint doi: https://doi.org/10.1101/2025.06.10.656791; this version posted June 12, 2025. The copyright holder for this preprint (which was not certified by peer review) is the author/funder, who has granted bioRxiv a license to display the preprint in perpetuity. It is made available under aCC-BY 4.0 International license.

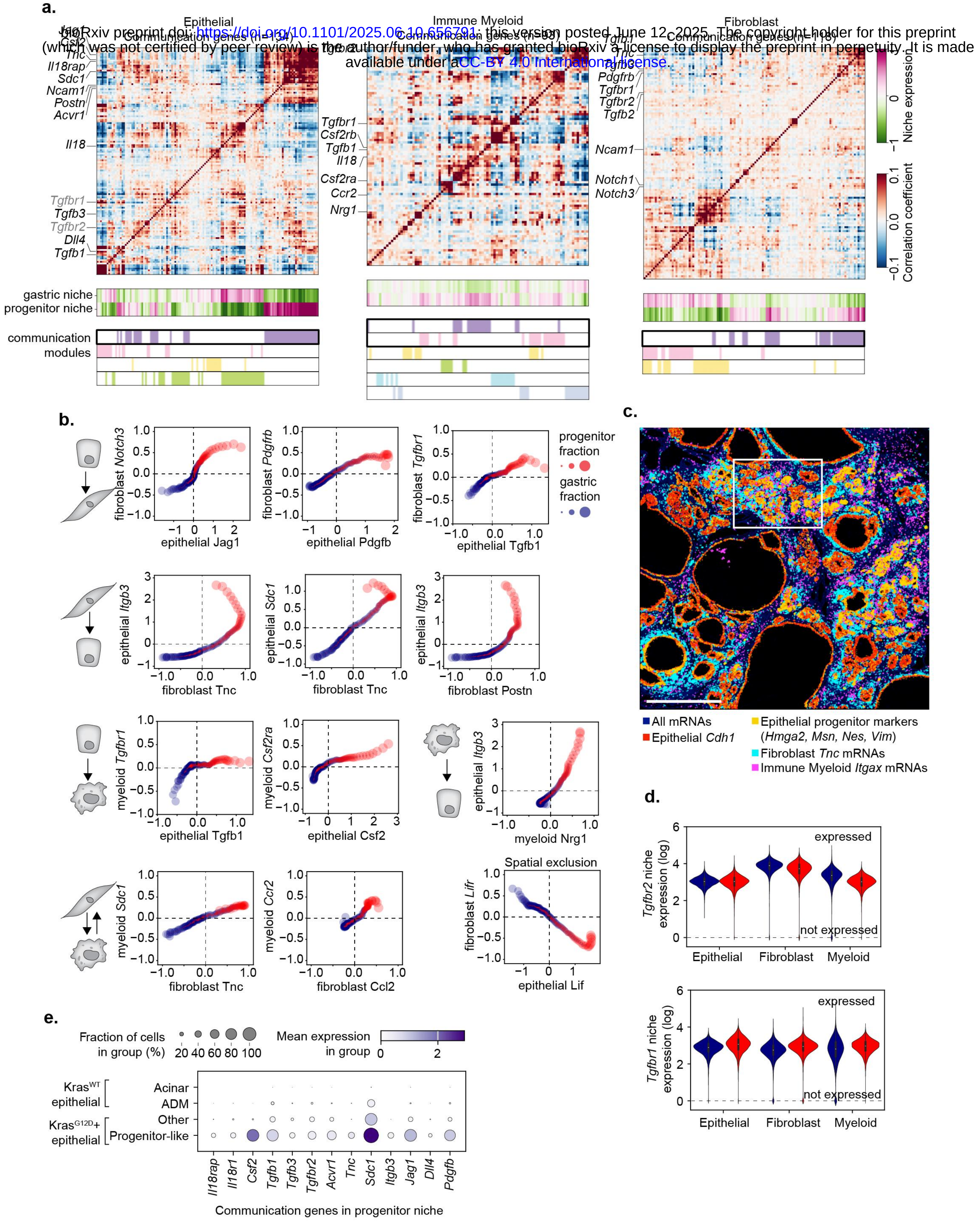

Supplementary Figure S8. Concerted upregulation of cognate ligand-receptor pairs in the progenitor niche (Related to Main Figure 5).

bioRxiv preprint doi: https://doi.org/10.1101/2025.06.10.656791; this version posted June 12, 2025. The copyright holder for this preprint (which was not certified by peer review) is the author/funder, who has granted bioRxiv a license to display the preprint in perpetuity. It is made available under aCC-BY 4.0 International license.

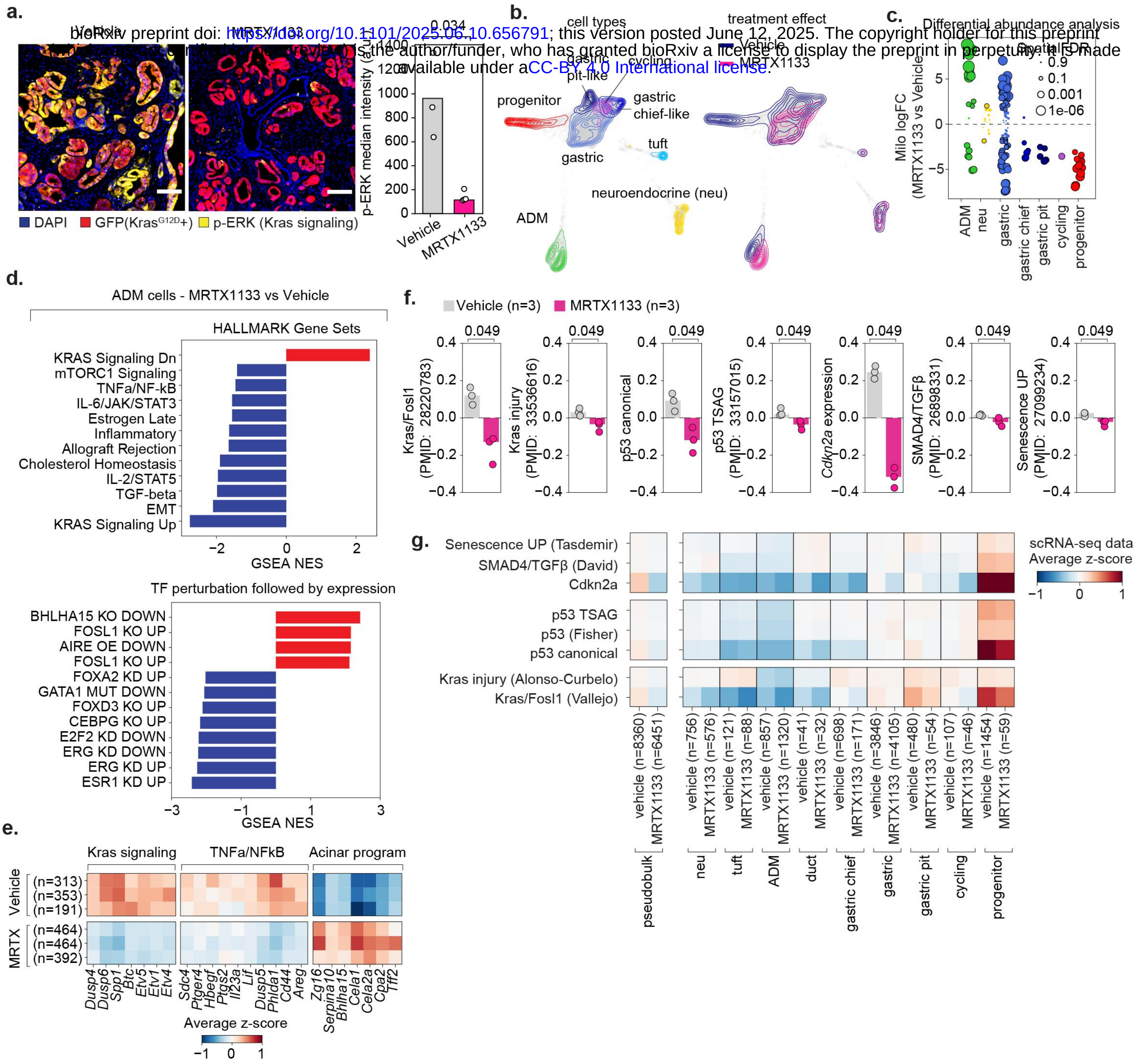

Supplementary Figure S9. Molecular and compositional changes in the premalignant pancreas upon acute oncogenic Kras inhibition (Related to Main Figure 6).

bioRxiv preprint doi: https://doi.org/10.1101/2025.06.10.656791; this version posted June 12, 2025. The copyright holder for this preprint (which was not certified by peer review) is the author/funder, who has granted bioRxiv a license to display the preprint in perpetuity. It is made available under aCC-BY 4.0 International license.

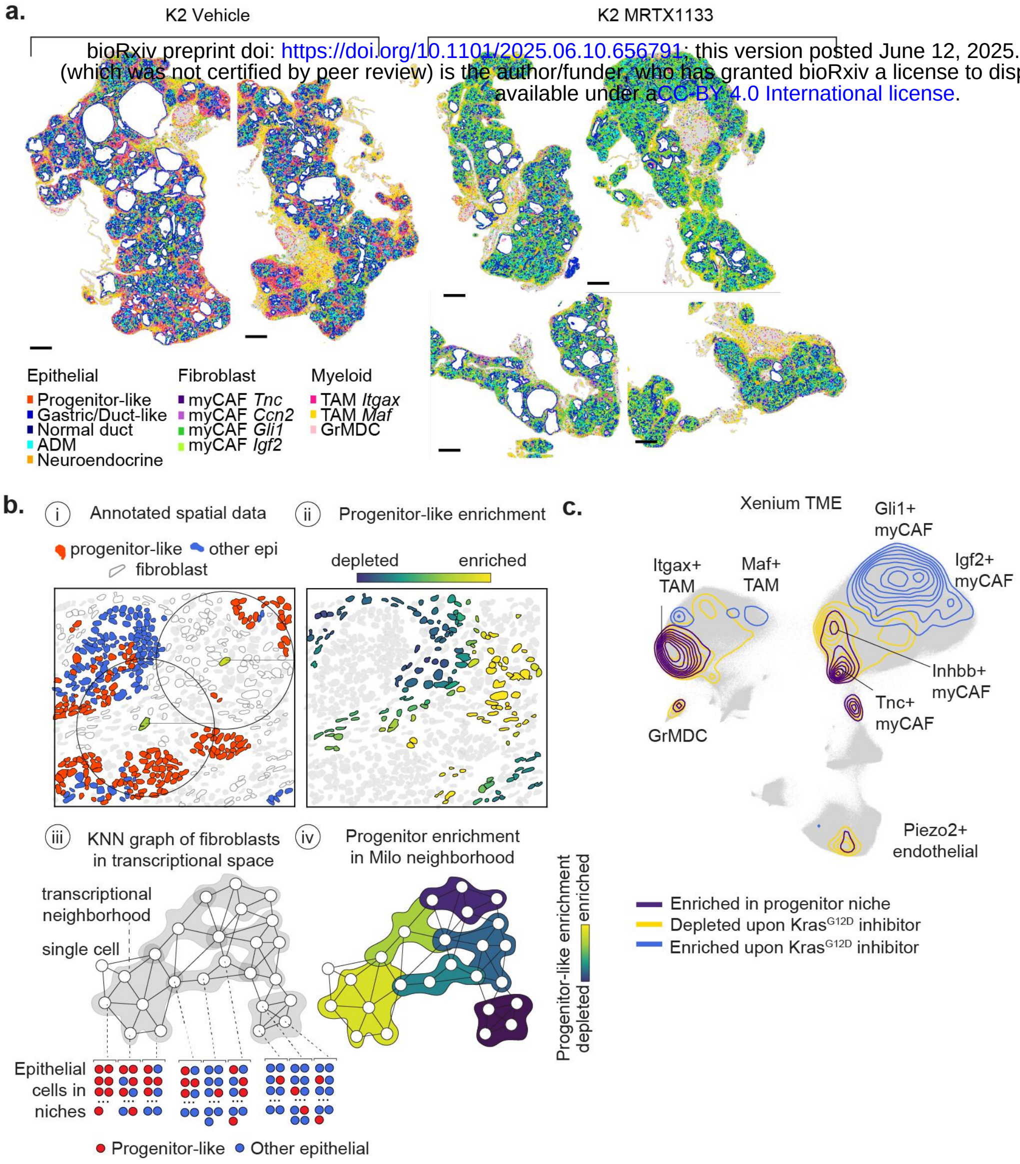

Supplementary Figure S10. Molecular and compositional changes in the premalignant pancreas upon acute oncogenic Kras inhibition (Related to Main Figure 6).

bioRxiv preprint doi: https://doi.org/10.1101/2025.06.10.656791; this version posted June 12, 2025. The copyright holder for this preprint (which was not certified by peer review) is the author/funder, who has granted bioRxiv a license to display the preprint in perpetuity. It is made available under aCC-BY 4.0 International license.

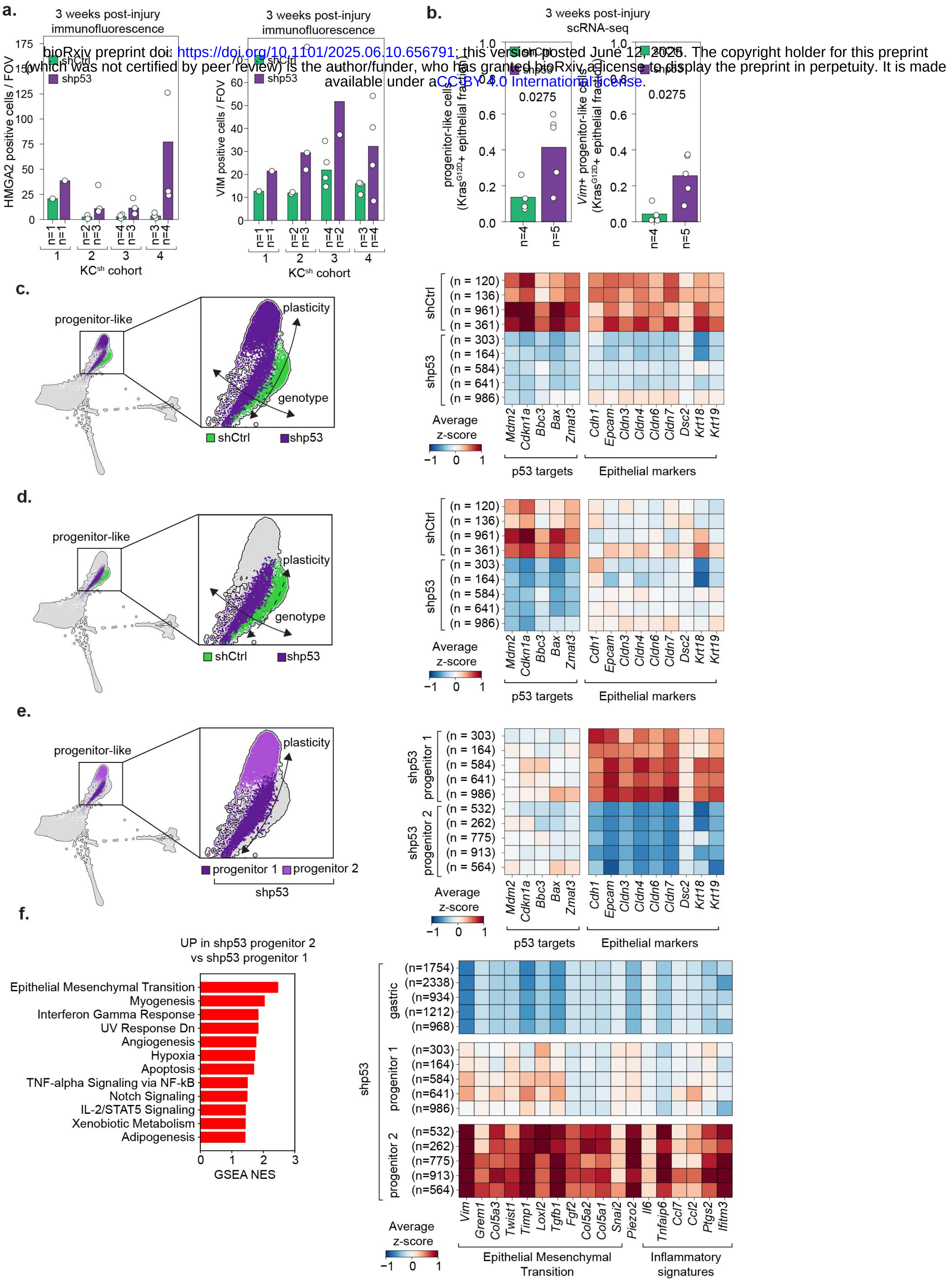

Supplementary Figure S11. Transcriptional consequences of p53 loss in the context of oncogenic Kras activation and pancreatic injury (Related to Main Figure 7).

bioRxiv preprint doi: https://doi.org/10.1101/2025.06.10.656791; this version posted June 12, 2025. The copyright holder for this preprint (which was not certified by peer review) is the author/funder, who has granted bioRxiv a license to display the preprint in perpetuity. It is made available under aCC-BY 4.0 International license.

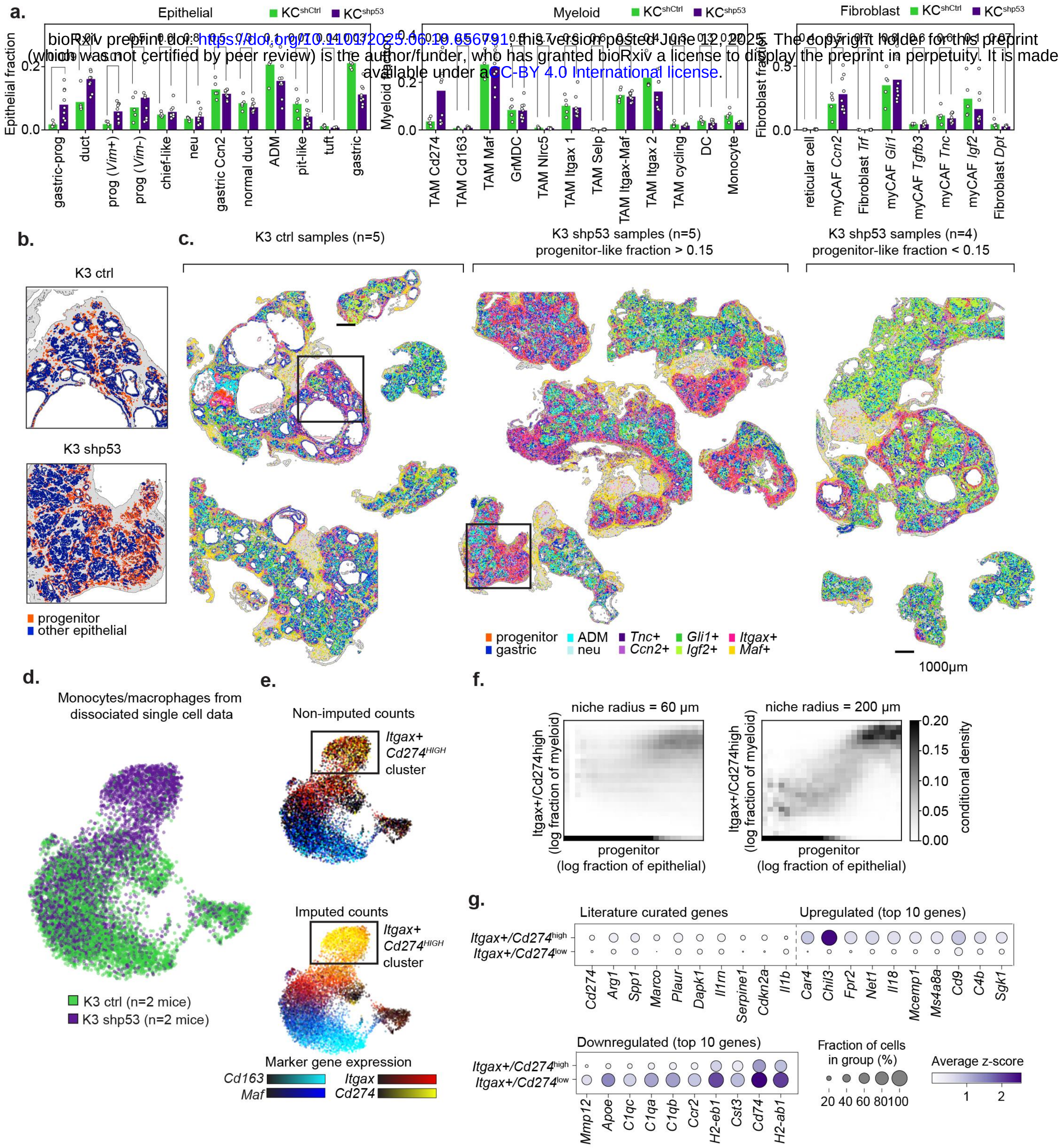

Supplementary Figure S12. Tissue remodeling upon p53 knockdown in the context of oncogenic Kras and pancreatic injury (Related to Main Figure 7).

bioRxiv preprint doi: https://doi.org/10.1101/2025.06.10.656791; this version posted June 12, 2025. The copyright holder for this preprint (which was not certified by peer review) is the author/funder, who has granted bioRxiv a license to display the preprint in perpetuity. It is made available under aCC-BY 4.0 International license.

Table S1. Metadata of scRNA-seq produced in this study

sample_id	condition	Mouse strain	Source	Hash id	batch	cell count	Counts per cell (mean)	Detected genes per cell (mean)	10x version	Related figures
254_2RH_PanINPM_GFPh	Pre-tumor p53 proficient 1	KPLOH	This work	unhashed	2	3,365	16018.8	3394.7	v2	Fig1 Fig2
254_2RH_PanINPM_Kate_CD45_1_31	Pre-tumor p53 deficient 1	KPLOH	This work	unhashed	2	88	24659.8	4055.1	v2	Fig1 Fig2
254_RH_PanINPM_GFPh	Pre-tumor p53 proficient 2	KPLOH	This work	unhashed	2	3,194	14828.8	3226.6	v2	Fig1 Fig2
254_RH_PanINPM_Kate_CD45_1_4	Pre-tumor p53 deficient 2	KPLOH	This work	unhashed	2	558	29470.9	4567.4	v2	Fig1 Fig2
53_LHRH_PDACreg_KATE	Tumor p53 deficient 7	KPLOH	This work	unhashed	2	2,791	27471.2	4529.8	v2	Fig1 Fig2
53_NH_PanINPM_GFPh	Pre-tumor p53 proficient 0	KPLOH	This work	unhashed	2	2,585	17438.3	3368.7	v2	Fig1 Fig2
9268_PHLH_PDAC_SP	Tumor p53 deficient 8	KPLOH	This work	unhashed	2	2,600	26649.4	3991.1	v2	Fig1 Fig2
9268_RH_PanINPM_DP	Pre-tumor p53 proficient 3	KPLOH	This work	unhashed	2	1,503	17330.8	3446.4	v2	Fig1 Fig2
Ag-Lung-Mets-Kate	Tumor p53 deficient 10	KPC (p53R172H)	PMID: 37167403	unhashed	1	313	52329.3	5582.6	v2	Fig1 Fig2
Ag-PDAC-PT-Kate	Tumor p53 deficient 10	KPC (p53R172H)	PMID: 37167403	unhashed	1	1,982	28501.5	4136.7	v2	Fig1 Fig2
DAC_D020_p5_Epi	Tumor p53 deficient 11	KPfC	PMID: 37167403	unhashed	1	2,036	33921.8	4312.8	v2	Fig1 Fig2
DACC963_mKate_plus	Tumor p53 deficient 9	KPfC	PMID: 37167403	unhashed	1	1,491	36368.8	4655.5	v2	Fig1 Fig2
DACC963LIVERmet	Tumor p53 deficient 9	KPfC	PMID: 37167403	unhashed	1	1,701	37370.6	4762.5	v2	Fig1 Fig2
DACC963PT_Kate_plus	Tumor p53 deficient 9	KPfC	PMID: 37167403	unhashed	1	275	37863.3	4567.3	v2	Fig1 Fig2
PDAC-SP3	Tumor p53 deficient 6	KPLOH	This work	unhashed	2	2,869	24434.5	3809.4	v2	Fig1 Fig2
pre_dp_2	Pre-tumor p53 proficient 5	KPLOH	This work	unhashed	8	2,076	21085.8	3786.5	v3	Fig1 Fig2
pre_sp_2	Pre-tumor p53 deficient 5	KPLOH	This work	unhashed	8	212	74262.0	6335.4	v3	Fig1 Fig2
Preclinical_DP_batch1	Pre-tumor p53 proficient 4	KPLOH	This work	A0301	7	344	8150.3	2308.9	v3	Fig1 Fig2
Preclinical_DP_batch1	Pre-tumor p53 proficient 4	KPLOH	This work	A0302	7	5,708	9682.7	2545.5	v3	Fig1 Fig2

bioRxiv preprint doi: https://doi.org/10.1101/2025.06.10.656791; this version posted June 12, 2025. The copyright holder for this preprint (which was not certified by peer review) is the author/funder, who has granted bioRxiv a license to display the preprint in perpetuity. It is made available under aCC-BY 4.0 International license.

Preclinical_DP_batch1	Pre-tumor p53 proficient 4	KPLOH	This work	undefined	7	247	17517.8	2054.1	v3	Fig1 Fig2
Preclinical_SP_batch1	Pre-tumor p53 deficient 4	KPLOH	This work	A0301	7	10	13578.9	3044.5	v3	Fig1 Fig2
Preclinical_SP_batch1	Pre-tumor p53 deficient 4	KPLOH	This work	A0302	7	590	22675.5	4108.6	v3	Fig1 Fig2
Preclinical_SP_batch1	Pre-tumor p53 deficient 4	KPLOH	This work	undefined	7	14	79336.1	5400.3	v3	Fig1 Fig2
p489c_shRen_p53exp_caer_3weeks	control Injury, 3 weeks	KCshCtrl	This work	B0301	3	1,718	13095.7	3137.3	v3	Fig7
p489c_shRen_p53exp_caer_3weeks	control Injury, 3 weeks	KCshCtrl	This work	B0302	3	1,608	13598.9	3222.2	v3	Fig7
p489c_shRen_p53exp_caer_3weeks	control Injury, 3 weeks	KCshCtrl	This work	undefined	3	221	27018.1	2794.2	v3	Fig7
shRen_caer_3weeks_repeat	control Injury, 3 weeks	KCshCtrl	This work	B0304	5	3,742	13175.0	3246.6	v3	Fig7
shRen_caer_3weeks_repeat	control Injury, 3 weeks	KCshCtrl	This work	B0305	5	2,840	14041.6	3309.8	v3	Fig7
shRen_caer_3weeks_repeat	control Injury, 3 weeks	KCshCtrl	This work	undefined	5	867	29102.1	2213.6	v3	Fig7
p489c_shp53_caer_3weeks	p53 knockdown, Injury, 3 weeks	KCshp53	This work	B0303	3	3,034	9459.1	2725.9	v3	Fig7
p489c_shp53_caer_3weeks	p53 knockdown, Injury, 3 weeks	KCshp53	This work	B0304	3	3,225	9414.4	2711.1	v3	Fig7
p489c_shp53_caer_3weeks	p53 knockdown, Injury, 3 weeks	KCshp53	This work	undefined	3	359	17922.5	2117.9	v3	Fig7
shp53_caer_3weeks_repeat	p53 knockdown, Injury, 3 weeks	KCshp53	This work	B0304	5	2,513	12226.5	3195.0	v3	Fig7
shp53_caer_3weeks_repeat	p53 knockdown, Injury, 3 weeks	KCshp53	This work	B0305	5	2,965	11728.2	3084.0	v3	Fig7
shp53_caer_3weeks_repeat	p53 knockdown, Injury, 3 weeks	KCshp53	This work	B0306	5	2,593	12316.6	3210.1	v3	Fig7
shp53_caer_3weeks_repeat	p53 knockdown, Injury, 3 weeks	KCshp53	This work	undefined	5	222	30944.2	2667.9	v3	Fig7
JR-2281_Krasi_caer_epi	p53 proficient, MRTX1133	KPLOH	This work	B0304	9	2283	14560.0	3145.1	v3	FigS9
JR-2281_Krasi_caer_epi	p53 proficient, MRTX1133	KPLOH	This work	B0305	9	1654	14762.2	3025.1	v3	FigS9
JR-2281_Krasi_caer_epi	p53 proficient, MRTX1133	KPLOH	This work	B0306	9	2514	13502.7	3155.1	v3	FigS9
JR-2281_Krasi_caer_epi	p53 proficient, MRTX1133	KPLOH	This work	Undefined	9	48	5927.0	1441.3	v3	FigS9

bioRxiv preprint doi: https://doi.org/10.1101/2025.06.10.656791; this version posted June 12, 2025. The copyright holder for this preprint (which was not certified by peer review) is the author/funder, who has granted bioRxiv a license to display the preprint in perpetuity. It is made available under aCC-BY 4.0 International license.

JR-2281_Ctrl_caer_epi	p53 proficient, Vehicle	KPLOH	This work	B0304	9	3062	13402.9	3078.8	v3	FigS9
JR-2281_Ctrl_caer_epi	p53 proficient, Vehicle	KPLOH	This work	B0305	9	2618	13152.1	3017.2	v3	FigS9
JR-2281_Ctrl_caer_epi	p53 proficient, Vehicle	KPLOH	This work	B0306	9	2680	14234.8	3207.8	v3	FigS9
JR-2281_Ctrl_caer_epi	p53 proficient, Vehicle	KPLOH	This work	Undefined	9	26	7430.4	2074.7	v3	FigS9
JR-sp-28-02	control, 3 weeks post-injury	KCshp53 (dox off)	This work	BC001	Flex1	21,181	2641.0	924.9	FLEX	FigS7 FigS12
JR-sp-17-01	p53 knockdown, 3 weeks post-injury	KCshp53 (dox on)	This work	BC003	Flex1	18,593	2236.0	1102.2	FLEX	FigS7 FigS12
JR-sp-19-09	control, 3 weeks post-injury	KCshRen (dox on)	This work	BC002	Flex1	17,098	1520.0	850.9	FLEX	FigS7 FigS12
JR-sp-28-07	p53 knockdown, 3 weeks post-injury	KCshp53 (dox on)	This work	BC004	Flex1	16,443	2647.8	980.7	FLEX	FigS7 FigS12

bioRxiv preprint doi: https://doi.org/10.1101/2025.06.10.656791; this version posted June 12, 2025. The copyright holder for this preprint (which was not certified by peer review) is the author/funder, who has granted bioRxiv a license to display the preprint in perpetuity. It is made available under aCC-BY 4.0 International license.

Table S2. smFISH probe metadata (Related to Fig. S5)

filename	codename	date	Gene symbol	Readout id	channel	readout_probe	Number readout	Num probes
LOH20210524_GFP_RS0015_x2_opool.fa	LOH20210524	20210524	GFP	RS0015	Cy5	ATCCTCCTTCAATACATCCC	4	28
LOH20210524_RIKALLELE_RS0083_x0_opool.fa	LOH20210524	20210524	RIKALLELE	RS0083	Cy7	ACACTACCACCATTTCCTAT	1	89
LOH20210524_PTPRC_RS0095_x1_opool.fa	LOH20210524	20210524	PTPRC	RS0095	Cy7	ACTCCACTACTACTCACTCT	2	92
LOH20210524_CDKN1A_RS0109_x0_opool.fa	LOH20210524	20210524	CDKN1A	RS0109	Cy5	ACCCTCTAACTTCCATCACA	1	82
Almu20211213_PanchoIFN_ITGAX_RS0109_x0_opool.fa	Almu20211213_PanchoIFN	20211213	ITGAX	RS0109	Cy5	ACCCTCTAACTTCCATCACA	1	92
LOH20210524_VIM_RS0175_x0_opool.fa	LOH20210524	20210524	VIM	RS0175	Cy5	ACCACAACCCATTCCTTTCA	1	86
LOH20210524_ANXA10_RS0237_x1_opool.fa	LOH20210524	20210524	ANXA10	RS0237	Cy7	TTTCTACCACTAATCAACCC	2	56
LOH20210622_CDKN2A_RS0247_x2_opool.fa	LOH20210622	20210622	CDKN2A	RS0247	Cy5	ACCCTTTACAAACACACCCT	4	30
Pancreas20220830_CassandraDirena_revisions_highExpressors_SPP1_RS0247_x2_opool.fa	Pancreas20220830_CassandraDirena_revisions_highExpressors	20220830	SPP1	RS0247	Cy5	ACCCTTTACAAACACACCCT	2	55
LOH20210524_MUC6_RS0247_x1_opool.fa	LOH20210524	20210524	MUC6	RS0247	Cy5	ACCCTTTACAAACACACCCT	2	92
LOH20210622_KRT19_RS0255_x1_opool.fa	LOH20210622	20210622	KRT19	RS0255	Cy7	TCCTATTCTCAACCTAACCT	2	67
LOH20220302_newMalignancyMarkers_RBPJL_RS0307_x0_opool.fa	LOH20220302_newMalignancyMarkers	20220302	RBPJL	RS0307	Cy7	TATCCTTCAATCCCTCCACA	1	88
LOH20210524_CPA1_RS0332_x1_opool.fa	LOH20210524	20210524	CPA1	RS0332	Cy5	ACATTACACCTCATTCTCCC	2	70
LOH20210622_MKI67_RS0384_x0_opool.fa	LOH20210622	20210622	MKI67	RS0384	Cy7	TTCTCCCTCTATCAACTCTA	1	92
LOH20230507_microenvironment_nreadout_2_spacer_A_PLAUR_ENSMUST00000002284_RS0406_possible_oligos.fasta	LOH20230507_microenvironment	20230507	PLAUR	RS0406	Cy5	ACCCTTACTACTACATCATC	2	45
LOH20230608_progenitor_nreadout_1_spacer_A_HMGA2_ENSMUST00000159699_RS0406_possible_oligos.fasta	LOH20230608_progenitor	20230608	HMGA2	RS0406	Cy5	ACCCTTACTACTACATCATC	1	100
LOH20211213_OIS_TFF1_RS0451_x2_opool.fa	LOH20211213_OIS	20211213	TFF1	RS0451	Cy7	TCCTAACAACCAACTACTCC	4	28
Senescence20210901_PECAM1_RS0468_x0_opool.fa	Senescence20210901	20210901	PECAM1	RS0468	Cy7	TCTATCATTACCCTCCTCCT	1	92

bioRxiv preprint doi: https://doi.org/10.1101/2025.06.10.656791; this version posted June 12, 2025. The copyright holder for this preprint (which was not certified by peer review) is the author/funder, who has granted bioRxiv a license to display the preprint in perpetuity. It is made available under aCC-BY 4.0 International license.

Pancreas20220830_CassandraDirena_revisions_highExpressors_FN1_RS0468_x1_opool.fa	Pancreas20220830_CassandraDirena_revisions_highExpressors	20220830	FN1	RS0468	Cy7	TCTATCATTACCCTCCTCCT	1	92
LOH20230507_microenvironment_nreadout_3_spacer_A_LCN2_ENSMUST00000192241_RS0468_possible_oligos.fasta	LOH20230507_microenvironment	20230507	LCN2	RS0468	Cy7	TCTATCATTACCCTCCTCCT	3	39
LOH20211213_OIS_LIF_RS0548_x0_opool.fa	LOH20211213_OIS	20211213	LIF	RS0548	Cy5	TATTCACCTTACAAACCCTC	1	92
LOH20230515_microenvironment_nreadout_1_spacer_A_TNC_ENSMUST00000107372_RS0548_possible_oligos.fasta	LOH20230515_microenvironment	20230515	TNC	RS0548	Cy5	TATTCACCTTACAAACCCTC	1	100
Senescence20210901_ACTA2_RS0578_x0_opool.fa	Senescence20210901	20210901	ACTA2	RS0578	Cy3B	AAACACACACTAAACCACCC	1	92
LOH20230608_progenitor_nreadout_1_spacer_A_ONECUT2_ENSMUST00000175965_RS0578_possible_oligos.fasta	LOH20230608_progenitor	20230608	ONECUT2	RS0578	Cy3B	AAACACACACTAAACCACCC	1	100
LOH20210622_MDM2_RS0584_x1_opool.fa	LOH20210622	20210622	MDM2	RS0584	Cy3B	AACTCATCTCAATCCTCCCA	2	89
LOH20230608_progenitor_nreadout_1_spacer_A_ITGB4_ENSMUST00000106461_RS0584_possible_oligos.fasta	LOH20230608_progenitor	20230608	ITGB4	RS0584	Cy3B	AACTCATCTCAATCCTCCCA	1	100
LOH20210622_BAX_RS0639_x2_opool.fa	LOH20210622	20210622	BAX	RS0639	Cy3B	TATCTCATCAATCCCACACT	4	32
Kal20221005_TGFB1_RS0639_x2_opool.fa	Kal20221005	20221005	TGFB1	RS0639	Cy3B	TATCTCATCAATCCCACACT	2	56
Senescence20210901_ADGRE1_RS0708_x0_opool.fa	Senescence20210901	20210901	ADGRE1	RS0708	Cy3B	TCCAACTCATCTCTAATCTC	1	92
LOH20210628_MSN_RS0730_x0_opool.fa	LOH20210628	20210628	MSN	RS0730	Cy3B	AATACTCTCCCACCTCAACT	1	92
LOH20220302_newMalignancyMarkers_PIEZO2_RS0763_x0_opool.fa	LOH20220302_newMalignancyMarkers	20220302	PIEZO2	RS0763	Cy3B	ATAAATCATTCCCACTACCC	1	92
LOH20230608_progenitor_nreadout_2_spacer_A_F3_ENSMUST00000029771_RS0793_possible_oligos.fasta	LOH20230608_progenitor	20230608	F3	RS0793	Cy3B	ACCCAACACTCATAACATCC	2	54
LOH20220411_CCN2_RS1047_x0_opool.fa	LOH20220411	20220411	CCN2	RS1047	Cy3B	ACCTTTCTCCATACCCAACT	1	84

bioRxiv preprint doi: https://doi.org/10.1101/2025.06.10.656791; this version posted June 12, 2025. The copyright holder for this preprint (which was not certified by peer review) is the author/funder, who has granted bioRxiv a license to display the preprint in perpetuity. It is made available under aCC-BY 4.0 International license.

Table S3. Embedding of TME subsets in injury induced tumorigenesis dissociated scRNA-seq (Related to Fig. S7 and Fig. S12)

object id	Parent object	Filter	Subpopulation filter	# cells in subset	# PCs	Explained variance	Related figure
all_cells	NA	control and shp53 samples	NA	73,315	100	53%	Intermediate processing
fibroblast_ctrl	all cells	control samples	fibroblasts (Dpt, Col1a1, Pdgfra, Pdpn)	9,576	86	36%	Intermediate processing
iCAF_myCAF_ctrl	fibroblast_ctrl	control samples	iCAF and myCAFs based on public signatures (PMID: 31197017)	8,915	88	36%	Intermediate processing
myCAF_ctrl	iCAF_myCAF_ctrl	control samples	myCAFs based on public signatures (PMID: 31197017)	6,291	83	32%	Fig. S7d
myeloid_ctrl	all cells	control samples	Public signature: (PMID: 35427180) Ptprc, Csf1r, Adgre1, H2-Ab1, Cd68, Lyz2, Itgam, Mertk	5,538	76	39%	Intermediate processing
myeloid_Maf_Itgax_ctrl	myeloid_ctrl	control samples	PhenoGraph clusters expressing either Maf or Itgax	4,505	87	42%	Fig. S7e
myeloid_all	all cells	control and shp53 samples	Public signature: (PMID: 35427180) Ptprc, Csf1r, Adgre1, H2-Ab1, Cd68, Lyz2, Itgam, Mertk	10,446	61	34%	Fig. S12

bioRxiv preprint doi: https://doi.org/10.1101/2025.06.10.656791; this version posted June 12, 2025. The copyright holder for this preprint (which was not certified by peer review) is the author/funder, who has granted bioRxiv a license to display the preprint in perpetuity. It is made available under aCC-BY 4.0 International license.

Table S5. Xenium sample information and embeddings (Related to Fig. 3, Fig. 4, Fig. 5, Fig. 6, Fig. 7).

xenium batch	slide id	slide name	mouse id	mouse strain	treatment	timepoint	Counts per cell	Num genes detected per cell
JR-2885	0004279_Region_1	JR0025	JR-sp-04-03	KCshCtrl	injury	2d post-injury	81.6	56.4
JR-2885	0004279_Region_2	JR0104_section 1	JR-sp-12-23	KPLOH	injury	1d post-injury	118.3	68.5
JR-2885	0004279_Region_3	JR0104_section 2	JR-sp-12-24	KPLOH	injury	1d post-injury	112.1	66.4
JR-2885	0004329_Region_1	JR0077	JR-sp-09-05	KCshCtrl	injury	3w post-injury	103	62.5
JR-2885	0004329_Region_2	JR0033	JR-sp-04-02	KCshCtrl	injury	3w post-injury	100.4	60.6
JR-2885	0004329_Region_3	JR0081	JR-sp-09-03	KCshp53	injury	3w post-injury	112.4	65.1
JR-3090	0011178_Region_1	JR-sp-15-04	JR-sp-15-04	KPLOH	injury + MRTX1133	2d post inhibitor	95.3	58.3
JR-3090	0011178_Region_2	JR-sp-15-09	JR-sp-15-09	KPLOH	injury + MRTX1133	2d post inhibitor	97.4	58.8
JR-3090	0011181_Region_1	JR-sp-15-06	JR-sp-15-06	KPLOH	injury + MRTX1133	2d post inhibitor	82.8	52.7
JR-3090	0011181_Region_2	JR-sp-15-02	JR-sp-15-02	KPLOH	injury + MRTX1133	2d post inhibitor	96.2	58.6
JR-2918	0015409_Region_1	JR0079	JR-sp-09-01	KCshp53	injury	3w post-injury	96.9	59.9
JR-2918	0015409_Region_2	JR0026	JR-sp-04-07	KCshp53	injury	2d post-injury	93.7	59.4
JR-2918	0015409_Region_3	JR0080	JR-sp-09-02	KCshp53	injury	3w post-injury	91.9	57.4
JR-2918	0015409_Region_4	JR0034	JR-sp-04-10	KCshp53	injury	3w post-injury	97.1	59.2
JR-2918	0015409_Region_5	JR0027	JR-sp-04-08	KCshp53	injury	2d post-injury	93.2	60.4
JR-2918	0015410_Region_1	DACE616	DACE616	KCshCtrl	injury	2d post-injury	92.5	59.5
JR-2918	0015410_Region_2	JR0078	JR-sp-09-06	KCshCtrl	injury	3w post-injury	84.4	53.5
JR-2918	0015410_Region_3	JR0024	JR-sp-04-01	KCshCtrl	injury	2d post-injury	81.3	55.7
JR-2918	0015410_Region_4	DACE617	DACE617	KCshCtrl	injury	2d post-injury	95	60.4
JR-3083	0027845_Region_1	JR0039	JR-sp-06-09	KCshCtrl	injury	1d post-injury	106.4	62.5
JR-3083	0027845_Region_2	JR0040	JR-sp-06-10	KCshCtrl	injury	1d post-injury	115.5	66.6
JR-3083	0027845_Region_3	JR-sp-15-05	JR-sp-15-05	KPLOH	injury + Vehicle	2d post vehicle	113.3	65
JR-3083	0027846_Region_1	JR0042	JR-sp-06-04	KCshp53	injury	1d post-injury	84.5	54.1
JR-3083	0027846_Region_2	JR0041	JR-sp-06-03	KCshp53	injury	1d post-injury	113.5	65.7
JR-3083	0027846_Region_3	JR-sp-15-07	JR-sp-15-07	KPLOH	injury + Vehicle	2d post vehicle	112	65.4
JR-3177	0028094_Region_1	JR-sp-19-09	JR-sp-19-09	KCshCtrl	injury	3w post-injury	88.5	57.5
JR-3177	0028094_Region_2	JR-sp-19-10	JR-sp-19-10	KCshCtrl	injury	3w post-injury	95.6	59.5
JR-3433	0042536_Region_1	JR-sp-28-25	JR-sp-28-25	KCshp53	injury	3w post-injury	78	52.7

bioRxiv preprint doi: https://doi.org/10.1101/2025.06.10.656791; this version posted June 12, 2025. The copyright holder for this preprint (which was not certified by peer review) is the author/funder, who has granted bioRxiv a license to display the preprint in perpetuity. It is made available under aCC-BY 4.0 International license.

JR-3433	0042536_Region_2	JR-sp-28-08	JR-sp-28-08	KCshp53	injury	3w post-injury	84.8	54.6
JR-3311	0042569_Region_3	JR0019	JR-sp-02-17	KPLOH	Spontaneous	pre-tumor	60.2	43.4
JR-3433	0042724_Region_1	JR-sp-17-01	JR-sp-17-01	KCshp53	injury	3w post-injury	80.6	52.5
JR-3433	0042724_Region_2	JR-sp-28-07	JR-sp-28-07	KCshp53	injury	3w post-injury	85.4	55.4
JR-3433	0042724_Region_3	JR-sp-28-06	JR-sp-28-06	KCshp53	injury	3w post-injury	89.2	57.1

bioRxiv preprint doi: https://doi.org/10.1101/2025.06.10.656791; this version posted June 12, 2025. The copyright holder for this preprint (which was not certified by peer review) is the author/funder, who has granted bioRxiv a license to display the preprint in perpetuity. It is made available under aCC-BY 4.0 International license.

Table S6. Compartment- and condition-specific embeddings for Xenium samples.

Compartment	Conditions included	Number of samples included	Number of genes	Fraction of genes	Number of PCs	Variance explained	Total cells	Related figures
TME	Non-perturbed samples	15	415	0.86	127	75%	6,489,818	Fig. S6
Epithelial	Non-perturbed samples	15	394	0.82	100	75%	3,217,585	All Xenium
Immune Myeloid	Non-perturbed samples	15	389	0.81	98	75%	1,844,178	All Xenium
Immune Lymphoid	Non-perturbed samples	15	385	0.8	87	75%	636,172	All Xenium
Fibroblast	Non-perturbed samples	15	387	0.8	100	75%	2,862,709	All Xenium
Mural Cells	Non-perturbed samples	15	369	0.76	85	75%	285,767	All Xenium
Endothelial	Non-perturbed samples	15	380	0.79	104	75%	814,550	All Xenium
Epithelial - control samples	Non-perturbed samples	15	399	0.83	96	75%	1,388,199	Fig. 3 and Fig. 4
Progenitor and gastric-like cells - control samples	Non-perturbed samples	15	392	0.81	93	75%	838,581	Fig. 3, Fig. 4, Fig. 5, Fig. S4, Fig. S8
Kras inhibitor experiment TME	MRTX1133 or vehicle treated samples	6	390	0.81	108	75%	1,216,079	Fig. 6, Fig. S10
Kras inhibitor experiment Epithelial	MRTX1133 or vehicle treated samples	6	370	0.77	93	75%	925,367	Fig. 6, Fig. S10

bioRxiv preprint doi: https://doi.org/10.1101/2025.06.10.656791; this version posted June 12, 2025. The copyright holder for this preprint (which was not certified by peer review) is the author/funder, who has granted bioRxiv a license to display the preprint in perpetuity. It is made available under aCC-BY 4.0 International license.

Table S7. Statistics on mixed cell states in Xenium data (Related to Fig. S6)

Mix type	Number of cells	% in dataset
mixed_myeloid_stroma	166,979	1.7
mixed_epithelial_stroma	70,906	0.72
mixed_endothelial_immue_stroma	33,375	0.34
mixed_lymphoid_other	22,790	0.23
mixed_mural_fibroblast	8,310	0.08
mixed_lymphoid_myeloid	3,338	0.03
mixed_mural_immune	2,750	0.02
mixed_mural_other	1,233	0.012

bioRxiv preprint doi: https://doi.org/10.1101/2025.06.10.656791; this version posted June 12, 2025. The copyright holder for this preprint (which was not certified by peer review) is the author/funder, who has granted bioRxiv a license to display the preprint in perpetuity. It is made available under aCC-BY 4.0 International license.

Table S8. Gene censoring in Xenium data (Related to Fig. 4, Fig. 5, Fig. S7, Fig. S8)

Compartment	Fraction positive threshold (dissociated)	Number of genes that passed dissociated threshold	Fraction positive threshold in any subpopulation	Number of genes that passed dissociated threshold	Number of genes that passed both thresholds
Epithelial	1% of cells	373	0.0794	326	318
Immune myeloid	1% of cells	296	0.0797	233	211
Fibroblast	1% of cells	347	0.1199	294	277
Immune B	1% of cells	225	0.0789	251	194
Immune T,NK,ILC	1% of cells	259	0.1184	220	194
Mural cells	1% of cells	304	0.0797	242	227
Endothelial cells	1% of cells	322	0.0788	273	258

bioRxiv preprint doi: https://doi.org/10.1101/2025.06.10.656791; this version posted June 12, 2025. The copyright holder for this preprint (which was not certified by peer review) is the author/funder, who has granted bioRxiv a license to display the preprint in perpetuity. It is made available under aCC-BY 4.0 International license.

Note S1

Construction of the diffusion operator and diffusion maps

Throughout this work, we make use of diffusion maps as a latent representation of single-cell expression data that is analogous to a non-linear version of principal components. A diffusion map captures dominant directions of diffusion along a single-cell manifold, understood as a random walk in which cells are allowed to reversibly transition between similar transcriptional states. We follow the computation of diffusion maps as previously described[1–3].

Let $M^{n\times m}$ be a matrix representing the log-normalized expression of m genes in n cells. We define the k-nearest neighbor graph (kNN) $G^{n\times n}$ as a sparse matrix such that $G_{i,j}$ is the Euclidean distance in principal component space between $cell_i$ and $cell_j$ when $cell_j$ belongs to the k-nearest neighbors of $cell_i$, and $G_{i,j} = 0$ for every other $cell_j$ in the data.

We compute the cell–cell affinity matrix $A^{n\times n}$ by applying an adaptive Gaussian kernel to the kNN matrix:

$$A_{i,j} = e^{\frac{-G_{i,j}}{h}}$$

where h varies for each $cell_i$ and is defined as the distance to its k' closest cell (we use $k' = 10$ for a kNN graph with $k = 30$). The width h of the Gaussian kernel determines the rate at which $A_{i,j}$ decays as a function of distance. Thus, an adaptive width allows controlling for heterogeneity in cell density along the single cell manifold.

To compute the diffusion operator $T^{n\times n}$, we symmetrize the cell-cell affinity matrix, set the diagonal to 0, and normalize by row, resulting in a row stochastic matrix. $T_{i,j}$ can be interpreted as the transition probability from $cell_i$ to $cell_j$. Further exponentiation of the diffusion operator results is diffusion, a random walk over the kNN graph that results in long-range connectivities between single cells based on short-scale phenotypic transitions.

Although Euclidean distances on PC space captures cell–cell similarities at the local level, and is routinely used to construct a kNN graph during manifold estimation, they fail to capture distances over long ranges due to non-linearities in the phenotypic manifold. Diffusion distance—intuitively understood as the result of a diffusion process or a random walk over the kNN graph—captures long-range cell-cell connectivities while respecting such non-linearities. A diffusion map results from the eigen-decomposition of the diffusion operator. The right eigenvectors of such decomposition, termed diffusion components, provide a new representation of the data that can be used to approximate diffusion distance. Ordering eigenvectors by their corresponding eigenvalue, and keeping the top L eigenvectors allows estimation of the diffusion distance between two cells:

$$D_{i,j} = \sum_{l=1}^{L} \lambda_l^{2t}\left(\psi_{il} - \psi_{jl}\right)^2$$

Where λ_l is the top l^{th} eigenvalue of the diffusion operator, $\psi_{\cdot l}$ it's associated eigenvector, and t is the number of diffusion steps ($t = 3$ in our analysis). Because the 0th right eigenvector of the

bioRxiv preprint doi: https://doi.org/10.1101/2025.06.10.656791; this version posted June 12, 2025. The copyright holder for this preprint (which was not certified by peer review) is the author/funder, who has granted bioRxiv a license to display the preprint in perpetuity. It is made available under aCC-BY 4.0 International license.

diffusion operator is a constant vector, we exclude this from the diffusion map, following work from Haghverdi and colleagues[3].

REFERENCES

1. Setty, M. *et al.* Characterization of cell fate probabilities in single-cell data with Palantir. *Nat Biotechnol* **37**, 451–460 (2019).
2. van Dijk, D. *et al.* Recovering Gene Interactions from Single-Cell Data Using Data Diffusion. *Cell* **174**, 716–729.e27 (2018).
3. Haghverdi, L., Buettner, F. & Theis, F. J. Diffusion maps for high-dimensional single-cell analysis of differentiation data. *Bioinformatics* **31**, 2989–2998 (2015).
